# Physiologically Based Pharmacokinetic Modeling of Bupropion and Its Metabolites in a CYP2B6 Drug-Drug-Gene Interaction Network

**DOI:** 10.3390/pharmaceutics13030331

**Published:** 2021-03-04

**Authors:** Fatima Zahra Marok, Laura Maria Fuhr, Nina Hanke, Dominik Selzer, Thorsten Lehr

**Affiliations:** Clinical Pharmacy, Saarland University, 66123 Saarbrücken, Germany; fatima.marok@uni-saarland.de (F.Z.M.); laura.fuhr@uni-saarland.de (L.M.F.); n.hanke@mx.uni-saarland.de (N.H.); dominik.selzer@uni-saarland.de (D.S.)

**Keywords:** physiologically based pharmacokinetic modeling, bupropion, hydroxybupropion, cytochrome P450 2B6 (CYP2B6), drug-drug-interactions (DDIs), drug-gene-interactions (DGIs)

## Abstract

The noradrenaline and dopamine reuptake inhibitor bupropion is metabolized by CYP2B6 and recommended by the FDA as the only sensitive substrate for clinical CYP2B6 drug–drug interaction (DDI) studies. The aim of this study was to build a whole-body physiologically based pharmacokinetic (PBPK) model of bupropion including its DDI-relevant metabolites, and to qualify the model using clinical drug–gene interaction (DGI) and DDI data. The model was built in PK-Sim^®^ applying clinical data of 67 studies. It incorporates CYP2B6-mediated hydroxylation of bupropion, metabolism via CYP2C19 and 11β-HSD, as well as binding to pharmacological targets. The impact of CYP2B6 polymorphisms is described for normal, poor, intermediate, and rapid metabolizers, with various allele combinations of the genetic variants CYP2B6*1, *4, *5 and *6. DDI model performance was evaluated by prediction of clinical studies with rifampicin (CYP2B6 and CYP2C19 inducer), fluvoxamine (CYP2C19 inhibitor) and voriconazole (CYP2B6 and CYP2C19 inhibitor). Model performance quantification showed 20/20 DGI ratios of hydroxybupropion to bupropion AUC ratios (DGI AUC_HBup/Bup_ ratios), 12/13 DDI AUC_HBup/Bup_ ratios, and 7/7 DDGI AUC_HBup/Bup_ ratios within 2-fold of observed values. The developed model is freely available in the Open Systems Pharmacology model repository.

## 1. Introduction

Bupropion is used for the treatment of major depressive disorders and to support smoking cessation [1]. Nearly one out of 10 prescriptions among psychotherapeutics was attributed to bupropion in 2018 [2]. In the treatment of depressive disorders, it is either used as monotherapy or in combination with other antidepressant agents, and is administered as oral immediate release, sustained release or extended release tablets [1,3].

Bupropion and various of its metabolites are pharmacologically active [4]. Hydroxybupropion is one of the major metabolites and is formed by cytochrome P450 (CYP) 2B6-mediated hydroxylation of bupropion. Bupropion and hydroxybupropion are known inhibitors of dopamine and noradrenaline reuptake transporters. Furthermore, they act as antagonists to various acetylcholine receptors and serotonin reuptake transporters [5,6,7]. Erythrohydrobupropion and threohydrobupropion are further metabolites of bupropion and formed via 11β-hydroxysteroid-dehydrogenase (11β-HSD) as the rate-limiting step in the reaction pathway [8]. After administration of a single dose of 200 mg bupropion, nearly 97% of total bupropion was recovered in urine (87%) and feces (10%). However, only 0.5% of unchanged bupropion was found in urine [9]. Adverse drug events or symptoms of bupropion intoxications, i.e., insomnia, vomiting, dry mouth or seizures, can be attributed to bupropion and its metabolites [10]. It was observed that erythrohydrobupropion plasma levels significantly correlate with insomnia, while threohydrobupropion is assumed to be responsible for dry mouth [11]. Moreover, hydroxybupropion induced seizures more potently than bupropion in rodent experiments [12] and immediate release administration of bupropion was associated with a higher incidence of seizures than a sustained release administration in humans [10,13,14]. All three metabolites are further metabolized via glucuronidation by uridine 5′-diphospho-glucuronosyltransferase (UGT) 2B7 [15].

According to the United States Food and Drug Administration (FDA), bupropion is listed as a sensitive substrate of CYP2B6 in clinical drug–drug interaction studies (DDIs), ref. [16] and it is subject to various CYP2B6 DDIs, when inducers or inhibitors of CYP2B6 are administered concomitantly [17,18]. For example, HIV patients that are on bupropion medication exhibited a 57% decrease in bupropion AUC at the initiation of antiviral therapy with ritonavir, an inducer of CYP2B6 [19,20]. Clopidogrel, an antiplatelet drug and known CYP2B6 inhibitor, was reported to decrease hydroxybupropion AUC by 60% after pretreatment with 75 mg clopidogrel [21]. Even short-term use of CYP2B6 perpetrator drugs can seriously affect bupropion hydroxylation, as shown for the administration of fluvoxamine and voriconazole shortly before bupropion administration that caused a 90% reduction in the hydroxybupropion to bupropion AUC plasma ratio (AUC_HBup/Bup_) [22]. In addition to its CYP2B6 interaction potential, bupropion is also listed as a strong clinical inhibitor of CYP2D6 [16]. However, the inhibitory effect is primarily attributed to its metabolites hydroxybupropion, erythrohydrobupropion, and threohydrobupropion [23].

In addition to its DDI potential, bupropion is also subject to CYP2B6 drug–gene interactions (DGIs). Polymorphisms in the *CYP2B6* gene can result in rapid, normal, intermediate, or poor metabolizer phenotypes. Important genetic variants of *CYP2B6* include CYP2B6*1, *4, *5, and *6 with frequencies of 49%, 4%, 12%, and 23% in European populations, respectively [17,24]. It has been shown that hydroxybupropion plasma levels and hydroxybupropion to bupropion plasma ratios are significantly altered in rapid or poor metabolizers, with 153% higher or 31% lower hydroxybupropion to bupropion AUC ratios compared to wildtype [17]. However, the clinical relevance for CYP2B6 polymorphic patients is still unclear and dose adjustment guidelines have yet to be developed.

Considering the DDI, DGI and drug–drug–gene interaction (DDGI) potential, the complex pharmacokinetics of bupropion should be thoroughly investigated. Here, physiologically based pharmacokinetic modeling (PBPK) can be a valuable tool to grasp the high level of complexity and implications of genetic polymorphisms and perpetrator drugs on the pharmacokinetics of bupropion [25]. However, a robust bupropion PBPK model connected to a strong DDGI CYP2B6 network has not been developed yet.

The aim of the presented work was the development of a PBPK model of bupropion including its three most relevant metabolites for the prediction of CYP2B6 DDI, DGI, and DDGI scenarios, and the qualification of this model using clinical data of CYP2B6 polymorphic individuals and DDI studies with different perpetrator drugs in the first published CYP2B6 DDGI network. The final model is shared with the modeling and drug development community in the Open Systems Pharmacology model repository (www.open-systems-pharmacology.org, December 2020) [26]. A transparent and comprehensive documentation of model development and evaluation is provided in the Supplementary Materials.

## 2. Materials and Methods

### 2.1. Software

The PBPK model was developed with the open-source modeling software PK-Sim^®^ and MoBi^®^ (Open Systems Pharmacology Suite 9.1, released under the GPLv2 license by the Open Systems Pharmacology community, www.open-systems-pharmacology.org (accessed on 31 December 2020)) [26]. GetData Graph Digitizer 2.26.0.20 (© S. Fedorov) was used to digitize published clinical study data according to best practices [27]. Model input parameters were optimized by application of the Levenberg–Marquardt algorithm with multiple starting values. Local sensitivity analyses were performed within PK-Sim^®^. Non-compartmental analyses, model performance measures, and plots were compiled in R 3.6.3 (The R Foundation for Statistical Computing, Vienna, Austria) with RStudio 1.2.5033 (RStudio PBC, Boston, MA, USA).

### 2.2. Clinical Data

Clinical studies of bupropion in single-and multiple-dose regimens were gathered and digitized from the literature [27]. The collected profiles were divided into a training (*n* = 19) and a test dataset (*n* = 48), used for model building and model evaluation, respectively [3,17,18,19,20,21,22,28,29,30,31,32,33,34,35,36,37,38,39,40,41,42,43,44,45,46,47,48,49,50,51,52,53,54,55,56,57]. Studies in the training dataset were selected to include metabolite concentration-time profiles, a wide dosing range, and different oral formulations. To minimize bias, the distribution of data on female and male populations was balanced as well. The whole dataset is documented in the clinical study tables, with their respective clinical data shown in semilogarithmic as well as linear plots in Sections 2–4 of the Supplementary Materials.

### 2.3. PBPK Model Building

Model building was started with an extensive literature search for physicochemical properties and information regarding absorption, distribution, metabolism, and excretion (ADME) processes of bupropion and its investigated metabolites.

Averaged demographic information about age, sex, ethnicity, body weight, and height listed in clinical study reports was used to create virtual individuals. If data on demographics was missing, a virtual standard individual with default values was created. Details on standard individuals are listed in Table S1.2 of the Supplementary Materials. Virtual populations of 500 individuals were created based on the demographic information provided in the clinical study reports. If no data was available, a male European population with an age distribution of 20–50 years was assumed.

Tissue distribution of enzymes and binding proteins used for the ADME processes of bupropion and its metabolites was implemented according to the PK-Sim^®^ expression database [58]. Information on their expression is provided in Table S1.1 of the Supplementary Materials.

Tablet formulations with different bupropion release kinetics were simulated using a Weibull model (Section 1.1 in the Supplementary Materials). The Weibull shape and Weibull time parameters (50% dissolved) were derived, if available, from dissolution profiles reported in the literature. Model parameters that either could not be sufficiently informed from the literature or were involved in important QSAR model estimates of permeability and distribution processes were optimized by fitting the model simultaneously to all plasma concentration-time profiles of the training dataset.

### 2.4. PBPK Model Evaluation

PBPK model evaluation was performed using several methods. Model predictions of plasma concentration-time profiles were graphically compared to observed profiles from the respective clinical studies. Subsequently, predicted plasma concentrations from all studies were plotted against their corresponding observed values in goodness-of-fit plots. The model performance was further evaluated by comparison of predicted to observed area under the plasma concentration-time curve (AUC) and maximum plasma concentration (C_max_) values. AUC values (predicted as well as observed) were calculated from the time of drug administration to the time of the last concentration measurement (AUC_last_). If measured profiles were missing, predicted AUC was calculated as reported in the corresponding study. As quantitative measures of the model performance, mean relative deviation (MRD) of all predicted and observed plasma concentrations and geometric mean fold error (GMFE) of all predicted and observed AUC_last_ and C_max_ values were calculated according to Equations (1) and (2). Predictions with MRD and GMFE values ≤ 2 were considered successful model predictions.

(1) $${\mathrm{MRD}=10}^{x};\;\;\;x=\sqrt{\frac{\sum_{i=1}^{k}{\;(\log}_{10}{ĉ}_{i}{-\log}_{10}c_{i}{)}^{2}}{k}}$$

where c_i_ = the ith observed plasma concentration, ĉ_i_ = the corresponding predicted plasma concentration, and k = the number of observed values.

(2) $${\mathrm{GMFE}=10}^{x};\;\;\;x=\frac{\sum_{i=1}^{m}{\;|\;\log}_{10}\;\left(\frac{{\mathrm{predicted}\;\mathrm{PK}\;\mathrm{parameter}}_{i}}{{\mathrm{observed}\;\mathrm{PK}\;\mathrm{parameter}}_{i}}\right)|\;}{m}$$

where predicted PK parameter_i_ = the ith predicted AUC_last_ or C_max_ value, observed PK parameter_i_ = the corresponding observed AUC_last_ or C_max_ value, and *m* = the number of studies.

Local sensitivity of the AUC of bupropion, hydroxybupropion, erythrohydrobupropion, and threohydrobupropion to single parameter changes was analyzed for bupropion multiple dose administrations of the three different release formulations. Analyses included parameters that were either optimized or assumed to have an impact on AUC. A detailed description is provided in the Supplementary Materials Section 2.5.5.

### 2.5. DGI, DDI and DDGI Modeling

To model the effect of CYP2B6 genetic variants, difference in enzyme activity was expressed by variation of the Michaelis-Menten (K_M_) and catalytic rate constant (kcat) values for CYP2B6*1/*1 (wildtype), CYP2B6*1|*4, CYP2B6*1|*6, CYP2B6*5|*5, and CYP2B6*6|*6 genotypes. Parameters that could not be informed from literature were optimized by fitting the model to clinical data based on a population with the respective genotype. If no data on genotype or phenotype of the investigated subjects was available, *CYP2B6* wildtype was assumed. If mean plasma concentration-time profiles of different genotypes were reported, the most frequent one was used for model simulations.

To model the effect of DDIs, different interaction processes (competitive inhibition or induction) were incorporated into the perpetrator PBPK model with the corresponding in vitro interaction parameters values extracted from the literature. The different interaction types are described in the Supplementary Materials Section 1.6. Previously published PBPK models of the CYP2B6 perpetrator drugs rifampicin, voriconazole, and fluvoxamine were used to simulate DDI scenarios with bupropion [59,60,61].

To predict the rifampicin-bupropion DDGIs in carriers of different *CYP2B6* alleles, inhibition and induction parameters for wildtype DDI simulations were assumed.

### 2.6. DGI, DDI and DDGI Model Evaluation

DGI, DDI, and DDGI model performance was evaluated by comparison of predicted to observed plasma concentration-time profiles of bupropion (Bup) and its CYP2B6 metabolite hydroxybupropion (HBup) after single administration and during concomitant administration of CYP2B6 perpetrator drugs (rifampicin or fluvoxamine and voriconazole). In addition, the metabolite–parent ratio HBup/Bup of the PK parameters AUC (AUC_last_ or AUC_inf_ [AUC extrapolated to infinity]) and C_max_ was calculated for predicted and observed effect and control profiles according to Equation (3). HBup/Bup AUC and C_max_ ratios were used to calculate DGI, DDI, and DDGI effect ratios according to Equation (4).

(3) $${\mathrm{PK}}_{\mathrm{HBup}/\mathrm{Bup}}=\frac{{\mathrm{HBup}\;}_{\mathrm{PK}}}{{\mathrm{Bup}\;}_{\mathrm{PK}}}$$

where Bup _PK_ = PK parameter of bupropion, and HBup _PK_ = PK parameter of hydroxybupropion.

(4) $${\mathrm{DGI},\;\mathrm{DDI}\;\mathrm{or}\;\mathrm{DDGI}\;\mathrm{PK}}_{\mathrm{HBup}/\mathrm{Bup}}=\frac{{\mathrm{PK}}_{\mathrm{HBup}/\mathrm{Bup}}\;(\mathrm{DGI},\;\mathrm{DDI}\;\mathrm{or}\;\mathrm{DDGI})}{{\;\mathrm{PK}}_{\mathrm{HBup}/\mathrm{Bup}}\;(\mathrm{reference})}$$

where PK_HBup/Bup_ = Hydroxybupropion-bupropion PK parameter ratio.

As a quantitative measure of DGI, DDI, and DDGI model performance, *GMFE* values of the predicted and observed PK_Hbup/Bup_ values as well as PK_Hbup/Bup_ effect ratios were calculated according to Equation (2).

## 3. Results

### 3.1. Model Building and Evaluation

A PBPK model for bupropion and its three metabolites, hydroxybupropion, erythrohydrobupropion, and threohydrobupropion was developed. A total of 48 clinical studies in which bupropion was administered in a wide dosing range (20–300 mg) as immediate, sustained, and extended release tablets in single or multiple dose regimens were used in the model development process. In total, all 48 studies included plasma concentration-time profiles of bupropion, 40 of hydroxybupropion, and 17 of erythro- and threohydrobupropion. Study details are listed in Table S2.1 of the Supplementary Materials. For the extended release tablet formulation, Weibull parameters were calculated from dissolution profiles from the literature according to Langenbucher et al. [62]. For additional formulations, parameters were fitted to plasma concentration-time profiles of the training dataset. Dissolution details are listed in Table S2.2 of the Supplementary Materials.

**Figure 1:**
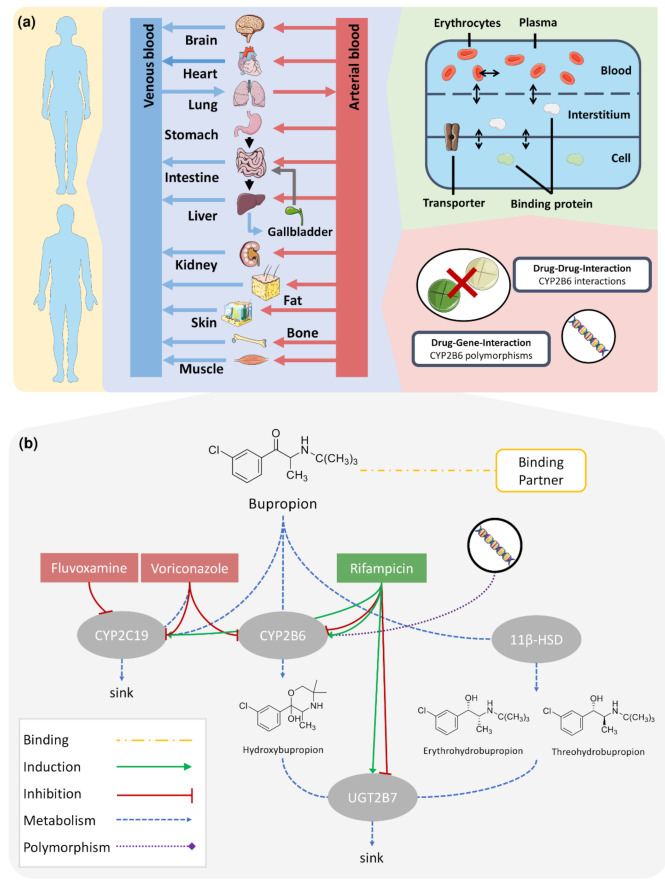
Modeling overview of the CYP2B6 DDGI Network. A whole-body PBPK model was augmented for the simulation of CYP2B6 drug–drug, drug–gene, and drug–drug–gene interactions (**a**). The model describes bupropion’s metabolism via CYP2B6, CYP2C19, and 11β-HSD (**b**). Its metabolites hydroxybupropion, erythro- and threohydrobupropion are transformed via UGT2B7. Binding to an unspecific binding protein, representing bupropion’s pharmacological targets, was implemented. Several effects on the bupropion PK were modeled, i.e., effects of genetic polymorphisms on CYP2B6; induction of CYP2C19 by rifampicin; induction and inhibition of CYP2B6 and UGT2B7 by rifampicin; inhibition of CYP2C19 by fluvoxamine; and inhibition of CYP2B6 and CYP2C19 by voriconazole. Drawings by Servier, licensed under CC BY 3.0 [68]. 11β-HSD: 11β-hydroxysteroid-dehydrogenase, CYP2B6: cytochrome P450 2B6, CYP2C19: cytochrome P450 2C19, UGT2B7: uridine 5′-diphospho-glucuronosyltransferase 2B7.

Figure 1a illustrates the basic structure of the developed whole-body PBPK model and implemented DGI and DDI processes. Figure 1b summarizes the implemented metabolism of bupropion via CYP2B6 to hydroxybupropion and via 11β-HSD to erythro- and threohydrobupropion. Moreover, CYP2B6 metabolism is influenced by genetic variants and perpetrator drugs, such as the CYP2B6 inducer and inhibitor rifampicin [63,64] and the CYP2B6 inhibitor voriconazole [65]. Bupropion metabolism via CYP2C19 was modeled to reflect minor metabolic pathways of bupropion covered by other CYPs. Since binding to therapeutic targets might influence the PK of bupropion, binding to a surrogate protein representing various neurotransmitter transporters was implemented. Furthermore, the model applied glucuronidation of the three metabolites via UGT2B7. UGT2B7 and CYP2C19 metabolism were also considered in DDI predictions, as fluvoxamine and voriconazole inhibit CYP2C19 [65,66] and rifampicin induces CYP2C19. Moreover, both compounds induce and inhibit UGT2B7 [64,67]. In summary, the simulated effects include: (i) CYP2B6 polymorphisms; (ii) induction of CYP2B6, CYP2C19 and UGT2B7, and inhibition of CYP2B6 and UGT2B7 by rifampicin; and (iii) inhibition of CYP2C19 by fluvoxamine as well as inhibition of CYP2B6 and CYP2C19 by voriconazole.

Table 1 and Table 2 provide an overview of the drug-dependent model parameters as well as details on the implemented metabolic processes. A description of all implemented processes and formulations with their respective model parameters is listed in the drug-dependent parameter table in the Supplementary Materials.

**Figure 2:**
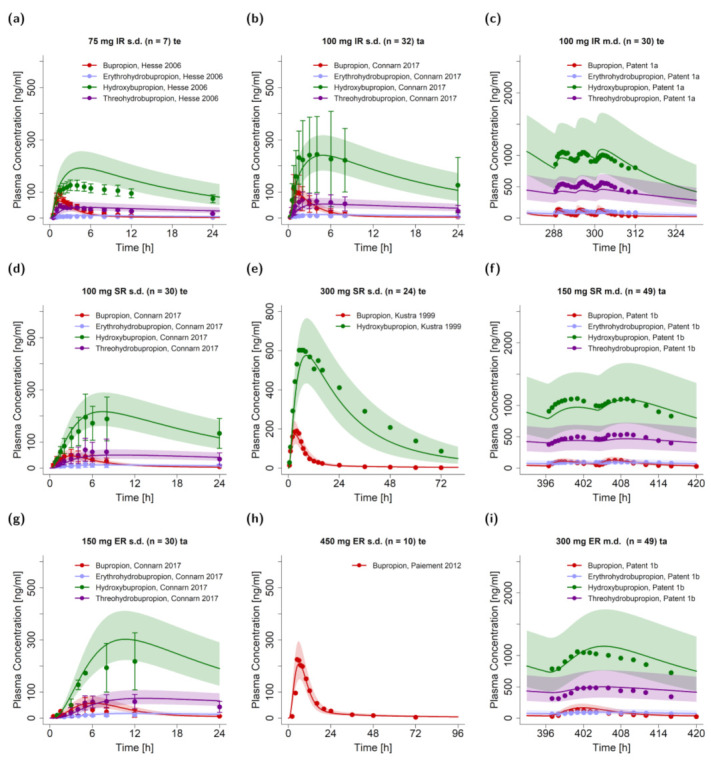
Predicted plasma concentration-time profiles of selected clinical studies from test and training datasets for bupropion, hydroxybupropion, erythro- and threohydrobupropion after application of single and multiple oral tablets with immediate release (**a**–**c**), sustained release (**d**–**f**) and extended release (**g**–**i**) kinetics compared to observed data [3,28,32,51,54]. The geometric means of the population predictions (*n* = 500) are shown as solid lines and corresponding observed data as dots (arithmetic mean ± standard deviation, if available). The shaded areas indicate the geometric standard deviation. Detailed information on study protocols is provided in Table S2.1 of the Supplementary Materials. ER: extended release, IR: immediate release, m.d.: multiple dose, n: number of participants, s.d.: single dose, SR: sustained release, ta: training dataset, te: test dataset.

Figure 2a–i presents simulations of bupropion administration as immediate, sustained, and extended release tablets. The bupropion PBPK model accurately described and predicted plasma concentration-time profiles of bupropion and its metabolites after single and multiple dose administrations for the three different formulations. Predicted concentration-time profiles of all 48 clinical studies compared to observed data are provided on linear and semi-logarithmic scale in Figures S2.4.1–S2.4.14 in the Supplementary Materials. All simulated plasma profiles were in good agreement with their respective observed data.

**Figure 3:**
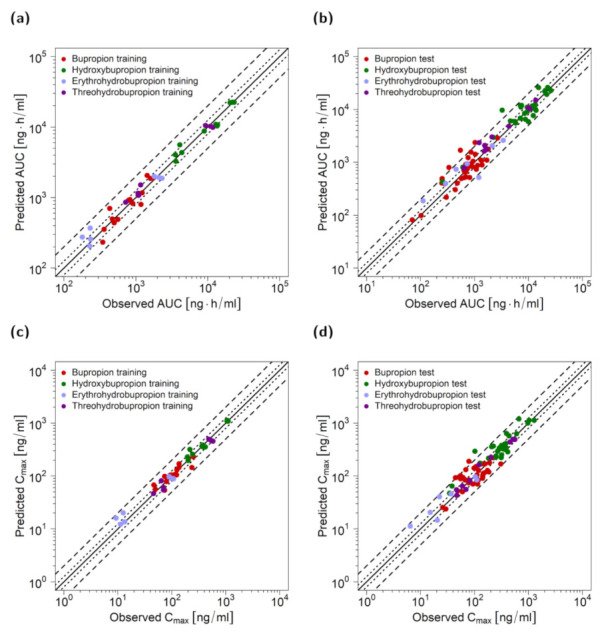
Goodness-of-fit plots of PK parameters for bupropion and metabolites. Predicted AUC of the training (**a**) and test dataset (**b**) as well as C_max_ values of the training (**c**) and test dataset (**d**) compared to observed values. The solid line marks the line of identity, dotted lines indicate 1.25-fold, and dashed lines indicate 2-fold deviation. AUC: area under the plasma concentration-time curve from the time of drug administration to the time of the last concentration measurement, C_max_: maximum plasma concentration.

Model performance is demonstrated in Figure 3 as comparisons of predicted to observed AUC_last_ (a) and C_max_ values (b). Both training and test data were well predicted for all four compounds. In addition, Table 3 provides MRDs of plasma concentration-time profiles and GMFEs of AUC_last_ and C_max_ for the four compounds. With 119/124 of the predicted AUC_last_ and 121/124 of the predicted C_max_ values within the 2-fold acceptance limits, total GMFEs of 1.31 (range 0.43–3.06) for predicted AUC_last_ values and 1.29 (range 0.55–2.87) for C_max_ values further confirmed an adequate model performance. Individual MRD and GMFE values for all plasma profiles are listed in Tables S2.3 and S2.4 in the Supplementary Materials.

Sensitivity analysis of a 14-day multiple dose simulation of either 100 mg immediate release three times daily, 150 mg sustained release twice daily, or 300 mg extended release once daily, revealed that regardless of the bupropion formulation, the highest impact on bupropion AUC can be attributed to the fraction unbound of bupropion, a fixed literature value. Of the optimized parameters, the most impactful parameter was CYP2B6 kcat for immediate and sustained release formulations. For the extended release formulation, AUC was more sensitive to bupropion lipophilicity than to CYP2B6 kcat. A detailed assessment of model sensitivity is provided in Section 2.5.5 of the Supplementary Materials.

### 3.2. DGI Modeling and Evaluation

**Figure 4:**
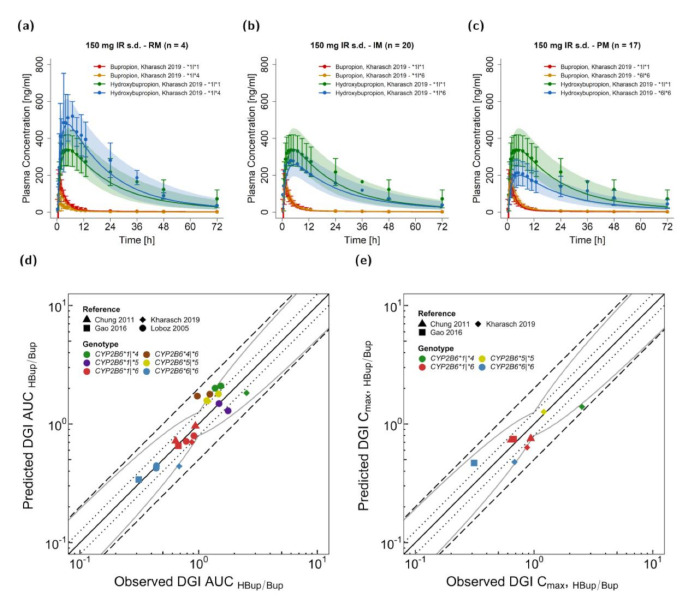
Bupropion CYP2B6 DGI model evaluation. Predicted compared to observed plasma concentration-time profiles are illustrated for CYP2B6*1/*4 (**a**), CYP2B6*1/*6 (**b**) and CYP2B6*6/*6 (**c**) genotypes [17] in comparison to the CYP2B6*1/*1 genotype (wildtype, *n* = 21). The effects of respective genetic variants of CYP2B6 are shown in orange and blue for bupropion and hydroxybupropion, respectively; the corresponding profiles of the CYP2B6*1/*1 genotype are shown in red and green for bupropion and hydroxybupropion, respectively. The solid line illustrates the geometric mean of the population predictions (*n* = 500) and the shaded area the geometric standard deviation. Predicted compared to observed DGI effect ratios are shown for hydroxybupropion–bupropion ratios of AUC (**d**) and C_max_ (**e**) with different colors indicating the genotypes and different shapes the respective studies [17,35,40,44]. The straight solid line marks the line of identity, the curved solid lines show the prediction acceptance limits proposed by Guest et al. including 1.25-fold variability [85]. Dotted lines indicate 1.25-fold and dashed lines indicate 2-fold deviation. Details on the study protocols and DGI ratios are provided in the Supplementary Materials. AUC: area under the plasma concentration-time curve, C_max_: maximum plasma concentration, DGI: drug–gene interaction, HBup/Bup: hydroxybupropion–bupropion ratio, IM: intermediate metabolizer, IR: immediate release, PM: poor metabolize, RM: rapid metabolizer, s.d.: single dose.

The developed model was extended to describe effects of polymorphism in the CYP2B6 gene on CYP2B6 activity and interaction with CYP2B6 perpetrator drugs. Most published studies only reported mean profiles of populations, often exhibiting multiple different genotypes, or only the respective AUC or HBup/Bup ratio of plasma AUC or single concentrations at specific time points after administration. However, plasma concentration-time profiles of four genetic variants could be gathered from the literature. These included: CYP2B6*1 (or wildtype), CYP2B6*4, CYP2B6*5, and CYP2B6*6. Three studies reporting profiles of bupropion and hydroxybupropion were used for development of DGI predictions. Michaelis Menten constants (K_M_) were obtained from the literature and corrected for binding in the microsomal assay, if necessary. The rate constant k_cat_ was optimized for the CYP2B6*6 haplotype. Table 1 provides bupropion K_M_ and k_cat_ values for the implemented *CYP2B6* alleles. Prediction of CYP2B6*4 heterozygous expression was simulated by splitting the implemented CYP2B6-mediated pathway in two clearance processes. In vitro parameters representing the CYP2B6 partition not produced by the CYP2B6*4 allele were assumed to be equal to parameters for homozygous expression of the respective allele (i.e., CYP2B6*1/*1 or CYP2B6*6/*6). For example, the CYP2B6*1 allele was simulated with a K_M_ value of 25.80 µmol/l from the literature and half of the optimized CYP2B6*1|*1 kcat value of 21.74 1/min. Figure 4 demonstrates the performance of the bupropion DGI model with Figure 4a–c illustrating model-based simulations of 150 mg bupropion as an immediate release tablet alongside their respective observed profiles of three different polymorphisms in comparison to CYP2B6*1|*1 (wildtype). The effect of DGIs, especially on hydroxybupropion plasma levels, was well described for rapid (CYP2B6*1|*4), normal (CYP2B6*1|*1 or wildtype), intermediate (CYP2B6*1|*6) and poor metabolizers (CYP2B6*6|*6). Plots documenting the model performance for all modeled bupropion DGIs are provided in Figures S3.4.1 and S3.4.2 of the Supplementary Materials. Figure 4d–e shows predicted compared to observed DGI HBup/Bup ratios calculated for AUC (d) and C_max_ (e). Predicted DGI HBup/Bup ratios were in good agreement with observed ratios, with 20/20 of DGI AUC_HBup/Bup_ and 8/8 of DGI C_max HBup/Bup_ values within the 2-fold acceptance limits and 18/20 of DGI AUC_HBup/Bup_ and 7/8 of DGI C_max HBup/Bup_ values within the prediction success limits suggested by Guest et al. with 1.25-fold variability [85]. Predicted and observed DGI AUC_HBup/Bup_ ratios showed an overall GMFE of 1.25 (range 0.64–1.77) and DGI C_max HBup/Bup_ of 1.35 (range 0.41–1.29). Tables S3.3 and S3.4 of the Supplementary Materials list all calculated MRD and GMFE values of predicted and observed plasma concentration-time profiles and the corresponding AUC and C_max_ values along with the DGI HBup/Bup ratios.

### 3.3. Bupropion DDI Modeling and Evaluation

The bupropion DDI model was established and evaluated using a total of five clinical DDI studies with the perpetrator drugs fluvoxamine together with voriconazole (one study) and rifampicin (four studies). Details on the previously developed PBPK models for rifampicin [60], fluvoxamine [61], and voriconazole [59] are listed in the parameter tables in Section 4 of the Supplementary Materials.

The rifampicin-bupropion DDI was predicted as an induction of CYP2B6, CYP2C19, and UGT2B7 with interaction parameters obtained from the literature [63,64,67]. Additionally, competitive inhibition of CYP2B6 and UGT2B7 by rifampicin was included as well [86,87].

The fluvoxamine-voriconazole-bupropion DDI was predicted as competitive inhibition of CYP2B6 and CYP2C19 metabolism by voriconazole and competitive inhibition of CYP2C19 by fluvoxamine. Literature CYP2C19 K_i_ values were used for both fluvoxamine and voriconazole [65,66]. CYP2B6 K_i_ of voriconazole was adjusted via parameter optimization.

**Figure 5:**
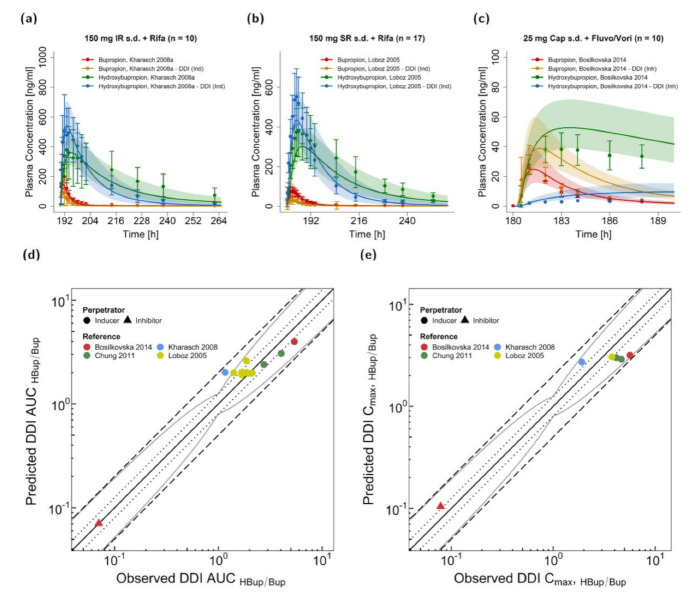
Victim drug plasma concentrations and DDI HBup/Bup ratios for AUC and C_max_ of the modeled bupropion DDIs. Predicted compared to observed plasma concentration-time profiles of bupropion and hydroxybupropion are shown for interactions with rifampicin (**a**,**b**) as well as fluvoxamine and voriconazole (**c**). The profiles during administration of CYP2B6 perpetrator drugs are shown in orange and blue for bupropion and hydroxybupropion, respectively, and the corresponding profiles without DDI are shown in red and green for bupropion and hydroxybupropion, respectively. The solid line illustrates the geometric mean of the population predictions (*n* = 500) and the shaded area the geometric standard deviation. Predicted compared to observed effect ratio plots for the hydroxybupropion–bupropion ratios of AUC (**d**) and C_max_ (**e**) show data of four CYP2B6 inducer and one CYP2B6 inhibitor studies. Different shapes indicate the perpetrators and different colors the respective studies [19,22,35,44]. The straight solid line marks the line of identity; the curved solid lines show the prediction acceptance limits proposed by Guest et al. including 1.25-fold variability [85]. Dotted lines indicate 1.25-fold and dashed lines indicate 2-fold deviation. Details on the study protocols and the values of all DDI ratios are provided in the Supplementary Materials. AUC: area under the plasma concentration-time curve, Cap: capsule (Geneva Capsule [22]), C_max_: maximum plasma concentration, DDI: drug-drug interaction, Fluvo/Vori: fluvoxamine and voriconazole, HBup/Bup: hydroxybupropion–bupropion ratio, Ind: inducer, Inh: inhibitor, IR: immediate release, Rifa: rifampicin, s.d.: single dose, SR: sustained release.

**Figure 6:**
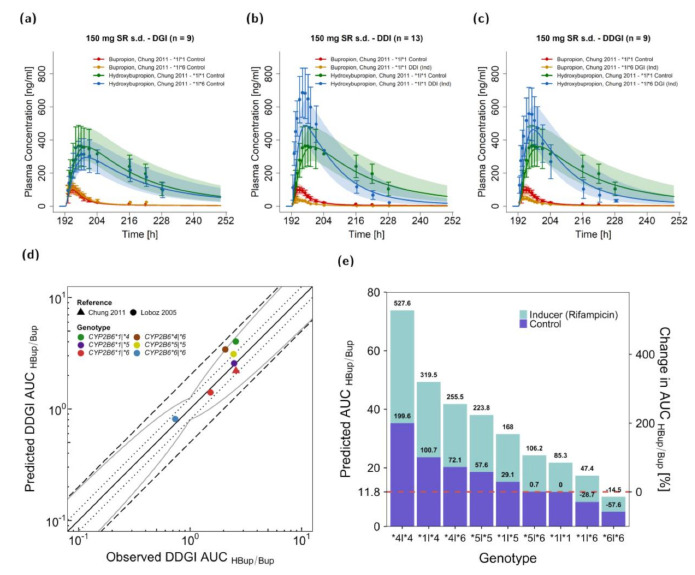
Rifampicin–bupropion CYP2B6 DDGI. Predicted compared to observed plasma concentration-time profiles are illustrated for the CYP2B6 bupropion DGI (CYP2B6*1|*6 compared to CYP2B6*1|*1) (**a**), for rifampicin–bupropion DDI in individuals with the CYP2B6*1|*1 genotype (**b**) and for the rifampicin–bupropion DDGI for individuals with the CYP2B6*1|*6 genotype compared to carriers of the CYP2B6*1|*1 genotype (**c**). The predicted and observed plasma concentrations under the combined effects of CYP2B6 genetic polymorphism and perpetrators are shown in orange and blue, respectively, while the control is shown in red and green. The solid line illustrates the geometric mean of the population predictions (*n* = 500) and the shaded area the geometric standard deviation. Predicted compared to observed effect ratio plot for the hydroxybupropion–bupropion ratio of AUC values are shown for six different genotypes after rifampicin induction (**d**). Different colors indicate the genotypes and different shapes the respective studies ([35,44]). The straight solid line marks the line of identity, the curved solid lines show the prediction acceptance limits proposed by Guest et al. including a 1.25-fold variability [85]. Dotted lines indicate 1.25-fold and dashed lines indicate 2-fold deviation. Predicted effects of rifampicin-bupropion DDGIs on the hydroxybupropion-bupropion AUC ratios were compared to CYP2B6*1|*1 without co-administration of rifampicin (**e**). Details on study protocols and DDGI ratios are provided in the Supplementary Materials. AUC area under the plasma concentration-time curve, DGI: drug–gene interaction, DDI: drug–drug interaction, DDGI: drug drug gene interaction, HBup/Bup: hydroxybupropion–bupropion ratio, Ind: inducer, po: oral, s.d.: single dose, SR: sustained release.

Figure 5a–c illustrates predicted plasma concentration-time profiles before and during DDI scenarios in comparison to the corresponding observed data from clinical DDI studies. Induction by rifampicin and inhibition via fluvoxamine and voriconazole are shown for bupropion and hydroxybupropion plasma levels on linear scale for three representative studies (two for rifampicin DDIs with different bupropion release formulations and one for fluvoxamine and voriconazole). In Section 4 of the Supplementary Materials, predicted compared to observed profiles of all investigated rifampicin–bupropion DDIs are presented. In the DDI studies, 600 mg rifampicin were administered daily with 150 mg bupropion given once either as immediate release (Figure 5a) or sustained release tablets (shown in Figure 5b, Figure 6 and in Section 4 of the Supplementary Materials). Plasma concentration-time profiles during CYP2B6 inhibition were only provided in one DDI study with a single dose of bupropion as a cocktail capsule [22]. For the fluvoxamine–voriconazole–bupropion DDI scenario, fluvoxamine and voriconazole were administered concomitantly, 2 h before the bupropion cocktail capsule. Further details on regimens and population characteristics of the DDI studies are listed in Tables S4.4 and S4.5 of the Supplementary Materials.

HBup/Bup ratios were calculated via Equations (4) and (5) for AUC and C_max_ values and are depicted in Figure 5d–e. Here, 12/13 DDI AUC_HBup/Bup_ and 6/6 DDI C_max HBup/Bup_ values were within the limits proposed by Guest et al. assuming 1.25-fold variability [85] with overall GMFE values of 1.23 (range 0.74–1.73) for DDI AUC_HBup/Bup_ and 1.46 (range 0.56–1.44) for DDI C_max HBup/Bup_. Calculated MRD and GMFE values of all predicted DDI studies are listed in Tables S4.6 and S4.7 of the Supplementary Materials.

### 3.4. Bupropion DDGI Modeling and Evaluation

DDGIs, combinations of DGIs and DDIs, were predicted for the polymorphisms CYP2B6*1, CYP2B6*4, CYP2B6*5, and CYP2B6*6 for bupropion intake in DDI scenarios with concomitant rifampicin administration. Rifampicin was administered in multiple oral doses of 600 mg (daily) before a single oral dose of 150 mg bupropion (sustained release) was administered [35,67]. One DDGI study reported plasma concentration-time profiles of bupropion and hydroxybupropion for CYP2B6*1|*1 and CYP2B6*1|*6 either with or without rifampicin. Figure 6a–c shows predicted compared to observed profiles for a DGI in CYP2B6*6 heterozygous subjects (a), a DDI with rifampicin in *CYP2B6 wildtype* subjects (b), and a DDGI with rifampicin in CYP2B6*6 heterozygous subjects (c) compared to *CYP2B6* wildtype subjects receiving bupropion solely. Furthermore, HBup/Bup ratios of AUC_inf_ after rifampicin induction were reported for several CYP2B6 polymorphic individuals [44]. Figure 6d illustrates predicted compared to observed DDGI AUC_HBup/Bup_ values for several genetic variants of *CYP2B6*.

Figure 6e shows predicted AUC_HBup/Bup_ values for all possible allele combinations of the modeled *CYP2B6* variants with or without rifampicin interaction. HBup/Bup ratios were decreased in carriers of the variant CYP2B6*6 allele. A homozygous CYP2B6*6 expression with inducer was predicted to be lower in CYP2B6 activity than wildtype CYP2B6 without inducer resulting in a ~15% decrease in AUC_HBup/Bup_. CYP2B6*5|*6 individuals were predicted to exhibit AUC_HBup/Bup_ ratios similar to wildtype individuals, with or without inducer. The highest AUC_HBup/Bup_ was simulated for homozygous expression of the gene variant CYP2B6*4 after rifampicin intake. However, it should be noted that for predictions of the genotypes CYP2B6*4|*4 and CYP2B6*5|*6, no observed data for validation were available. In summary, DDGI predictions showed overall DDGI AUC_HBup/Bup_ GMFE values of 1.27 (range 0.85–1.60) with 7/7 of the predicted DDGI AUC_HBup/Bup_ within the acceptance limits of Guest et al., assuming 1.25-fold variability [85].

## 4. Discussion

In the presented work, a whole-body PBPK model of bupropion and its metabolites hydroxybupropion, threohydrobupropion and erythrohydrobupropion was built and evaluated to predict drug plasma concentrations over a wide dosing range (20–300 mg) for three different oral formulations. Furthermore, the model was extended to describe the effects of CYP2B6 DGIs, DDIs, and rifampicin-bupropion CYP2B6 DDGIs on the PK of bupropion and its metabolites.

So far, only one other bupropion PBPK model has been published yet [88]. Despite demonstrating reasonable performance, in comparison to the presented work, the model did not incorporate a similarly large amount of data for building and evaluation and did not reflect the effects of different genetic alterations of CYP2B6. These shortcomings, which we consider as necessary elements to qualify the bupropion PBPK model as a part of the CYP2B6 network, were addressed in our model.

Bupropion is predominantly metabolized to hydroxybupropion in the liver and, to some extent, also in the gut [10]. Even though CYP2B6 hydroxylation plays a major role in the metabolism of bupropion, the implementation of CYP2C19 as minor metabolic pathway was important to sufficiently describe the data including DDIs and DGIs [17,77]. In addition to CYP2B6 and CYP2C19 metabolism, carbonyl reductases transform bupropion to erythrohydrobupropion and threohydrobupropion [10]. In the presented model, the metabolic pathway along several carbonyl reductases was reduced to the rate-limiting enzymatic step via 11β-HSD for the formation of erythrohydrobupropion and threohydrobupropion. Here, the respective K_M_ values for all implemented enzymes could be informed from the literature. After a single dose of 150 mg bupropion, the bupropion fractions metabolized were predicted as 58%, 28%, and 13% via CYP2B6, 11β-HSD, and CYP2C19, respectively (Figure S2.1.1 of the Supplementary Materials). Furthermore, the model predicts an extensive metabolism of bupropion (99%) after complete absorption with small fractions excreted unchanged to urine and feces (~1%), which is consistent with the literature [9]. Reported bupropion fractions metabolized varied with measurements from in vitro clearance data of 56% or 12% for hydroxybupropion formation and 40% or 68% for threohydrobupropion formation [8,89], which are in reasonable agreement with predicted values.

Bupropion shows affinity to a variety of therapeutic targets, such as numerous acetylcholine receptors and dopamine and noradrenaline reuptake transporters [10]. Target binding was incorporated into our model as it improved the description of the concentration-time profiles. To simplify the complex binding of bupropion to several targets, only binding to the noradrenaline reuptake transporter was implemented, as it covers the expression in all relevant organs, such as brain or gastro-intestinal tract, where noradrenaline and dopamine reuptake transporters, as well as nicotinic acetylcholine receptors are expected. The applied K_D_ value is in good agreement with literature values describing binding or inhibition of the different relevant targets [78,79,80].

The bupropion metabolism is especially sensitive to genetic polymorphisms in CYP2B6 [17]. Unfortunately, documentation on genetic polymorphisms of participants was poor in most clinical studies. Either mean profiles of mixed populations were presented or no genotype information was reported. Nevertheless, gene variants CYP2B6*1, *4, *5, and *6 were included in our model and described the available plasma concentration-time profiles of bupropion and hydroxybupropion sufficiently well. Dose adjustment guidelines for genetic polymorphisms have not been implemented yet. However, as hydroxybupropion might play an important role in the occurrence and onset of seizures after rapid bupropion absorption [10,12,13,14], the presented model could support a rational individualized CYP2B6 polymorphism-guided dose selection.

The presented DDI network includes interactions via CYP2B6, CYP2C19, and UGT2B7. The rifampicin–bupropion DDI covers the induction of CYP2B6, CYP2C19, and UGT2B7, with simultaneous competitive inhibition of CYP2B6 and UGT2B7. All rifampicin–bupropion interaction parameters were derived from the literature [45,46,47,48]. However, inhibition is relatively weak with inhibition constants (K_i_) of 118.50 µmol/L and 554.87 µmol/L for CYP2B6 and UGT2B7 [86,87] and presumably negligible; especially after multiple dose applications of rifampicin. For single dose administrations, rifampicin’s inhibitory activity on bupropion could not be evaluated, due to a lack of clinical bupropion DDI data.

Voriconazole is a known CYP2B6 inhibitor that displayed interactions with bupropion and efavirenz [74]. The reported K_i_ value of voriconazole was not strong enough to fully describe the observed in vivo effects. This seems reasonable, since the metabolite voriconazole N-oxide is also responsible for the inhibitory effect on CYP2B6 [90], but was not implemented in the published PBPK model [59]. Moreover, a polymorphism-dependent CYP2B6 inhibition of voriconazole was previously described for efavirenz hydroxylation, where lower K_i_ values were reported for CYP2B6*6 than for CYP2B6*1 [74]. In the DDI study conducted by Bosilkovska et al. [22], six of 10 subjects exhibited a CYP2B6*6 polymorphism, which could potentially explain a deviation in prediction. Due to the lack of relevant clinical data, the K_i_ value of voriconazole for CYP2B6 had to be optimized and could not be validated yet. Hence, further in vitro studies are needed to optimize and evaluate the voriconazole DDI. Inhibition of CYP2C19 was implemented, as fluvoxamine and voriconazole are listed as strong and weak inhibitors for CYP2C19 by the FDA, respectively [16]. As bupropion is also a known CYP2D6 inhibitor, we assumed that the inhibitory effect on CYP2D6-mediated fluvoxamine metabolism is negligibly small, as fluvoxamine is given 2 h prior to bupropion administration, and as bupropion’s strong CYP2D6 inhibition potential is predominately attributed to its metabolites and a CYP2D6 downregulation after long-term bupropion intake [91].

The model correctly predicted DDGI plasma profiles of bupropion co-administered with rifampicin in CYP2B6*6 heterozygous subjects. Furthermore, DDGI model performance was successfully evaluated by comparison of predicted and reported HBup/Bup AUC ratios. Subsequently, potential DDGIs were simulated for combinations of the genetic *CYP2B6* variants CYP2B6*1, CYP2B6*4, CYP2B6*5, and CYP2B6*6. The simulated scenarios illustrate the models’ potential to investigate the effect of DDGIs on bupropion and hydroxybupropion plasma levels. Whether the simulated DDGI combinations lead to the predicted changes in HBup/Bup ratios, especially the profound increase in rapid metabolizers receiving the CYP2B6 inducer rifampicin, should be carefully evaluated in clinical studies. Moreover, pharmacological implications of bupropion intake, with or without perpetrator, in CYP2B6 polymorphic patients, are still unclear. While clinical efficacy or tolerability can be correlated to plasma levels of bupropion or its metabolites, guidelines regarding bupropion dosing have yet to be established. Our presented model demonstrated its flexibility in simulations of various DDGI scenarios and can be applied to develop rational dosing recommendations for bupropion drug labeling or clinical study design.

## 5. Conclusions

A comprehensive parent-metabolite PBPK model of bupropion including whole-body PBPK models of bupropion and the metabolites hydroxybupropion, erythrohydrobupropion, and threohydrobupropion was developed. Bupropion pharmacokinetics were thoroughly described for tablets with different release formulations in single and multiple dose regimens. The established CYP2B6 network incorporates reliable prediction of DGIs with several polymorphisms, DDIs and DDGIs as combinations of DGIs and DDIs. A transparent and detailed documentation of the model development and performance further underlines the model quality. The final PBPK model files are freely available in the Open Systems Pharmacology repository (www.open-systems-pharmacology.org, December 2020) [26] to assist the development of bupropion dosing guidelines and to support DDI studies in drug discovery and development.

## Figures and Tables

**Table 1:** Drug-dependent parameters of the bupropion PBPK model.

Parameter	Value	Unit	Source	Literature	Reference	Description
MW	239.74	g/mol	lit.	239.74	[69]	Molecular Weight
pKa	8.75	-	lit.	8.75	[70]	Acid dissociation constant
Solubility (pH = 7.40)	365.56	mg/mL	lit.	365.56	[71]	Solubility
log P	2.57	-	fit.	3.27	[69]	Lipophilicity
fu	16.00	%	lit.	16.00	[23]	Fraction unbound
Intestinal perm.	2.76 × 10^−5^	cm/min	fit.	-	-	Transcellular intestinal permeability
Partition coefficients	Diverse	-	calc.	Berez.	[72]	Cell to plasma partitioning
Cellular Perm.	-	-	fit.	PK-Sim	[73]	Permeability into the cellular space
GFR fraction	1.00	-	asm.	-		Filtered drug in urine
EHC cont. fraction	1.00	-	asm.	-		Bile fraction continuously released
K_M_ CYP2B6*1 → HBup	^‡^ 25.80	µmol/L	lit.	^‡^ 25.80	[74]	Michaelis-Menten constant
k_cat_ CYP2B6*1 → HBup	* 10.87	1/min	fit.	-	-	Catalytic rate constant
K_M_ CYP2B6*6 → HBup	^‡^ 61.26	µmol/L	lit.	^‡^ 61.26	[74]	Michaelis-Menten constant
k_cat_ CYP2B6*6 → HBup	* 9.52	1/min	fit.	-	-	Catalytic rate constant
K_M_ CYP2B6*4 → HBup	12.70	µmol/L	lit.	12.70	[75]	Michaelis-Menten constant
k_cat_ CYP2B6*4 → HBup	^a^ 18.13	1/min	lit.	* 18.13	[75]	Catalytic rate constant
K_M_ 11β-HSD → EBup	39.10	µmol/L	lit.	39.10	[76]	Michaelis-Menten constant
k_cat_ 11β-HSD → EBup	2.15	1/min	fit.	-	-	Catalytic rate constant
K_M_ 11β-HSD → TBup	39.10	µmol/L	lit.	39.10	[76]	Michaelis-Menten constant
k_cat_ 11β-HSD → TBup	8.18	1/min	fit.	-	-	Catalytic rate constant
K_M_ CYP2C19	8.30	µmol/L	lit.	8.30	[77]	Michaelis-Menten constant
k_cat_ CYP2C19	2.59	1/min	fit.	-	-	Catalytic rate constant
K_D_ Binding partner	0.44	µmol/L	fit.	^b^ 0.35–0.60	[78,79,80]	Dissociation constant for binding
k_off_ Binding partner	0.05	1/min	fit.	-	-	Dissociation rate constant for binding

^‡^ in vitro values corrected for binding in the assay using fraction unbound to microsomal protein measurements from the same study, * half of the optimized parameter, ^a^ calculated mean of enantiomer selective degradation, ^b^ also includes inhibition constant values (Ki), 11β-HSD: 11β-hydroxysteroid-dehydrogenase, asm.: assumed, Berez.: Berezhkovskiy calculation method, calc.: calculated, cont.: continuous, CYP2B6: cytochrome P450 2B6, CYP2C19: cytochrome P450 2C19, EBup: erythrohydrobupropion, EHC: enterohepatic circulation, fit.: optimized parameter, GFR: glomerular filtration rate, HBup: hydroxybupropion, lit.: literature, perm.: permeability, PK-Sim: PK-Sim^®^ standard calculation method, TBup: threohydrobupropion.

**Table 2:** Drug-dependent parameters of the hydroxybupropion, erythrohydrobupropion and threohydrobupropion PBPK models.

Parameter	Value	Unit	Source	Literature	Reference	Value	Unit	Source	Literature	Reference	Description
	Hydroxybupropion					Erythro-and Threohydrobupropion					
MW	255.74	g/mol	lit.	255.74	[81]	241.76	g/mol	lit.	241.76	[82]	Molecular Weight
pKa	7.65	-	lit.	7.65	[81]	9.71	-	lit.	9.71	[82]	Acid dissociation constant
Solubility (pH = 7.40)	0.91	mg/mL	lit.	0.91	[81]	82.98	mg/mL	lit.	82.98	[82]	Solubility
log P	1.90	-	fit.	2.20	[83]	1.89	-	fit.	2.98	[82]	Lipophilicity
fu	23.00	%	lit.	23.00	[23]	58.00	%	lit.	58.00	[23]	Fraction unbound
Partition coefficients	Diverse	-	calc.	Berez.	[72]	Diverse	-	calc.	Berez.	[72]	Cell to plasma partitioning
Cellular Perm.	-	-	fit.	Ch.d.S.	[84]	-	-	fit.	Ch.d.S.	[84]	Permeability into the cellular space
GFR fraction	1.00	-	asm.	-	-	1.00	-	asm.	-	-	Filtered drug in urine
EHC cont. fraction	1.00	-	asm.	-	-	1.00	-	asm.	-	-	Bile fraction continuously released
K_M_ UGT2B7	^‡^ 14.64	µmol/L	lit.	14.64	[15]	(E) ^‡^ 9.33 (T) ^‡^ 6.22	µmol/L	lit.	(E) ^‡^ 9.33 (T) ^‡^ 6.22	[15]	Michaelis-Menten constant
k_cat_ UGT2B7	1.09	1/min	fit.	-	-	(E) 0.63(T) 0.10	1/min	fit.	-	-	Catalytic rate constant

^‡^ in vitro values corrected for binding in the assay using fraction unbound to microsomal protein measurements from the same study, asm.: assumed, Berez.: Berezhkovskiy calculation method, calc.: calculated, Ch.d.S.: Charge dependent Schmitt calculation method, cont.: continuous, E: erythrohydrobupropion, EHC: enterohepatic circulation, GFR: glomerular filtration rate, fit.: optimized parameter, lit.: literature, perm.: permeability, T: threohydrobupropion, UGT2B7: uridine 5′-diphospho-glucuronosyltransferase 2B7.

**Table 3:** Summary of quantitative measures of model performance for bupropion and its metabolites, separated by training and test dataset.

	Mean MRD		Mean GMFE_AUC_		Mean GMFE_Cmax_	
	training	test	training	test	training	test
Bupropion	1.62	1.90	1.20	1.42	1.20	1.41
Hydroxybupropion	1.16	1.30	1.14	1.34	1.10	1.32
Erythrohydrobupropion	1.48	1.38	1.25	1.46	1.26	1.38
Threohydrobupropion	1.36	1.21	1.36	1.23	1.17	1.30
Overall	1.51		1.31		1.29	
Profiles with measure ≤ 2	103/124		119/124		121/124	
Range	1.01–6.21		0.43–3.06		0.55–2.87	

AUC: area under the plasma concentration-time curve from the time of drug administration to the time of the last concentration measurement, C_max_: maximum plasma concentration, GMFE: geometric mean fold error, MRD: mean relative deviation.

## Data Availability

All modeling files including utilized clinical study data can be found at https://github.com/Open-Systems-Pharmacology (accessed on 31 December 2020).

## Supplementary Materials

The following are available at https://www.mdpi.com/1999-4923/13/3/331/s1, Electronic Supplementary Materials: A comprehensive reference manual, providing documentation of the complete model performance assessment.
