# Supplementary material for: Physiologically Based Pharmacokinetic Modeling of Bupropion and Its Metabolites in a CYP2B6 Drug-Drug-Gene Interaction Network
Source: Pharmaceutics. 2021 Mar 4;13(3):331. doi: 10.3390/pharmaceutics13030331 (PMC8001859; doi:10.3390/pharmaceutics13030331)
Supplement: Supplementary file 1 [file pharmaceutics-13-00331-s001.pdf]

Pharmaceutics

Physiologically Based Pharmacokinetic Modeling of Bupropion and its Metabolites in a CYP2B6 Drug-Drug-Gene Interaction Network

Supplementary Materials

Fatima Zahra Marok[1], Laura Maria Fuhr[1], Nina Hanke[1], Dominik Selzer[1], and Thorsten Lehr[1]

[1]Clinical Pharmacy, Saarland University, Saarbrücken, Germany

Funding:
The project has received support from the project "Open-source modeling framework for automated quality control and management of complex life science systems models" (OSMOSES), which is funded by the German Federal Ministry of Education and Research (BMBF, grant ID: 031L0161C). We acknowledge support by the Deutsche Forschungsgemeinschaft (DFG, German Research Foundation) and Saarland University within the funding programe "Open Access Publishing".

Conflict of Interest:
Thorsten Lehr has received research grants from the German Federal Ministry of Education and Research (grant 031L0161C). Fatima Zahra Marok, Laura Maria Fuhr, Nina Hanke and Dominik Selzer declare no conflict of interest. The funders had no role in the design of the study; in the collection, analyses, or interpretation of data; in the writing of the manuscript, or in the decision to publish the results.

Corresponding Author:
Prof. Dr. Thorsten Lehr
Clinical Pharmacy, Saarland University
Campus C2 2, 66123 Saarbrücken, Germany
Phone: +49 681 302 70255
Email: thorsten.lehr@mx.uni-saarland.de

Contents

1 PBPK modeling **4**
- 1.1 PBPK model building 4
- 1.2 Quantitative PBPK model evaluation 6
- 1.3 Sensitivity analysis 6
- 1.4 System-dependent parameters 8
- 1.5 Implementation - Interaction modeling 10
  - 1.5.1 Drug-gene-interaction 10
  - 1.5.2 Drug-drug-interaction 10
  - 1.5.3 Drug-drug-gene-interaction 11
- 1.6 Evaluation - Interaction modeling 12
  - 1.6.1 Drug-gene-interaction 12
  - 1.6.2 Drug-drug-interaction 12
  - 1.6.3 Drug-drug-gene-interaction 13

2 Bupropion model development **14**
- 2.1 Background 14
- 2.2 Clinical studies 16
- 2.3 Drug-dependent model parameters 18
- 2.4 Concentration-time profiles 20
- 2.5 Model evaluation 34
  - 2.5.1 Predicted compared to observed concentrations goodness-of-fit plots 34
  - 2.5.2 Mean relative deviation of plasma concentration predictions 35
  - 2.5.3 AUC and C_{max} goodness-of-fit plots 37
  - 2.5.4 Geometric mean fold error of predicted AUC and C_{max} values 38
  - 2.5.5 Local sensitivity analysis 42

3 DGI prediction **46**
- 3.1 Background 46
- 3.2 Clinical studies 47
- 3.3 Drug-dependent model parameters 48
- 3.4 Concentration-time profiles 49
- 3.5 Model evaluation 51
  - 3.5.1 Predicted compared to observed concentrations goodness-of-fit plots 51
  - 3.5.2 Mean relative deviation of plasma concentration predictions 52
  - 3.5.3 AUC and C_{max} goodness-of-fit plots 53
  - 3.5.4 Geometric mean fold error of predicted AUC and C_{max} values, $AUC_{HBup/Bup}$ and $C_{max, HBup/Bup}$ ratios, and DGI $AUC_{HBup/Bup}$ and DGI $C_{max, HBup/Bup}$ ratios 54

4 DDI prediction **57**
- 4.1 PBPK modeling of rifampicin 57
- 4.2 PBPK modeling of fluvoxamine 58
- 4.3 PBPK modeling of voriconazole 59
- 4.4 Clinical studies 60
- 4.5 Concentration-time profiles 61

4.6 Model evaluation 63
4.6.1 Predicted compared to observed concentrations goodness-of-fit plots 63
4.6.2 Mean relative deviation of plasma concentration predictions 64
4.6.3 AUC and C_{max} goodness-of-fit plots 65
4.6.4 Geometric mean fold error of predicted AUC and C_{max} values, $AUC_{HBup/Bup}$ and $C_{max, HBup/Bup}$ ratios, and DDI $AUC_{HBup/Bup}$ and DDI $C_{max, HBup/Bup}$ ratios 66

5 DDGI prediction **69**
5.1 Background 69
5.2 Clinical studies 69
5.3 Concentration-time profiles 70
5.4 Model evaluation 71
5.4.1 DDGI $AUC_{HBup/Bup}$ ratios goodness-of-fit plots 71
5.4.2 Geometric mean fold error of predicted DDGI $AUC_{HBup/Bup}$ ratios 72
5.4.3 DDGI scenarios of rifampicin-bupropion interactions 73

References **74**

1 PBPK modeling

1.1 PBPK model building

A parent-metabolite PBPK model for bupropion and its metabolites hydroxybupropion, erythrohydrobupropion and threohydrobupropion was developed. The metabolic pathways and interactions implemented to describe their pharmacokinetics in the CYP2B6 network are illustrated in Figure S1.1.1. Physiological parameters, such as tissue volumes or surface areas, are predefined in the PBPK modeling software PK-Sim® (Version 9.1) [1]. Further system-dependent parameters such as reference concentrations (concentration in the tissue with the highest expression) and tissue expression profiles of metabolizing enzymes and transporters, are listed in Table S1.1. Demographic data were derived from the collected clinical study reports and are listed in the study tables of the respective sections. The drug-dependent parameters of the developed bupropion parent-metabolite PBPK model are listed in Section 2.

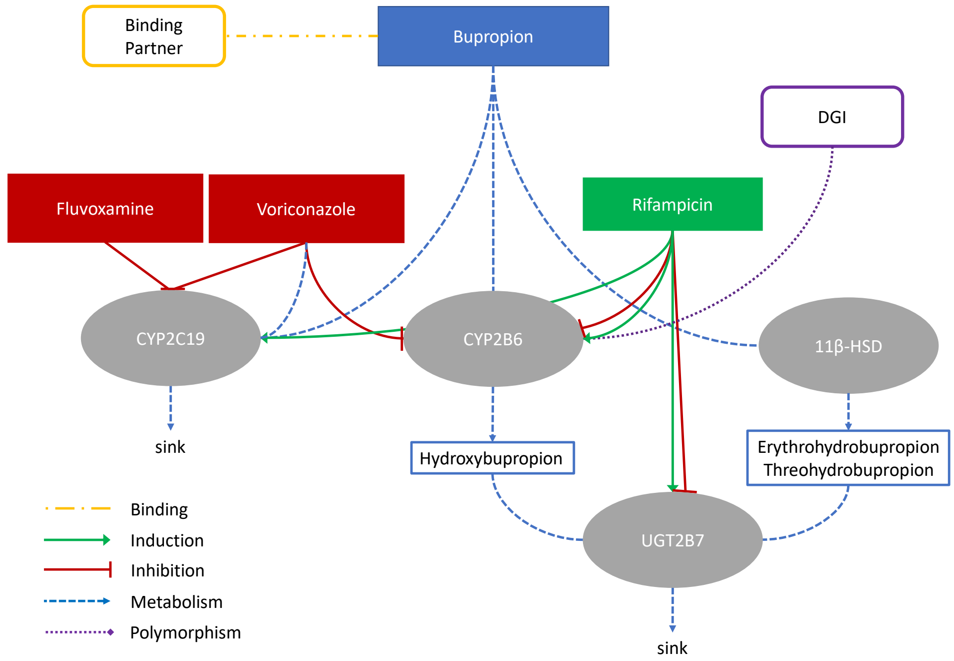

Figure S1.1.1: **Metabolic pathways and interactions implemented in the CYP2B6 network.** Bupropion is metabolized via CYP2B6 to hydroxybupropion and via 11β-HSD to erythrohydrobupropion and threohydrobupropion. Additionally, CYP2C19-mediated metabolism was included. Since bupropion binds to different therapeutic targets, binding to an unspecific protein (Binding Partner) was implemented as well. The metabolites are further degraded by UGT2B7. Drug-gene-interactions (DGIs), drug-drug-interactions (DDIs) as well as drug-drug-gene-interactions (DDGIs) were simulated for CYP2B6 with the perpetrators rifampicin, fluvoxamine and voriconazole. **11β-HSD**, 11β-hydroxysteroid dehydrogenase; **CYP**, cytochrome P450; **DGI**, drug-gene-interaction; **UGT**, uridine 5'-diphosphoglucuronosyltransferase.

Bupropion formulations

For simulation of oral tablets with different bupopion release, the weibull function was used according to Equation S1 [73], to describe immediate, sustained, and extended release formulations, as well as the cocktail capsule formulation (Geneva cocktail [74]) used in the Bosilkovska et al. 2014 and 2016 studies [24, 74].

Weibull model

$$m = 1 - exp\left(\frac{-(t - T_{lag}^{b})}{a}\right) \text{ with } a = (T_d)^b \tag{S1}$$

a = scale parameter
b = shape paramter
m = fraction of the dissolved drug at time t
T_d = time needed to dissolve 63% of the formulation
T_{lag} = lag time before the onset of dissolution

The parameters used in the presented model are listed in the drug-dependent parameter table (Table S2.2).

1.2 Quantitative PBPK model evaluation

The model performance was evaluated by comparing predicted plasma concentration-time profiles to observed data which are displayed in the following sections in linear and semilogarithmic scale (Figures S2.4.2-S2.4.15) and in goodness-of-fit plots (Figure S2.5.16). Furthermore, the models were evaluated by comparing predicted to observed area under the plasma concentration-time curve (AUC) and maximum plasma concentration (C_{max}) values (Figures S2.5.17-S2.5.18). Figures S2.5.19-S2.5.22 illustrate results of local sensitivity analyses as bar graphs.
As quantitative performance measures, the mean relative deviation (MRD) was calculated for all profiles from their respective predicted and observed plasma concentrations (Equation (S2)). Furthermore, the geometric mean fold errors (GMFE) of the AUC_{last} (AUC from the first time point to the last time point of concentration measurement of drug administration) and C_{max} were calculated according to Equation (S3).

Equation: Mean relative deviation

$$MRD = 10^x \text{ with } x = \sqrt{\frac{1}{n}\sum_{i=1}^{n}(\log_{10}\hat{c}_i - \log_{10}c_i)^2} \qquad \text{(S2)}$$

c_i = the ith observed plasma concentration
$\hat{c}_i$ = the respective predicted plasma concentration
n = number of observed values

Overall MRD values of ≤ 2 were considered reasonable predictions.
The GMFE was calculated for all AUC_{last} and C_{max} values according to Equation (S3).

Equation: Geometric mean fold error

$$GMFE = 10^x \text{ with } x = \frac{1}{n}\sum_{i=1}^{n}|\log_{10}(\frac{\hat{a}_i}{a_i})| \qquad \text{(S3)}$$

a_i = the ith observed AUC_{last} or C_{max} value
$\hat{a}_i$ = the respective predicted AUC_{last} or C_{max} value
n = number of studies

Overall GMFE values of ≤ 2 were considered reasonable predictions.

1.3 Sensitivity analysis

Sensitivity of the final models to single parameter changes (local sensitivity analysis) was calculated as relative change of the AUC_{last}. It was carried out using a relative perturbation of 1000% (variation range 10.0, maximum number of 9 steps). Parameters were included into the analysis if they were optimized or assumed to have an impact on AUC. Sensitivity to a parameter was calculated as the ratio of relative

change of the simulated AUC_{last} to the relative variation of the parameter value used in the final model according to Equation (S4).

Equation: Sensitivity analysis

$$S = \frac{\Delta AUC_{last}}{\Delta p} * \frac{p}{AUC_{last}} \tag{S4}$$

ΔAUC = change of the AUC_{last}
AUC = simulated AUC_{last} with the original parameter value
Δp = change of the examined parameter value
p = original parameter value
S = sensitivity of the AUC_{last} to the examined model parameter

A sensitivity of + 1.0 signifies that a 10% increase of the examined parameter value causes a 10% increase of the simulated AUC_{last}.

1.4 System-dependent parameters

System-dependent parameters, such as reference concentrations and tissue expression profiles of metabolizing enzymes and transporters, are listed in Table S1.1.

Table S1.1: System-dependent parameters.

Protein (*Gene*)	Reference concentration		Relative expression [a]	Localization	Half-life [h]	
	Mean[b] [µmol/l]	GeoSD[c]			Liver	Intestine
CYP2B6 (*CYP2B6*)	1.56	[d]1.40	RT-PCR [2]	intracellular	32	23
CYP2C19 (*CYP2C19*)	0.76	1.79 [16]	RT-PCR [2]	intracellular	26	23
11β-HSD (*HSD11B1*)	[e]1.0	[d]1.40	Array [3]	intracellular	36	23
UGT2B7 (*UGT2B7*)	[f]0.28 [4]	1.56 [16]	EST [5]	intracellular	36	23
NRT[g] (*SLC6A2*)	[e]1.0	[d]1.40	EST [5]	[h]membrane	36	23

11β-HSD, 11β-hydroxysteroid dehydrogenase 1; **conc.**, concentration **CYP**, cytochrome P450; **Array**, microarray expression profile; **EST**, expressed sequence tag expression profiles from UniGene; **NRT**, noradrenaline reuptake transporter; **RT-PCR**, reverse transcription-polymerase chain reaction measured expression profile; **UGT**, uridine 5'-diphospho-glucuronosyltransferase.

[a], in the different organs (PK-Sim® expression database profile)

[b], µmol protein/l in the tissue of highest expression

[c], geometric standard deviation of the reference concentration

[d], if no information was available, a moderate variability of 35% CV was assumed (1.40 GeoSD)

[e], no information was available, thus, the mean reference concentration was set to 1.0 µmol/l and the catalytic rate constant (k^{cat}) was optimized according to Meyer et al. 2012 [6].

[f], calculated from transporter per mg membrane protein times 26.2 mg human kidney microsomal protein per g kidney tissue [7]

[g], expression profile used for general binding partner

[h], extracellular membrane

Virtual individual

Proteins were implemented for every modeled individual. The individuals were created based on the demographics mentioned in the respective clinical study report. If no data was available a standard individual was used. This standard individual, similar to the Standard European Male implemented in OSP®, is based on the demographic databases used in the modeling software. Additionally, every individual had an activated enterohepatic circulation (EHC continuous fraction = 1). The characteristics of the standard individuals are compared in Table S1.2.

Virtual population

Virtual population were created based on 500 individuals, with their demographic information (age range, sex composition, ethnicity) derived from the respective clinical study report. If no information on ethnicity and sex composition was given, a 100% European male population with and age range of 20-50 years was assumed. System-dependent parameters, e.g. weight, height, organ volumes or blood flow rates, were varied by the implementd algorithm in PK-Sim® based on the limits of the following databases: American: Third National Health and Nutrition Examination Survey (NHANES)[15] database, Asian: Tanaka model [9], European: International Commission on Radiological Protection (ICRP) database [8]. The reference concentrations of the metaolizing enzymes and transporters as listed in Table S1.1, were log-normaly distributedmaccording to the variabilty reported in the ontogeny database implemented in PK-Sim® [16]. If no information could be found, reference concentrations were distributed with a variability of 35% CV (geometric standard deviation of 1.4).

Table S1.2: Standard individual demographics.

Individual	Age [years]	Weight [kg]	Height [cm]	BSA [m²]	BMI [kg/m²]	Reference
OSP® - Standard European Male[a]	30	73	176	1.89	23.57	[8]
European Female[b]	30	60	163	1.65	22.58	[8]
Asian Male[c]	30	60	170	1.68	20.78	[9]

BMI, body mass index; **BSA**, body surface area; **OSP®**, open systems pharmacology®.
[a], standard individual implemented in the modeling software, used for every study where demographical data was missing
[b], only used for Palovaara 2003 [10]
[c], only used for Fan 2009 [11], Gao 2012 [12], Gao 2016 [13] and Qin 2012 [14], since asian populations was assumed.

1.5 Implementation - Interaction modeling

1.5.1 Drug-gene-interaction

In order to describe the effect of different *CYP2B6* genotypes on the compounds' PK, the enzyme activity was implemented as two enzymes for each allele. The single alleles were modeled with Michaelis-Menten constant (K_M) values from literature (after correction for microsomal binding). For two genetic variants (*CYP2B6*4* and *CYP2B6*5*), the catalytic rate constant, k_{cat} was calculated from reported maximum velocity (V_{max}) in vitro measurements. For *CYP2B6*1* and *CYP2B6*6*, k_{cat} was optimized with reported plasma concentration-time profiles of populations with homozygous expression of the respective haplotype. All DGI parameters are shown in Table S3.2 in Section 3.1.

1.5.2 Drug-drug-interaction

DDIs were simulated for bupropion as the victim drug. For the CYP2B6 interaction network, the investigated perpetrators included rifampicin as inducer and fluvoxamine and voriconazole as inhibitors. Interaction parameters were informed from the literature and are listed in the respective parameter tables (Table S4.1,S4.2 and S4.3).

Mathematical implementation of induction

Drug-induced increase of gene expression and therefore, enzyme activity, was calculated as shown in Equations (S5) and (S6).

Implementation of enzyme induction

$$\frac{d[E]}{dt} = R_{syn,app} - k_{deg} * [E] \tag{S5}$$

$$R_{syn,app} = R_{syn} * (1 + \frac{E_{max} * [I]}{EC_{50} + [I]}) \tag{S6}$$

$\frac{d[E]}{dt}$ = enzyme turnover rate
$[E]$ = enzyme concentration
EC_{50} = inducer concentration to reach half-maximal induction in vivo
E_{max} = maximum induction effect in vivo
$[I]$ = free inducer concentration
k_{deg} = degradation rate constant
R_{syn} = enzyme synthesis rate in absence of inducer
$R_{syn,app}$ = enzyme synthesis rate in presence of inducer

Mathematical implementation of inhibition

Inhibition of enzyme activites was implemented as a competitive inhibition process. Competitive inhibition occurs if the inhibitor binds reversibly to the active site of an enzyme and hence, competes with

the substrate over the binding spot. Since the inhibitor binds reversibly to the enzyme, high substrate concentrations can overcome its inhibition. The process is calculated as shown in Equations (S7) and (S8).

Implementation of enzyme inhibition

$$v = \frac{V_{max} * [S]}{K_{M,app} + [S]} \tag{S7}$$

$$K_{M,app} = K_M * (1 + \frac{[I]}{K_I}) \tag{S8}$$

I = free inhibitor concentration
K_I = dissociation constant of the inhibitor-enzyme complex
K_M = Michaelis-Menten constant in absence of inhibitor
$K_{M,app}$ = apparent Michaelis-Menten constant in presence of inhibitor
S = free substrate concentration
v = reaction velocity
V_{max} = maximum reaction velocity

1.5.3 Drug-drug-gene-interaction

Drug-drug-gene-interactions (DDGIs) were simulated for various genotypes after concomitant rifampicin intake. The underlying effects on the PK were implemented according to the Sections 1.5.1 and 1.5.2.

1.6 Evaluation - Interaction modeling

1.6.1 Drug-gene-interaction

In addition to Section 1.2, the effect of DGIs was evaluated by calculation of the ratio of hydroxybupropion to bupropion AUC_{last} and C_{max} values in plasma as shown in Equation (S9).The calculated ratios are illustrated in Figure S3.5.4.

DGI effect ratio

$$PK_{HBup/Bup} = \frac{PK(HBup)}{PK(Bup)} \tag{S9}$$

Bup = PK parameter of bupropion
$HBup$ = PK parameter of hydroxybupropion
$PK_{HBup/Bup}$ = HBup/Bup ratio of the PK parameter
PK = PK parameter such as AUC_{last} or C_{max}

Additionally, DGI effect ratios for the ratio of hydroxybupropion to bupropion AUC_{last} and C_{max} values were calculated according to Equation (S10) for predicted and observed concentrations. The calculated ratios are illustrated in Figure S3.5.4.

DGI effect hydroxybupropion/bupropion ratio

$$DGI\ PK_{HBup/Bup} = \frac{PK_{HBup/Bup}(Effect)}{PK_{HBup/Bup}(Control)} \text{ with } PK_{HBup/Bup} = \frac{PK_{HBup}}{PK_{Bup}} \tag{S10}$$

Bup = bupropion
$HBup$ = hydroxybupropion
$PK_{HBup/Bup}(Control)$ = HBup/Bup ratio of the PK parameter of wildtype CYP2B6
$PK_{HBup/Bup}(Effect)$ = HBup/Bup ratio of the PK parameter of a variant CYP2B6 genotype
PK = PK parameter such as AUC_{last} or C_{max}

1.6.2 Drug-drug-interaction

Similar to the DGIs, the effect of DDIs was evaluated by calculation of the ratio of hydroxybupropion to bupropion AUC_{last} and C_{max} values according to Equation (S9). The calculated ratios are illustrated in Figure S4.6.4. Additionally, DDI effect ratios for the ratio of hydroxybupropion to bupropion AUC_{last} and C_{max} were calculated as shown in Equation (S11) for predicted and observed concentrations. The calculated DDI ratios are illustrated in Figure S4.6.4.

DDI effect hydroxybupropion/bupropion ratio

$$DDI\ PK_{HBup/Bup} = \frac{PK_{HBup/Bup}(Effect)}{PK_{HBup/Bup}(Control)} \text{ with } PK_{HBup/Bup} = \frac{PK_{HBup}}{PK_{Bup}} \qquad \text{(S11)}$$

Bup = bupropion
$HBup$ = hydroxybupropion
$PK_{HBup/Bup}(Control)$ = HBup/Bup ratio of the PK parameter without perpetrator
$PK_{HBup/Bup}(Effect)$ = HBup/Bup ratio of the PK parameter with perpetrator
PK = PK parameter such as AUC_{last} or C_{max}

1.6.3 Drug-drug-gene-interaction

The majority of compiled DDGI data only included AUC_{inf} (AUC extrapolated to infinity) ratios of hydroxybupropion and bupropion, with only one study showing plasma concentration-time profile. Hence, the effect of DDGIs was evaluated by calculation of DDGI effect ratios for $AUC_{HBup/Bup}$ (AUC_{last} or AUC_{inf}) in plasma as shown in Equation (S12). The calculated DDGI effect ratios for the ratio of hydroxybupropion to bupropion are illustrated in Figure S5.4.2.

DDGI effect hydroxybupropion/bupropion ratio

$$DDGI\ AUC_{inf,HBup/Bup} = \frac{AUC_{inf,HBup/Bup}(Effect)}{AUC_{inf,HBup/Bup}(Control)} \text{ with } AUC_{inf,HBup/Bup} = \frac{AUC_{inf,HBup}}{AUC_{inf,Bup}} \qquad \text{(S12)}$$

Bup = bupropion
$HBup$ = hydroxybupropion
$AUC_{inf,HBup/Bup}(Control)$ = HBup/Bup ratio of AUC_{inf} of wildtype CYP2B6 without perpetrator
$AUC_{inf,HBup/Bup}(Effect)$ = HBup/Bup ratio of AUC_{inf} of a variant CYP2B6 genotype with perpetrator

2 Bupropion model development

2.1 Background

Bupropion is a noradrenaline and dopamine reuptake inhibitor used in the treatment of major depressive disorder and to aid smoking cessation [17]. In therapy, the compound is either administered as monotherapy or in combination with additional anti-depressant agents [17, 18]. Bupropion is pharmacologically active, but is also transformed to three active metabolites [19].
One metabolite, hydroxybupropion, is formed by CYP2B6 mediated hydroxylation of bupropion. Erythro- and threohydrobupropion are formed through several metabolic steps of which reduction by carbonyl reductase 11β-HSD metabolizes is the rate-limiting step [20]. To some extent, further CYP enzymes, i.e. CYP2C19, are also involved in bupropion degradation [21]. Therefore, the presented model includes transformation via CYP2B6, 11β-HSD and CYP2C19. The three metabolites are subsequently glucuronidated via UGT2B7, which is also implemented in the model.
Bupropion binds and inhibits reuptake transporters for noradrenaline, dopamine and acetylcholine [22, 23]. This target-mediated binding was modeled by implementation of a surrogate binding parther, representing various different targets. An expression profile of the noradrenaline reuptake transpoter 1 was used for the surrogate binding partner.
Data from 48 clinical studies were used for model development and split into a training dataset, used for model building and parameter optimization, and a test dataset, used for model evaluation. Here, bupropion (20 mg to 450 mg) was administered as oral formulations with different release kinetics (Table S2.1). Drug-dependent parameters for the parent-metabolite model featuring bupropion, hydroxybupropion, erythorhydrobupropion and threohydrobupropion are listed in Table S2.2.
Several model input parameters that could not be informed from the literature, were optimized, including k_{cat} values for all metabolic reactions. To model the target-binding of bupropion, binding to various pharmacological targets was summarized by including one target protein as a binding partner.
Figure S2.1.1 illustrates a quantitative mass-balance diagram of the elimination pathways of bupropion. Bupropion is predicted to be absorbed completely. Metabolism via CYP2B6 accounts for 58 %, 11β-HSD for 28% and CYP2C19 for 13% of total bupropion. In urine, 1% of unchanged bupropion is predicted to be excreted, while almost no bupropion can be simulated in faeces. Influence of the first pass metabolism could not be determined, as data of intravenous administration of bupropion were not available and therefore, not evaluated.
The good performance of the model is demonstrated in linear (Fig. S2.4.2, S2.4.3, S2.4.8, S2.4.9 and S2.4.14) and semilogarithmic plots (Fig. S2.4.5, S2.4.6, S2.4.11, S2.4.12 and S2.4.15) of predicted compared to observed plasma concentration-time profiles of all clinical studies. Furthermore, goodness-of-fit plots comparing predicted to their corresponding observed plasma concentrations are presented (Fig. S2.5.16) and calculated MRD values for each study are listed in Table S2.4. Additionally, correlation plots of predicted versus observed AUC_{last} and C_{max} values are shown in Figures S2.5.17 and S2.5.18. A summary of the respective PK parameters, including calculated GMFE values, is shown in Table S2.4. Local sensitivity analysis results for simulations of 300 mg bupropion administered as immediate release (100 mg three times daily), sustained release (150 mg two times daily) or extended release (300 mg once daily) tablets are presented in Section 2.5.5.

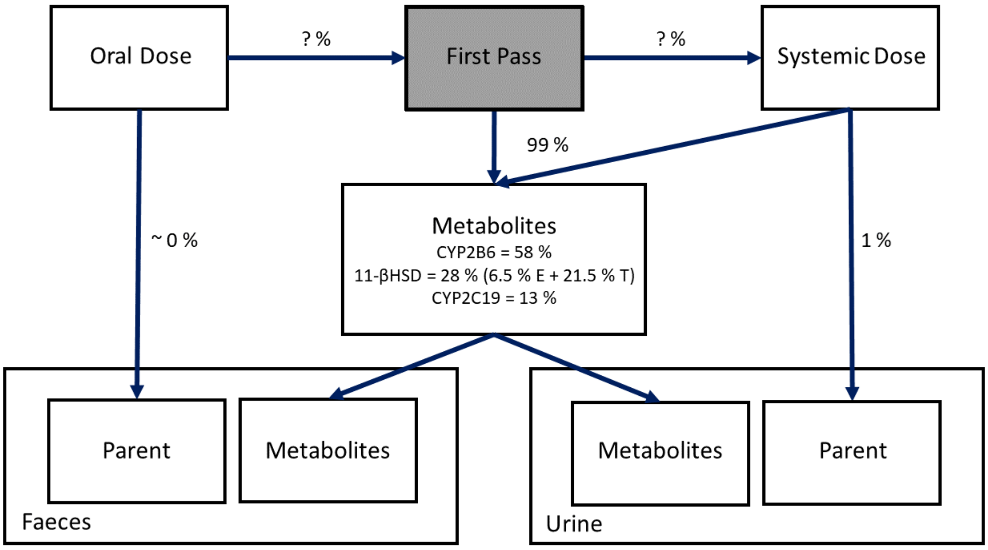

Figure S2.1.1: **Quantitative mass-balance diagram of the elimination pathways of bupropion.** Metabolism via CYP2B6, via 11β-HSD and CYP2C19 accounts for 99% of total bupropion. One % of bupropion is excreted unchanged to urine, while no bupropion is predicted to be in feces. **11β-HSD**, 11β-hydroxysteroid dehydrogenase; **CYP**, cytochrome P450; **E**, erythrohydrobupropion; **T**, threohydrobupropion.

2.2 Clinical studies

In Table S2.1, all clinical studies used for model development are listed. Virtual individuals were built according to the demographics published in the respective study reports. If no data on the demographics were reported, a standard individual was used as described in Section 1.4.

Table S2.1: Clinical studies used for bupropion model development.

Dosing	n	Age [years]	Weight [kg]	BMI [kg/m²]	Females [%]	CYP2B6 genotype (n)	Dataset	Reference
20 mg Cap (s.d.)	30	23.5 (18–36)	-	21.7 (18.4–27.7)	50	*1/*6 (16), *6/*6 (1)	ta	Bosilkovska 2016 [24]
25 mg Cap (s.d.)	10	23 (20–36)	-	22 (19.9–24.4)	0	*1/*6 (4), *6/*6 (2)	te	Bosilkovska 2014 [25]
50 mg IR (s.d.)	24	19–43	-	-	50	-	ta	Findlay 1981 [26]
75 mg IR (s.d.)	20	18–55	72.3 (53.6–88.9)	19.5–28.3	50	-	ta	Zahner 2014 [27]
75 mg IR (s.d.)	7	18–45	-	-	100	-	te	Hesse 2006 [28]
75 mg IR (s.d.)	33	25–55	67.5 (56.3–107)	18.5–35	51.5	-	te	Connarn 2017 [18]
100 mg IR (s.d.)	33	25–55	67.5 (56.3–107)	18.5–35	51.5	-	ta	Connarn 2017 [18]
100 mg IR (s.d.)	24	19–43	-	-	50	-	ta	Findlay 1981 [26]
100 mg IR (s.d.)	15	24 (19–47)	74.8	25	40	-	te	Masters 2016 [29]
100 mg IR (s.d.)	24	43.5	72.9	26.5	45.8	-	te	Yamazaki 2017 [30]
100 mg IR (s.d.)	8	20-35	-	-	0	-	te	Posner 1984 [31]
100 mg IR (s.d.)	8	29.5 (22–42)	80 (66–101)	-	-	-	te	Posner 1985 [32]
100 mg IR (m.d.)	8	29.5 (22–42)	80 (66–101)	-	-	-	te	Posner 1985 [32]
100 mg IR (m.d.)	30	-	-	-	-	-	ta	Patent 1a (US2006/0228415A1) [33]
150 mg IR (s.d.)	10	31 (21–40)	73 (57–84)	-	60	-	ta	Kharasch 2008 [34]
150 mg IR (s.d.)	13	29 (23–39)	67 (53–84)	-	-	-	te	Kharasch 2008b [35]
200 mg IR (s.d.)	24	19–43	-	-	50	-	ta	Findlay 1981 [26]
100 mg SR (s.d.)	33	25–55	67.5 (56.3–107)	18.5–35	51.5	-	te	Connarn 2017 [18]
100 mg SR (s.d.)	12	20–44	-	-	16.7	-	te	Hogeland 2007 [36]
150 mg SR (m.d.)	42	31.8 (19–64)	74.7 (56.2–105.4)	25.4 (18.7–39.5)	38	*1/*5 (2), *1/*6 (6), *5/*6 (3), *6/*6 (4)	te	Benowitz 2013 [37]
150 mg SR (s.d.)	22	22.7	65	-	27.3	*1/*4 (3), *1/*6 (11), *6/*6 (2)	ta	Chung 2011 [38]
150 mg SR (s.d.)	33	25–55	67.5 (56.3–107)	18.5–35	51.5	-	ta	Connarn 2017 [18]
150 mg SR (s.d.)	24	-	-	-	-	-	te	Dennison 2018 [39]
150 mg SR (s.d.)	17	-	-	-	-	*1/*6 (6), *6/*6 (5)	te	Fan 2009 [11]
150 mg SR (s.d.)	30	29 (18–53)	75 (56–96)	24 (20–30)	0	-	te	Farid 2008 [40]
150 mg SR (s.d.)	19	-	-	-	0	-	ta	Gao 2012 [12]
150 mg SR (s.d.)	34	26.2	66.5	-	47.1	-	te	Hsyu 1997 [41]
150 mg SR (s.d.)	14	21.3	61.3	-	0	-	te	Lei 2009 [42]
150 mg SR (s.d.)	18	21.3	62.7	-	0	-	te	Lei 2010 [43]
150 mg SR (s.d.)	18	22 (19–34)	72 (53–99)	23.1 (18.4–26.9)	0	*1/*4 (1), *1/*5 (1), *1/*6 (6), *5/*5 (1), *4/*6 (1), *6/*6 (1), *6/*14 (1)	ta	Loboz 2006 [44]
150 mg SR (s.d.)	12	20–25	50–75	18–27	100	-	ta	Palovaara 2003 [10]
150 mg SR (m.d.)	49	-	-	-	-	-	ta	Patent 1b (US2006/0228415A1) [33]
150 mg SR (m.d.)	7	-	-	-	-	-	te	Patent 2 (US8545880B2) [45]
150 mg SR (s.d.)	16	20–23	62–85	21–26	0	*1/*6 (6), *6/*6 (4)	te	Qin 2012 [14]
150 mg SR (s.d.)	13	39 (21–54)	86	-	23.1	-	te	Robertson 2008 [46]
150 mg SR (s.d.)	12	22–27	67–95	21–26	0	-	te	Turpeinen 2005 [47]

Table S2.1: Clinical studies used for bupropion model development. (continued)

**Dosing**	**n**	**Age [years]**	**Weight [kg]**	**BMI [kg/m²]**	**Females [%]**	***CYP2B6* genotype (n)**	**Dataset**	**Reference**
150 mg SR (s.d.)	17	27.3 (21–50)	73.9 (±8.9)	23.5 (±2.0)	41.7	-	te	Turpeinen 2007 [48]
150 mg SR (s.d.)	10	39.6 (32–43)	78.2 (±18.6)	26.4 (±5.3)	50	-	te	Turpeinen 2007 [48]
150 mg SR (s.d.)	16	61.9 (50–70)	-	-	100	-	te	Turpeinen 2013 [49]
300 mg SR (s.d.)	24	29 (18–45)	77 (56–96)	-	0	-	te	Kustra 1999 [50]
150 mg ER (s.d.)	33	25–55	67.5 (56.3–107)	18.5–35	51.5	-	ta	Connarn 2017 [18]
300 mg ER (s.d.)	33	25–55	67.5 (56.3–107)	18.5–35	51.5	-	te	Connarn 2017 [18]
300 mg ER (m.d.)	30	-	-	-	-	-	te	Patent 1a (US 2006/0228415 A1) [33]
300 mg ER (m.d.)	49	-	-	-	-	-	ta	Patent 1b (US 2006/0228415 A1) [33]
300 mg ER (m.d.)	38	-	-	-	-	-	te	Patent 3 (US7,645,802B2) [51]
300 mg ER (m.d.)	16	24.3	-	22.7	50	-	te	Schmid 2012 [52]
300 mg ER (m.d.)	-	-	-	-	-	-	ta	Woodcock 2012 [53]
450 mg ER (m.d.)	20	-	-	-	-	-	te	Paiement 2012 [54]

BMI, body mass index; **Cap**, capsule (Geneva cocktail [25]); **CYP**, cytochrome P450; **ER**, extended release formulation; **IR**, immediate release formulation; **m.d.**, multiple dose; **n**, number of individuals studied; **s.d.**, single dose; **SR**, sustained release formulation; **ta**, training dataset; **te**, test dataset; **-**, no data available. Values are given as mean ± standard deviation (SD), the range of values is given in brackets.

2.3 Drug-dependent model parameters

Table S2.2 lists the drug-dependent parameters used in model development of the final parent-metabolite model.

Table S2.2: Drug-dependent parameters of the bupropion PBPK model.

Parameter	Unit	Value	Source	Reference	Value	Source	Reference	Value	Source	Reference	Description
		Bupropion			Hydroxybupropion			Erythro-/Threohydrobupropion			
MW	g/mol	239.74	lit.	[55]	255.74	lit.	[56]	241.76	lit.	[57]	Molecular weight
pKa (base)	-	8.75	lit.	[58]	7.65	lit.	[56]	9.71	lit.	[57]	Acid dissociation constant
Solubility (pH)	mg/ml	364.56 (7.4)	lit.	[59]	0.91 (7.4)	lit.	[56]	82.98 (7.4)	lit.	[57]	Solubility
logP	-	[a]2.70	3.27	[55]	[a]1.90	2.20	[60]	[a]1.76	2.88	[61]	Lipophilicity
fu	%	16.00	lit.	[62]	23.00	lit.	[62]	58.00	lit.	[62]	Fraction unbound
Spec. int. perm.	cm/min	[a]3.30E-5	[b]2.42E-4	-	[b]2.78E-5	[b]2.78E-5	-	[b]2.64E-5	[b]2.64E-5	-	Normalized to surface area
Org. perm.	cm/min	[b]0.14	[b]0.14	-	[b]0.01	[b]0.01	-	[b]0.01	[b]0.01	-	Normalized to surface area
GFR frac.	-	1.00	asm.	-	1	asm.	-	1	asm.	-	Fraction of filtered drug in urine
EHC cont. frac.	-	1.00	asm.	-	1	asm.	-	1	asm.	-	Bile fraction continuously released
Cell. perm.	cm/min	Ch. d. S.	-	[63]	Ch. d. S.	-	[63]	Ch. d. S.	-	[63]	Permeation across cell membranes
Part. coef.	-	PK-Sim	-	[1]	Berez.	-	[64]	Berez.	-	[64]	Organ-plasma partition coefficients
11β-HSD K_M	µmol/l	39.10	lit.	[65]	-	-	-	-	-	-	11β-HSD Michaelis-Menten constant
11β-HSD kcat (EBup)	1/min	[a]2.15	-	-	-	-	-	-	-	-	11β-HSD catalytic rate constant for formation of EBup
11β-HSD kcat (TBup)	1/min	[a]8.18	-	-	-	-	-	-	-	-	11β-HSD catalytic rate constant for formation of TBup
CYP2B6 K_M	µmol/l	[c]25.80	lit.	[66]	-	-	-	-	-	-	CYP2B6 Michaelis-Menten constant
CYP2B6 kcat	1/min	[a]21.74	-	-	-	-	-	-	-	-	CYP2B6 catalytic rate constant for wildtype
CYP2C19 K_M	µmol/l	8.30	lit.	[67]	-	-	-	-	-	-	CYP2C19 Michaelis-Menten constant
CYP2C19 kcat	1/min	[a]2.59	-	-	-	-	-	-	-	-	CYP2C19 catalytic rate constant
BP K_D	µmol/l	[a]0.44	[d] 0.35, 0.53, 0.87, 6.98	[68–70]	-	-	-	-	-	-	Dissociation constant for binding
BP koff	1/min	[a]0.05	-	-	-	-	-	-	-	-	Dissociation rate constant for binding
UGT2B7 K_M	µmol/l	-	-	-	[c]14.64	lit.	[71]	[c]9.33 (EBup), [c]6.22 (TBup)	lit.	[71]	UGT2B7 Michaelis-Menten constant
UGT2B7 kcat	1/min	-	-	-	[a]1.38	-	-	[a]0.38 (EBup), [a]0.10 (TBup)	-	-	UGT2B7 catalytic rate constant
Weibull shape	-	[a]4.43 (Cap)	-	-	-	-	-	-	-	-	Shape used for Weibull
	-	[a]0.75 (IR)	-	-	-	-	-	-	-	-	
	-	[a]1.00 (SR)	-	-	-	-	-	-	-	-	
	-	[a,f]0.60 (SR)	-	-	-	-	-	-	-	-	

Table S2.2: Drug-dependent parameters of the bupropion PBPK model. (continued)

Parameter	Unit	Value	Source	Reference	Value	Source	Reference	Value	Source	Reference	Description
	-	^{e}1.88 (ER)	^{e}1.88	[33]	-	-	-	-	-	-	
Weibull time	min	^{a}10.64 (Cap)	-	-	-	-	-	-	-	-	Time of 50% dissolved
	min	^{a}3.12 (IR)	-	-	-	-	-	-	-	-	
	min	^{a}100.00 (SR)	-	-	-	-	-	-	-	-	
	min	^{a,f}54.13 (SR)	-	-	-	-	-	-	-	-	
	min	^{e}230.00 (ER)	^{e}230.00	[33]	-	-	-	-	-	-	

11β-HSD, 11β-hydroxysteroid dehydrogenase 1; **asm.**, assumed; **Berez.**, Berezhkovskiy calculation method; **BP**, binding partner; **Cap**, capsule (Geneva cocktail [25]); **cell. perm.**, cellular permeabilities; **Ch. d. S.**, Charge dependent Schmitt calculation method; **CYP**, cytochrome P450; **EBup**, erythrohydrobupropion; **EHC**, enterohepatic circulation; **ER**, extended release formulation; **frac.**, fraction; **IR**, immediate release formulation; **GFR**, glomerular filtration rate; **lit.**, literature; **org. perm.**, organ permeability; **part. coeff.**, partition coefficients; **PK-Sim**, PK-Sim Standard calculation method; **spec. int. perm.**, specific intestinal permeability; **SR**, sustained release formulation; **TBup**, threohydrobupropion; **UGT**, uridine 5'-diphospho-glucuronosyltransferase; **-**, not available.

^{a}, optimized

^{b}, calculated parameter

^{c}, in vitro values corrected for binding in the assay using fraction unbound to microsomal protein measurements from the same study [72]

^{d}, range also includes inhibition constant values (K_i)

^{e}, calculated dissolution parameter after Langenbuchener et al. 1972 [73]

^{f}, used for Fan 2009 [11], Gao 2012 [12], Gao 2016 [13], Lei 2009 [42], Lei 2010 [43] and Qin 2012 [14]

2.4 Concentration-time profiles

The geometric means of the population predictions (n=500) are shown as solid lines and corresponding observed data as filled dots. Symbols represent the arithmetic mean values ± standard deviation, if available. The shaded areas indicate the geometric standard deviation. Details on dosing regimens, study populations and literature references are listed in Table S2.1.

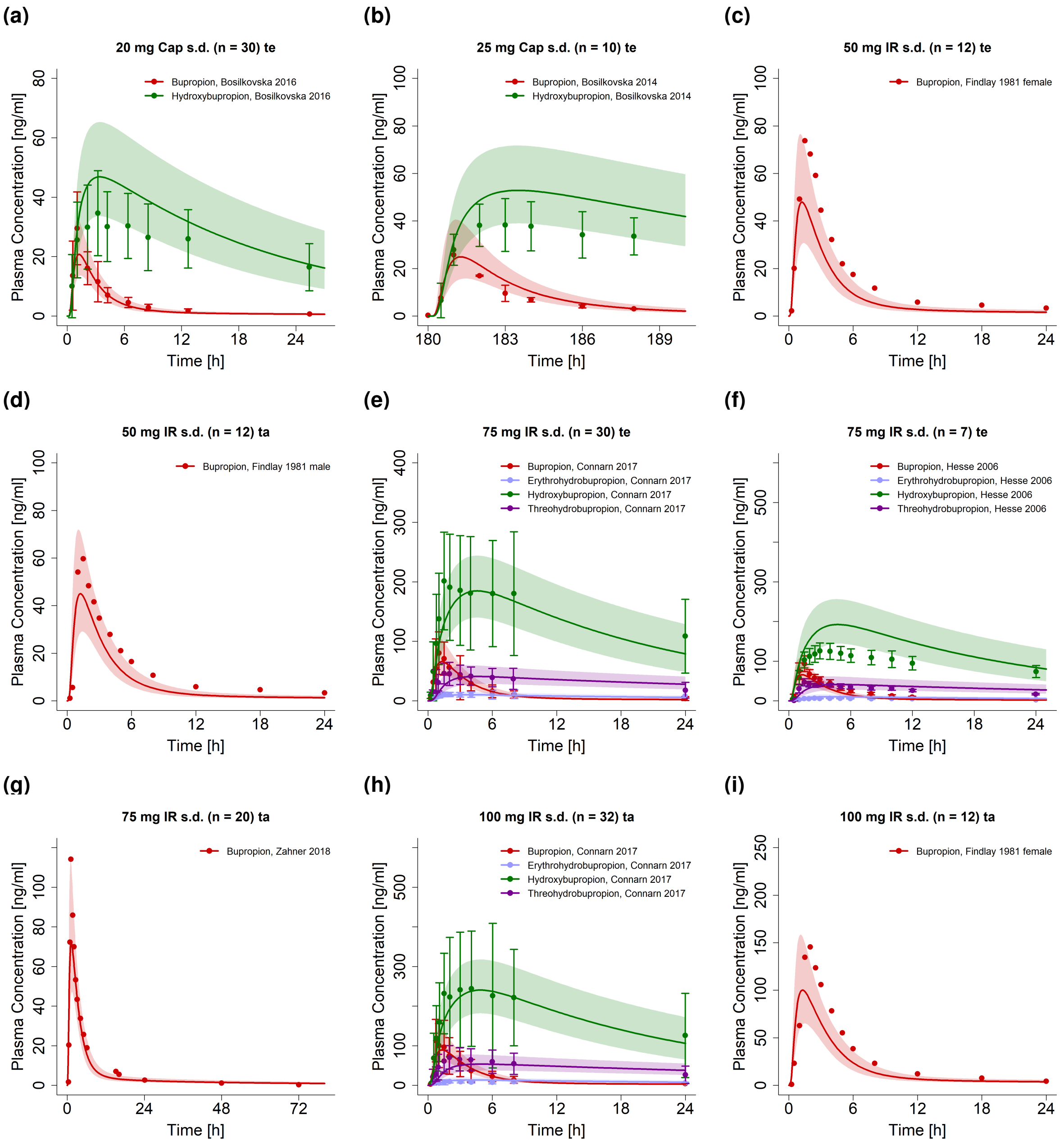

Figure S2.4.2: **Bupropion and metabolites after administration of single or multiple doses of bupropion as an immediate release formulation (part 1/3)** on a linear scale. **Cap**, capsule (Geneva cocktail [74]); **IR**, immediate release tablet; **m.d.**, multiple dose; **n**, number of individuals; **s.d.**, single dose; **ta**, training dataset; **te**, test dataset.

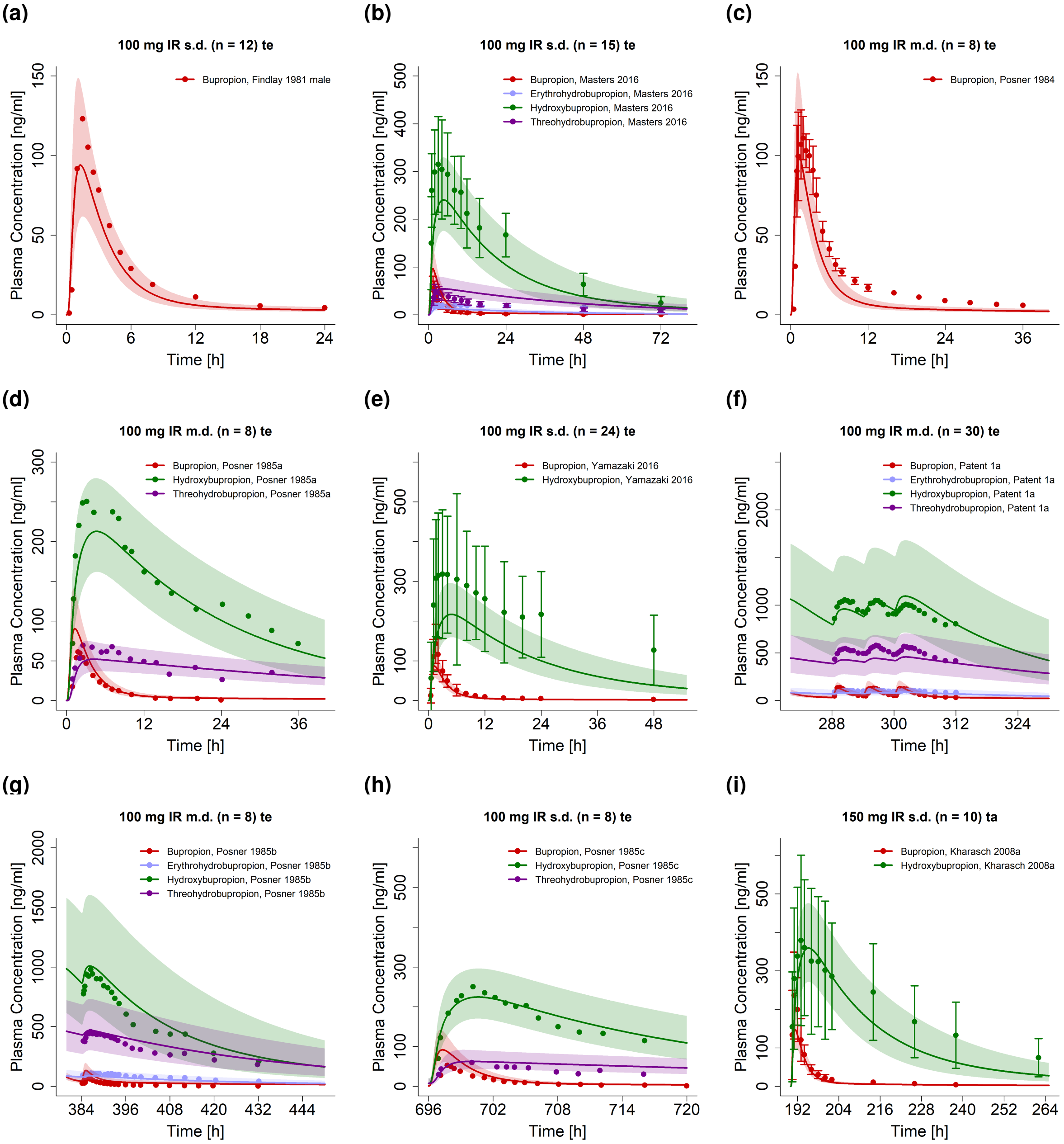

Figure S2.4.3: **Bupropion and metabolites after administration of single or multiple doses of bupropion as an immediate release formulation (part 2/3)** on a linear scale. **IR**, immediate release tablet; **m.d.**, multiple dose; **n**, number of individuals; **s.d.**, single dose; **ta**, training dataset; **te**, test dataset.

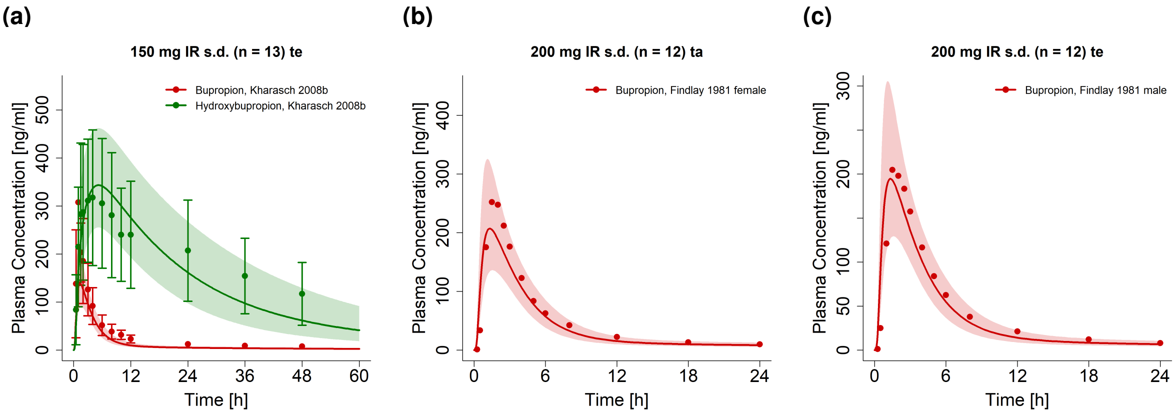

Figure S2.4.4: **Bupropion and metabolites after administration of single or multiple doses of bupropion as an immediate release formulation (part 3/3)** on a linear scale. **IR**, immediate release tablet; **m.d.**, multiple dose; **n**, number of individuals; **s.d.**, single dose; **ta**, training dataset; **te**, test dataset.

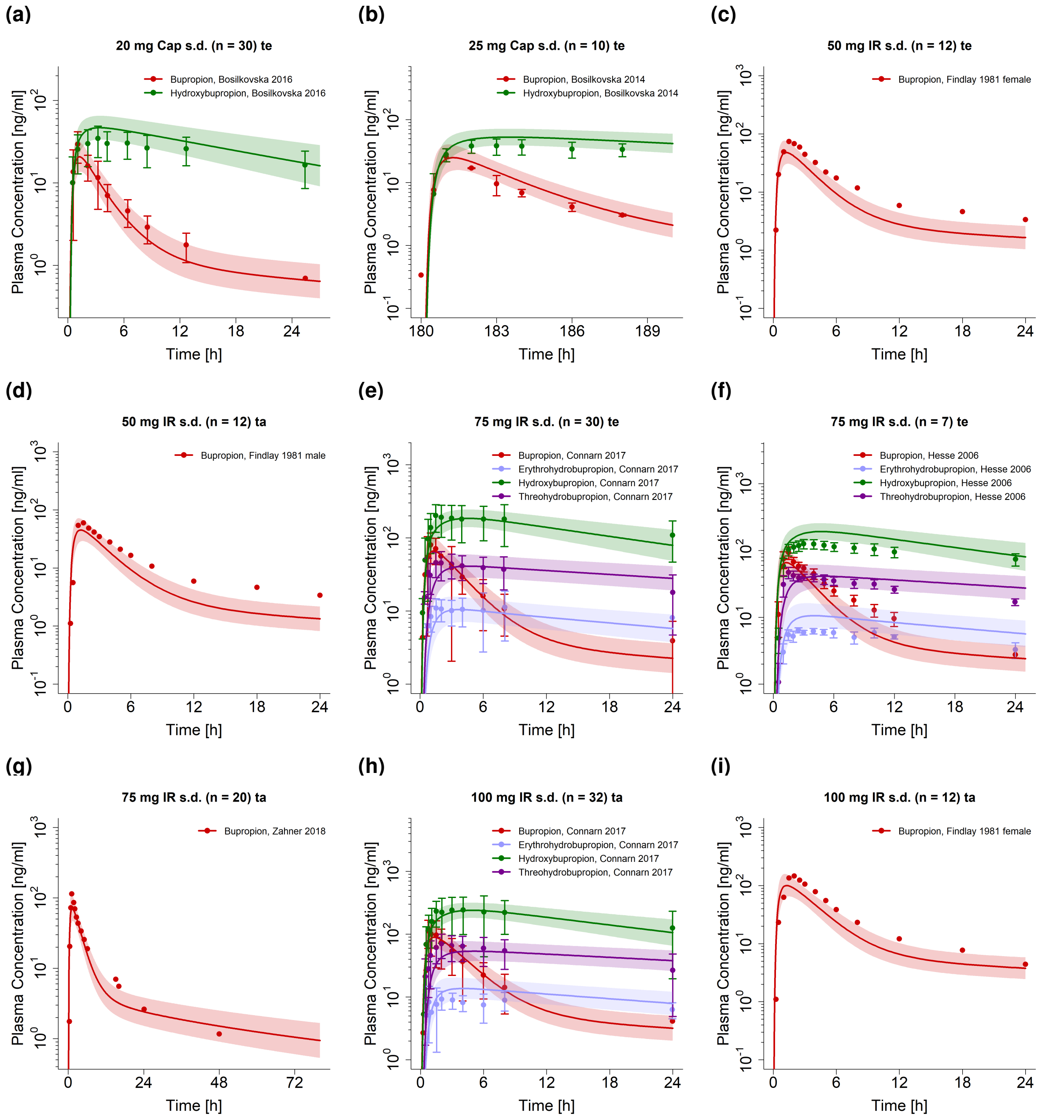

Figure S2.4.5: **Bupropion and metabolites after administration of single or multiple doses of bupropion as an immediate release formulation (part 1/3)** on a semi-logarithmic scale. **Cap**, capsule (Geneva cocktail [74]); **IR**, immediate release tablet; **m.d.**, multiple dose; **n**, number of individuals; **s.d.**, single dose; **ta**, training dataset; **te**, test dataset.

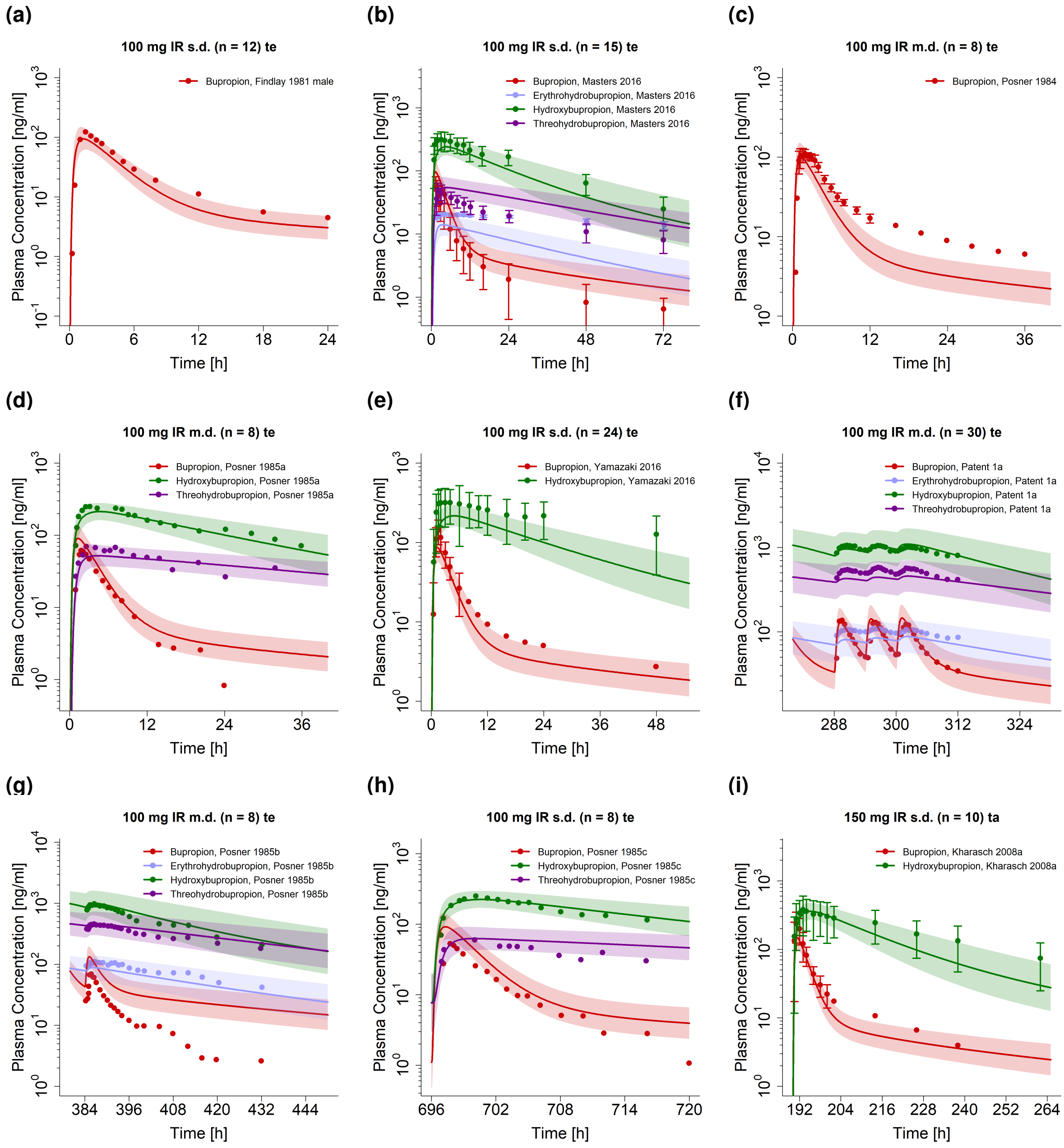

Figure S2.4.6: **Bupropion and metabolites after administration of single or multiple doses of bupropion as an immediate release formulation (part 2/3)** on a semi-logarithmic scale. **IR**, immediate release tablet; **m.d.**, multiple dose; **n**, number of individuals; **s.d.**, single dose; **ta**, training dataset; **te**, test dataset.

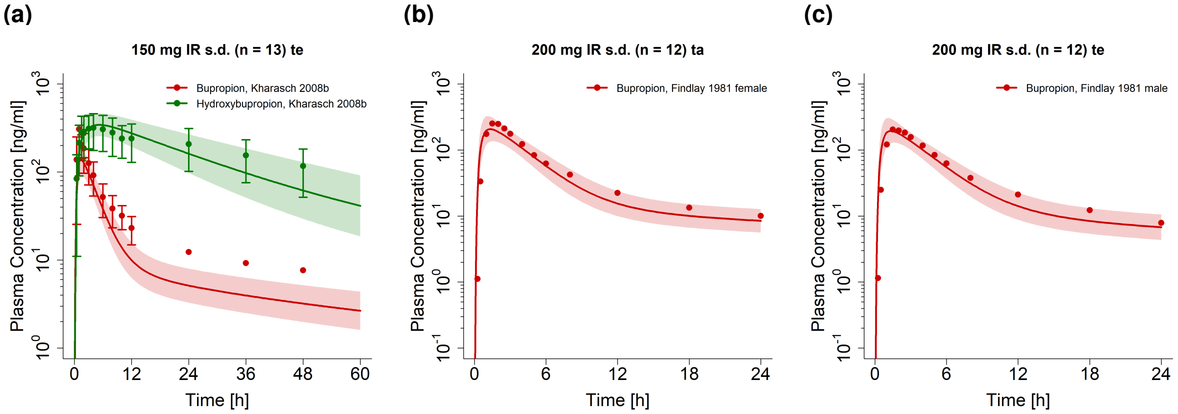

Figure S2.4.7: **Bupropion and metabolites after administration of single or multiple doses of bupropion as an immediate release formulation (part 3/3)** on a semi-logarithmic scale. **IR**, immediate release tablet; **m.d.**, multiple dose; **n**, number of individuals; **s.d.**, single dose; **ta**, training dataset; **te**, test dataset.

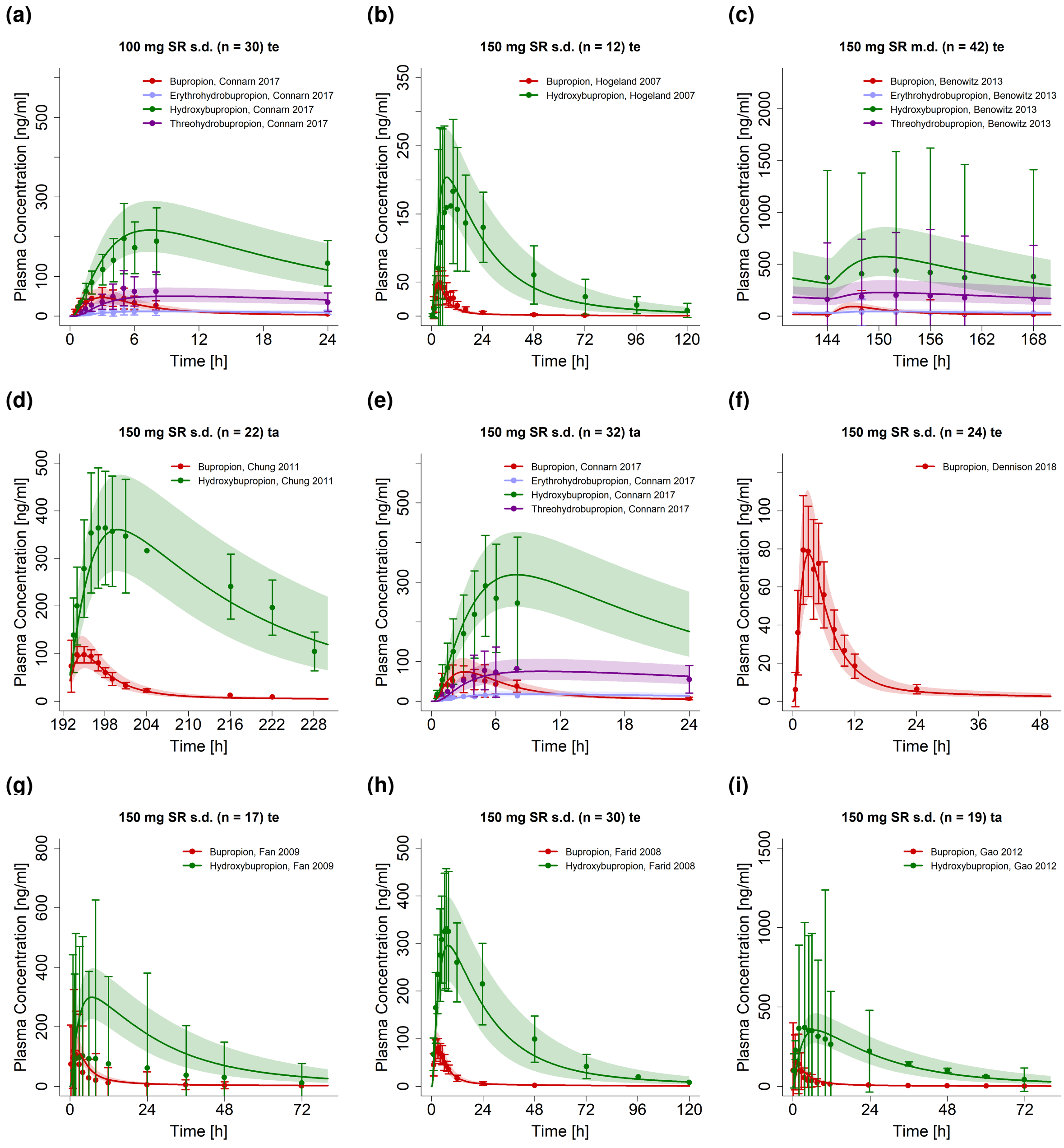

Figure S2.4.8: **Bupropion and metabolites after administration of single or multiple doses of bupropion as a sustained release formulation (part 1/3)** on a linear scale. **m.d.**, multiple dose; **n**, number of individuals; **s.d.**, single dose; **SR**, sustained release tablet; **ta**, training dataset; **te**, test dataset.

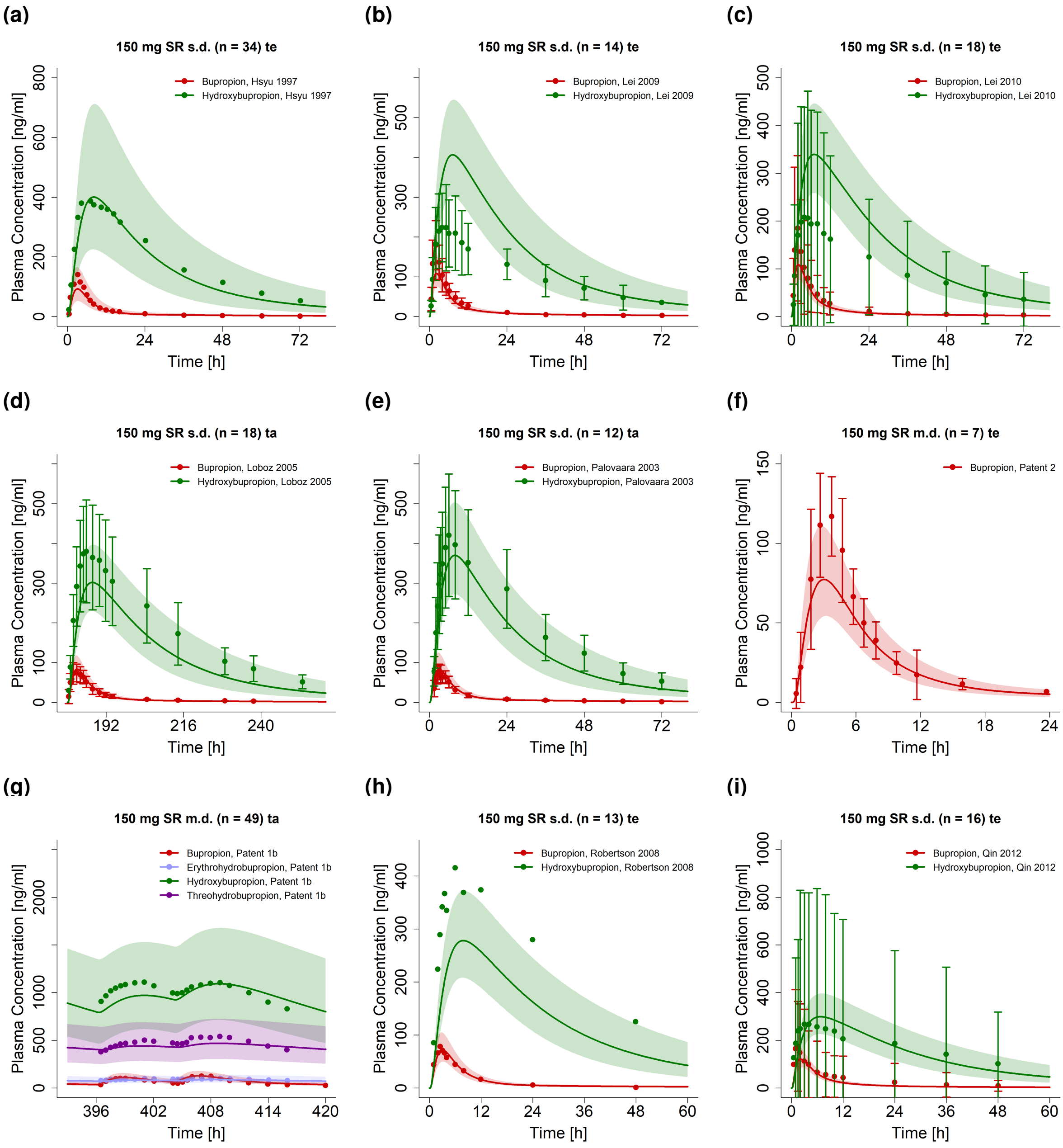

Figure S2.4.9: **Bupropion and metabolites after administration of single or multiple doses of bupropion as a sustained release formulation (part 2/3)** on a linear scale. **m.d.**, multiple dose; **n**, number of individuals; **s.d.**, single dose; **SR**, sustained release tablet; **ta**, training dataset; **te**, test dataset.

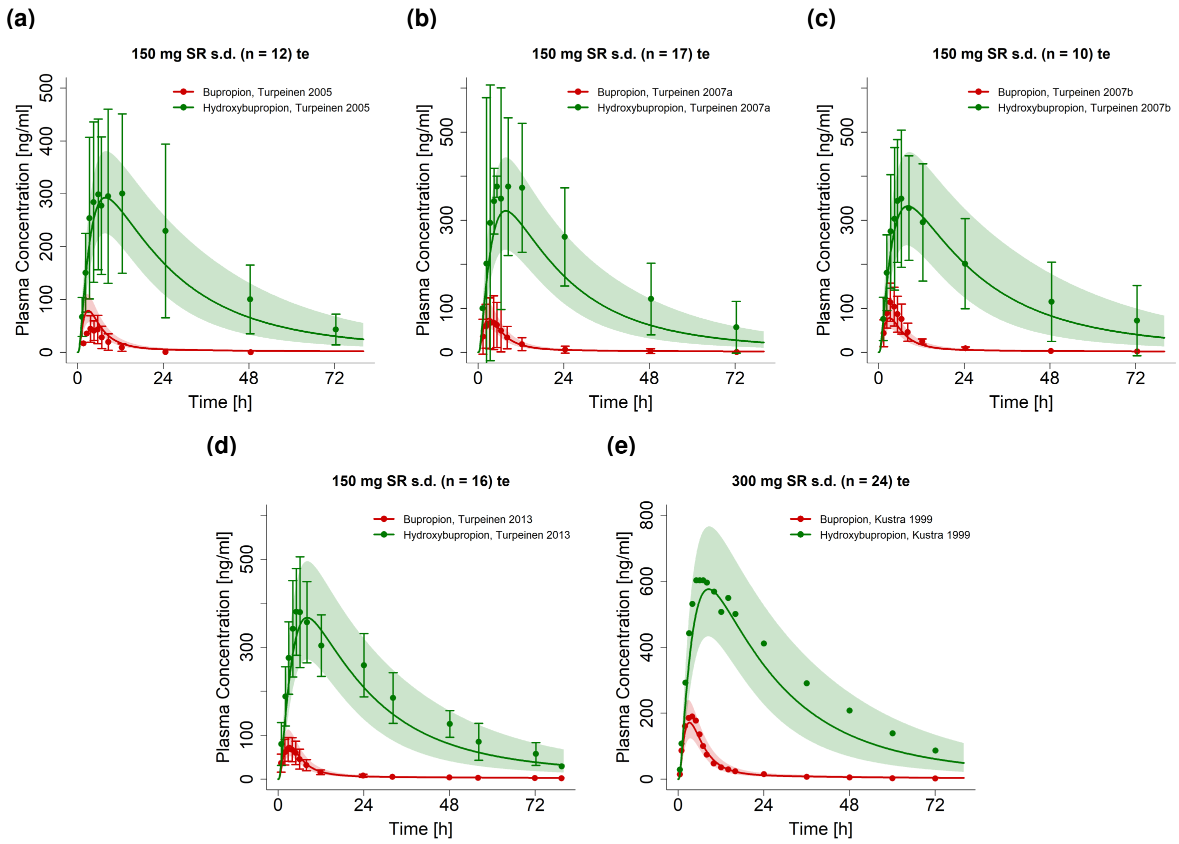

Figure S2.4.10: **Bupropion and metabolites after administration of single or multiple doses of bupropion as a sustained release formulation (part 3/3)** on a linear scale. **m.d.**, multiple dose; **n**, number of individuals; **s.d.**, single dose; **SR**, sustained release tablet; **ta**, training dataset; **te**, test dataset.

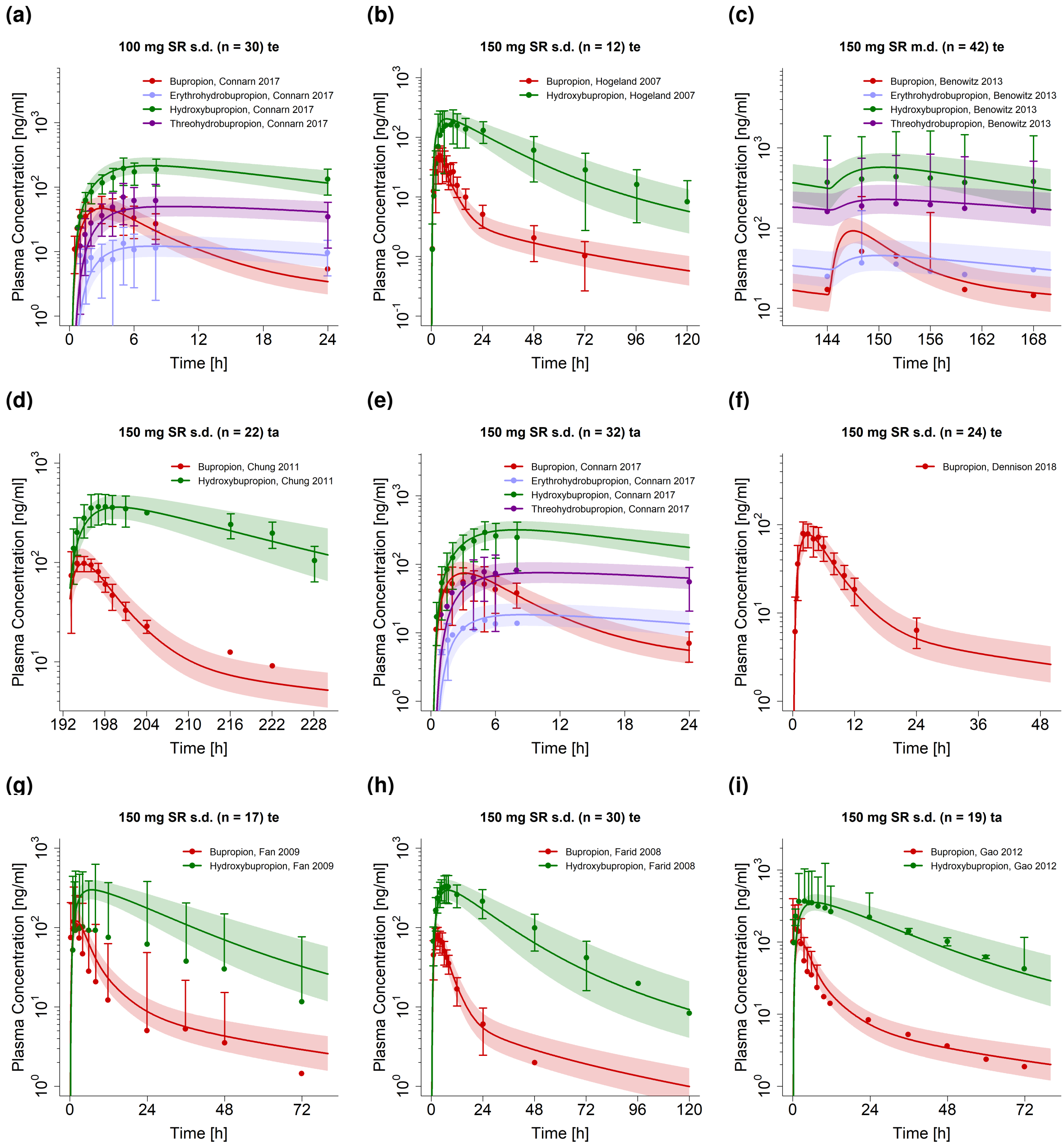

Figure S2.4.11: **Bupropion and metabolites after administration of single or multiple doses of bupropion as a sustained release formulation (part 1/3)** on a semi-logarithmic scale. **m.d.**, multiple dose; **n**, number of individuals; **s.d.**, single dose; **SR**, sustained release tablet; **ta**, training dataset; **te**, test dataset.

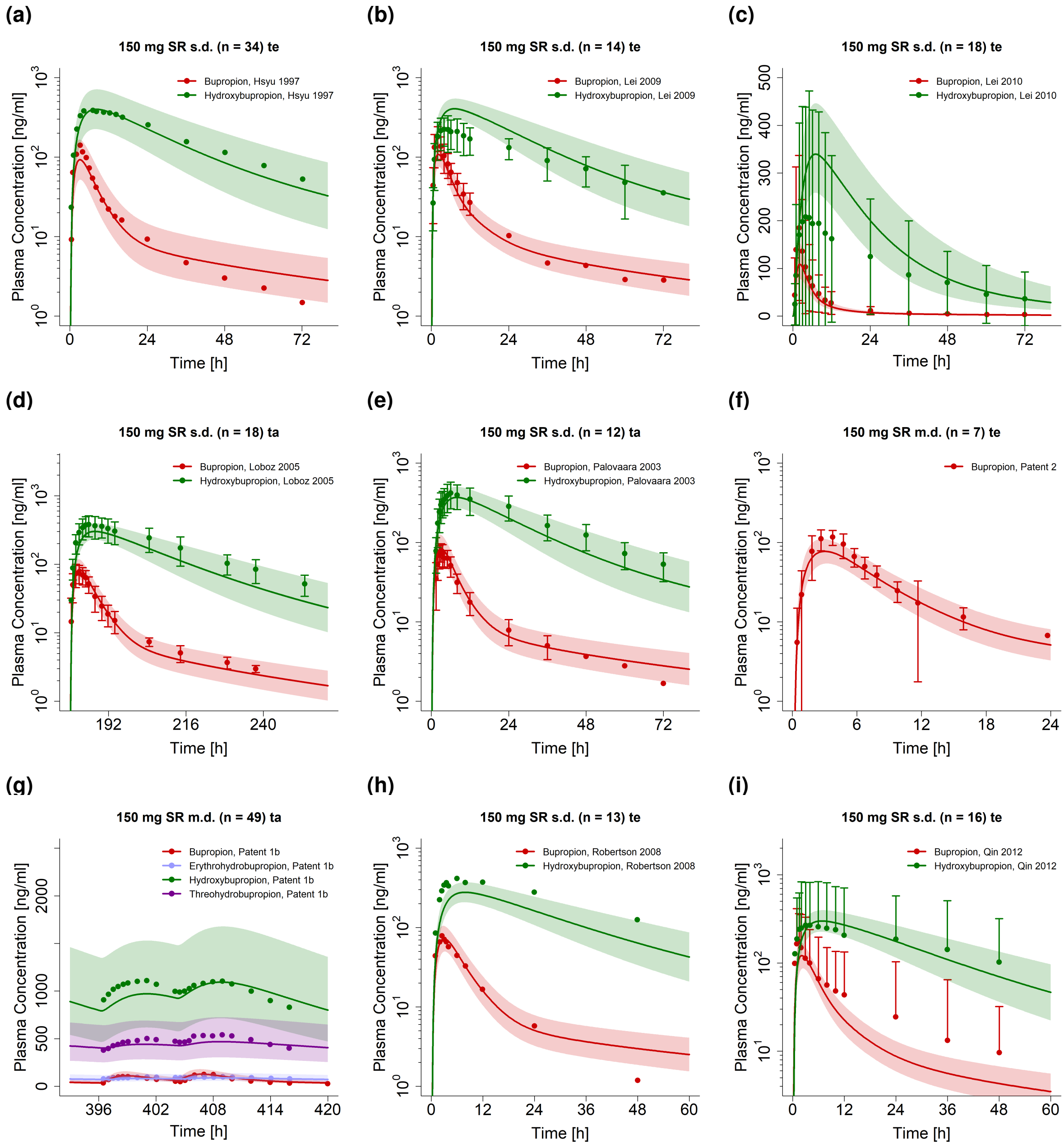

Figure S2.4.12: **Bupropion and metabolites after administration of single or multiple doses of bupropion as a sustained release formulation (part 2/3)** on a semi-logarithmic scale. **m.d.**, multiple dose; **n**, number of individuals; **s.d.**, single dose; **SR**, sustained release tablet; **ta**, training dataset; **te**, test dataset.

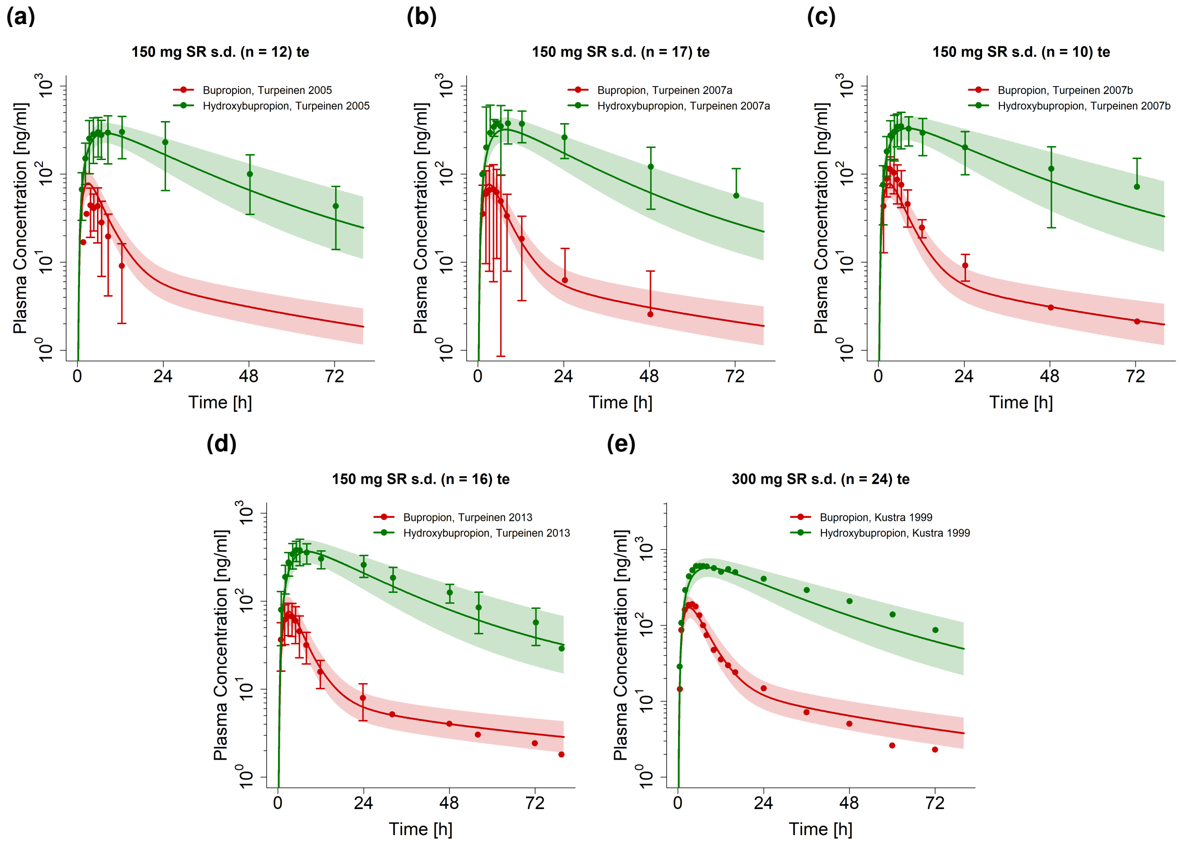

Figure S2.4.13: **Bupropion and metabolites after administration of single or multiple doses of bupropion as a sustained release formulation (part 3/3)** on a semi-logarithmic scale. **m.d.**, multiple dose; **n**, number of individuals; **s.d.**, single dose; **SR**, sustained release tablet; **ta**, training dataset; **te**, test dataset.

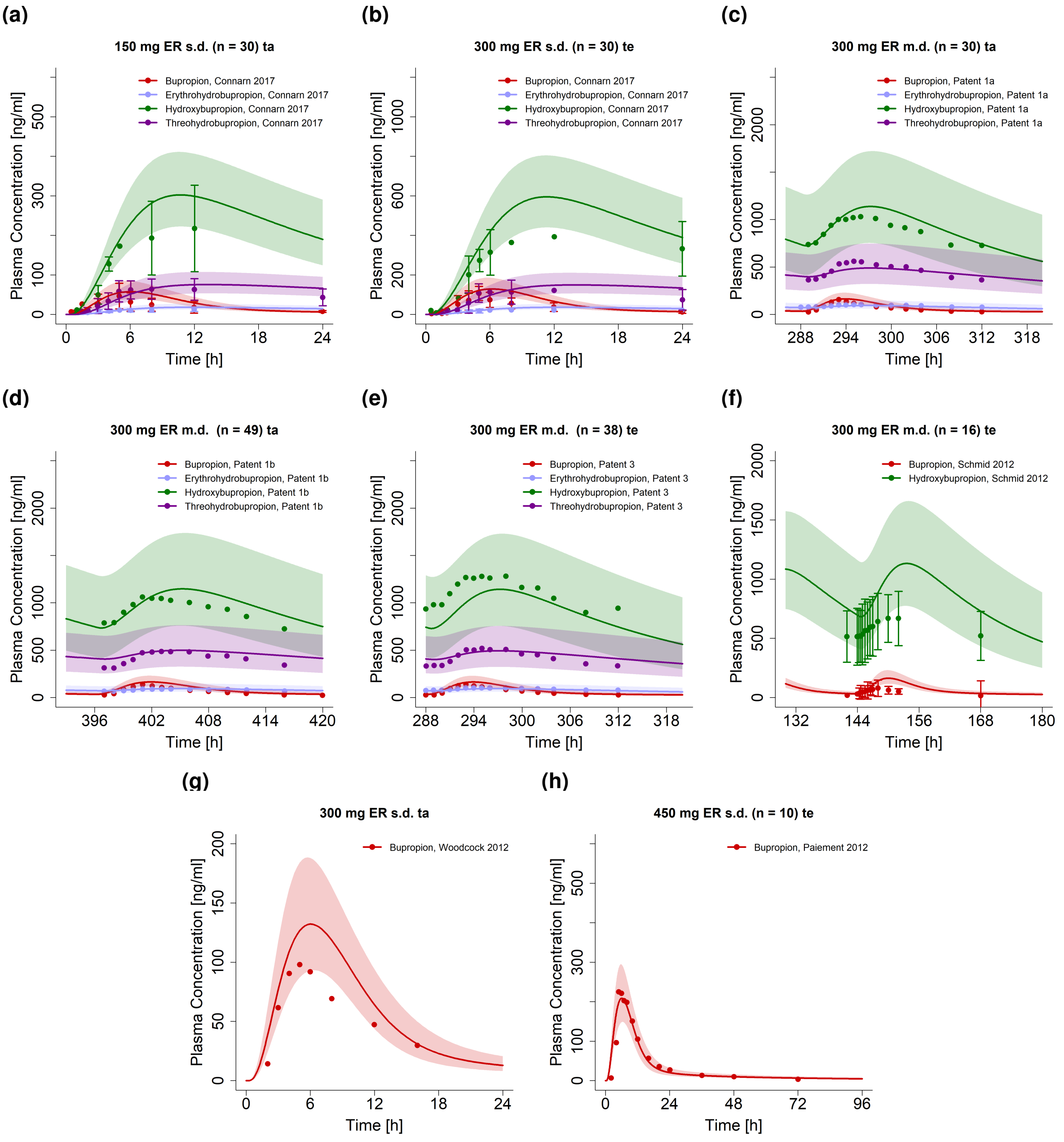

Figure S2.4.14: **Bupropion and metabolites after administration of single or multiple doses of bupropion as an extended release formulation** on a linear scale. **ER**, extended release tablet; **m.d.**, multiple dose; **n**, number of individuals; **s.d.**, single dose; **ta**, training dataset; **te**, test dataset.

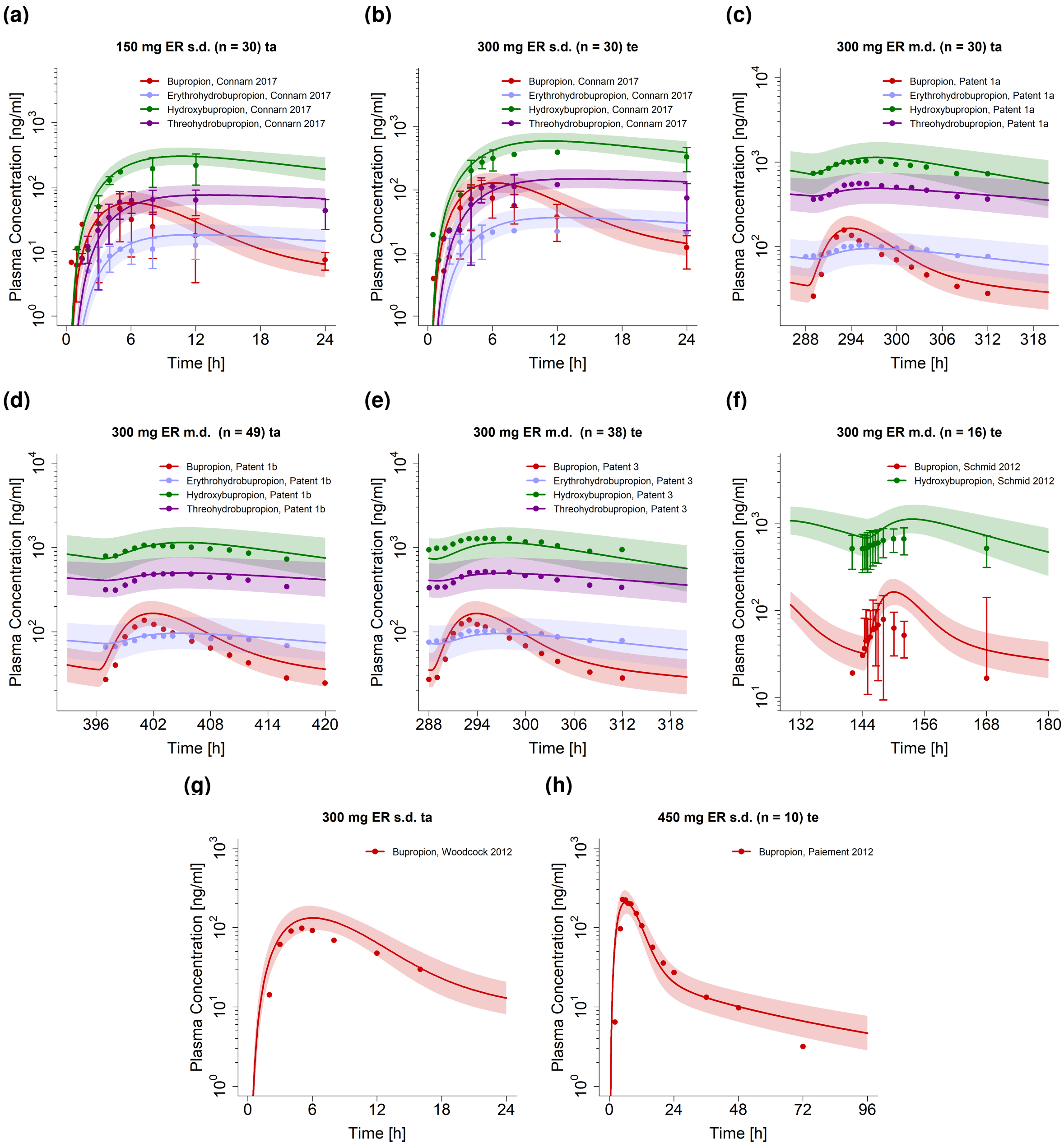

Figure S2.4.15: **Bupropion and metabolites after administration of single or multiple doses of bupropion as an extended release formulation** on a semi-logarithmic scale. **ER**, extended release tablet; **m.d.**, multiple dose; **n**, number of individuals; **s.d.**, single dose; **ta**, training dataset; **te**, test dataset.

2.5 Model evaluation

2.5.1 Predicted compared to observed concentrations goodness-of-fit plots

Following, goodness-of-fit plots of predicted compared to observed plasma concentrations of all four compounds are illustrated in Figure S2.5.16. Details on dosing regimens, study populations and literature references are listed in Table S2.1.

(a) Immediate release **(b) Sustained release** **(c) Extended release**

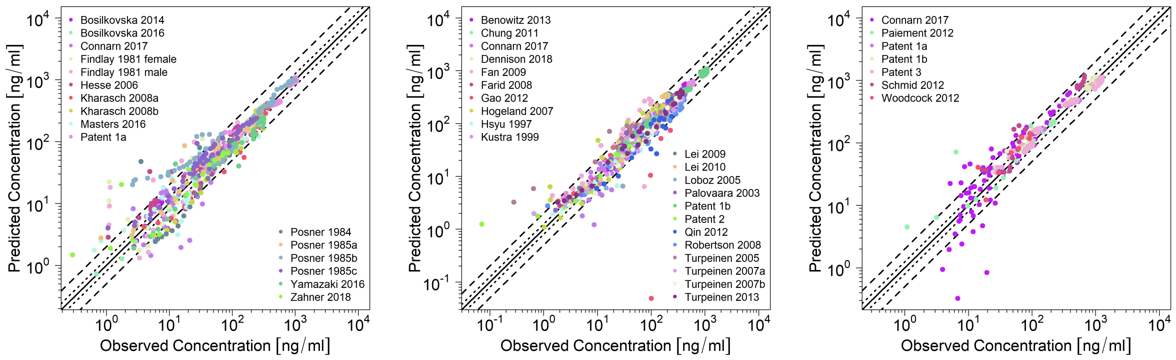

Figure S2.5.16: **Predicted compared to observed plasma concentration values.** Illustrated are values after application of (a) immediate release, (b) sustained release, and (c) extended release tablets. The solid line marks the line of identity. Dotted lines indicate 1.25-fold, dashed lines indicate 2-fold deviation.

2.5.2 Mean relative deviation of plasma concentration predictions

Table S2.3: Mean relative deviation values of bupropion, hydroxybupropion, erythrohydrobupropion and threohydrobupropion plasma concentration predictions.

Dosing	n	Compound	MRD	Compound	MRD	Dataset	Reference
20 mg Cap (s.d.)	30	Bup	1.86	HBup	1.18	ta	Bosilkovska 2016 [24]
25 mg Cap (s.d.)	10	Bup	1.40	HBup	1.36	te	Bosilkovska 2014 [25]
50 mg IR (s.d.)	12	Bup	2.03	HBup	-	te	Findlay 1981 (female) [26]
50 mg IR (s.d.)	12	Bup	2.64	HBup	-	ta	Findlay 1981 (male) [26]
75 mg IR (s.d.)	20	Bup	2.11	HBup	-	te	Zahner 2014 [27]
75 mg IR (s.d.)	7	Bup	1.62	HBup	1.57	te	Hesse 2006 [28]
75 mg IR (s.d.)	30	Bup	1.43	HBup	1.33	te	Connarn 2017 [18]
100 mg IR (s.d.)	32	Bup	1.59	HBup	1.22	ta	Connarn 2017 [18]
100 mg IR (s.d.)	12	Bup	2.10	HBup	-	ta	Findlay 1981 (female) [26]
100 mg IR (s.d.)	12	Bup	1.96	HBup	-	te	Findlay 1981 (male) [26]
100 mg IR (s.d.)	15	Bup	1.75	HBup	1.10	te	Masters 2016 [29]
100 mg IR (s.d.)	24	Bup	1.58	HBup	1.25	te	Yamazaki 2017 [30]
100 mg IR (s.d.)	8	Bup	2.30	HBup	-	te	Posner 1984 [31]
100 mg IR (s.d.)	8	Bup	1.59	HBup	1.08	te	Posner 1985a [32]
100 mg IR (m.d.)	8	Bup	2.50	HBup	1.03	te	Posner 1985b [32]
100 mg IR (s.d.)	8	Bup	1.92	HBup	1.08	te	Posner 1985c [32]
100 mg IR (m.d.)	30	Bup	1.79	HBup	1.08	ta	Patent 1a (US2006/0228415A1) [33]
150 mg IR (s.d.)	10	Bup	1.33	HBup	1.30	ta	Kharasch 2008 [34]
150 mg IR (s.d.)	13	Bup	1.71	HBup	1.14	te	Kharasch 2008b [35]
200 mg IR (s.d.)	12	Bup	2.22	HBup	-	ta	Findlay 1981 (female) [26]
200 mg IR (s.d.)	12	Bup	2.15	HBup	-	te	Findlay 1981 (male) [26]
100 mg SR (s.d.)	30	Bup	1.38	HBup	1.09	ta	Connarn 2017 [18]
100 mg SR (s.d.)	12	Bup	1.58	HBup	1.77	te	Hogeland 2007 [36]
150 mg SR (m.d.)	42	Bup	1.20	HBup	1.05	te	Benowitz 2013 [37]
150 mg SR (s.d.)	22	Bup	1.17	HBup	1.04	ta	Chung 2011 [38]
150 mg SR (s.d.)	32	Bup	1.25	HBup	1.11	ta	Connarn 2017 [18]
150 mg SR (s.d.)	24	Bup	1.13	HBup	-	te	Dennison 2018 [39]
150 mg SR (s.d.)	17	Bup	2.02	HBup	1.57	te	Fan 2009 [11]
150 mg SR (s.d.)	30	Bup	1.12	HBup	1.16	te	Farid 2008 [40]
150 mg SR (s.d.)	19	Bup	6.21	HBup	1.50	ta	Gao 2012 [12]
150 mg SR (s.d.)	34	Bup	1.25	HBup	1.33	te	Hsyu 1997 [41]
150 mg SR (s.d.)	14	Bup	1.26	HBup	1.14	te	Lei 2009 [42]
150 mg SR (s.d.)	18	Bup	1.19	HBup	1.20	te	Lei 2010 [43]
150 mg SR (s.d.)	18	Bup	1.25	HBup	1.37	ta	Loboz 2006 [44]
150 mg SR (s.d.)	12	Bup	1.22	HBup	1.16	ta	Palovaara 2003 [10]
150 mg SR (m.d.)	49	Bup	1.13	HBup	1.02	ta	Patent 1b (US2006/0228415A1) [33]
150 mg SR (m.d.)	7	Bup	1.49	HBup	-	te	Patent 2 (US8545880B2) [45]
150 mg SR (s.d.)	16	Bup	1.46	HBup	1.35	te	Qin 2012 [14]
150 mg SR (s.d.)	13	Bup	1.23	HBup	1.24	te	Robertson 2008 [46]
150 mg SR (s.d.)	12	Bup	4.02	HBup	1.12	te	Turpeinen 2005 [47]
150 mg SR (s.d.)	17	Bup	1.35	HBup	1.23	te	Turpeinen 2007a [48]
150 mg SR (s.d.)	10	Bup	1.37	HBup	1.15	te	Turpeinen 2007b [48]
150 mg SR (s.d.)	16	Bup	1.32	HBup	1.16	te	Turpeinen 2013 [49]
300 mg SR (s.d.)	24	Bup	1.35	HBup	1.13	te	Kustra 1999 [50]
150 mg ER (s.d.)	30	Bup	4.16	HBup	1.59	ta	Connarn 2017 [18]
300 mg ER (s.d.)	30	Bup	2.72	HBup	4.03	te	Connarn 2017 [18]
300 mg ER (m.d.)	30	Bup	1.16	HBup	1.02	te	Patent 1a (US2006/0228415A1) [33]
300 mg ER (m.d.)	49	Bup	1.22	HBup	1.02	ta	Patent 1b (US2006/0228415A1) [33]
300 mg ER (m.d.)	38	Bup	1.16	HBup	1.02	te	Patent 3 (US7,645,802B2) [51]
300 mg ER (m.d.)	16	Bup	1.42	HBup	1.04	te	Schmid 2012 [52]
300 mg ER (m.d.)	-	Bup	1.35	HBup	-	ta	Woodcock 2012 [53]
450 mg ER (s.d.)	10	Bup	1.97	HBup	-	te	Paiement 2012 [54]
75 mg IR (s.d.)	7	EBup	1.81	TBup	1.40	te	Hesse 2006 [28]
75 mg IR (s.d.)	32	EBup	1.55	TBup	2.39	te	Connarn 2017 [18]
100 mg IR (s.d.)	30	EBup	2.29	TBup	2.19	ta	Connarn 2017 [18]
100 mg IR (s.d.)	15	EBup	1.77	TBup	1.40	te	Masters 2016 [29]
100 mg IR (s.d.)	8	EBup	-	TBup	1.21	te	Posner 1985a [32]
100 mg IR (m.d.)	8	EBup	1.20	TBup	1.03	te	Posner 1985b [32]

Table S2.3: Mean relative deviation values of bupropion, hydroxybupropion, erythrohydrobupropion and threohydrobupropion plasma concentration predictions. (continued)

Dosing	n	Compound	MRD	Compound	MRD	Dataset	Reference
100 mg IR (m.d.)	8	EBup	-	TBup	1.19	te	Posner 1985c [32]
100 mg IR (m.d.)	30	EBup	1.13	TBup	1.03	ta	Patent 1a (US2006/0228415A1) [33]
100 mg SR (s.d.)	30	EBup	3.00	TBup	1.41	te	Connarn 2017 [18]
150 mg SR (m.d.)	42	EBup	1.13	TBup	1.04	te	Benowitz 2013 [37]
150 mg SR (s.d.)	32	EBup	1.87	TBup	1.39	ta	Connarn 2017 [18]
150 mg SR (m.d.)	49	EBup	1.05	TBup	1.01	ta	Patent 1b (US2006/0228415A1) [33]
150 mg ER (s.d.)	30	EBup	1.78	TBup	1.83	ta	Connarn 2017 [18]
300 mg ER (s.d.)	30	EBup	1.57	TBup	1.49	te	Connarn 2017 [18]
300 mg ER (m.d.)	30	EBup	1.04	TBup	1.02	te	Patent 1a (US2006/0228415A1) [33]
300 mg ER (m.d.)	49	EBup	1.10	TBup	1.05	ta	Patent 1b (US2006/0228415A1) [33]
300 mg ER (m.d.)	38	EBup	1.04	TBup	1.04	te	Patent 3 (US7,645,802B2) [51]
	**Mean**		**1.51 (1.01–6.21)**				
	**Median**		**1.24 (1.01–6.21)**				
			**83.06% (103/124) $\leq$ 2**				

Bup, bupropion; **Cap**, capsule (Geneva cocktail capsule [74]); **EBup**, erythrohydrobupropion; **ER**, extended release tablet formulation, **HBup**, hydroxybupropion; **IR**, immediate release tablet formulation; **m.d.**, multiple dose; **MRD**, mean relative deviation; **n**, number of individuals studied; **s.d.**, single dose; **SR**, sustained release formulation; **TBup**, threohydrobupropion; **ta**, training dataset; **te**, test dataset; **-**, not available.

2.5.3 AUC and C_{max} goodness-of-fit plots

Following, goodness-of-fit plots of predicted compared to observed AUC and C_{max} values for every study are illustrated in Figures S2.5.17 and S2.5.18. Line of identity and 2.0-fold acceptance limits are shown as black dashed lines. The 1.25-fold limits are shown as black dotted lines.

Details on dosing regimens, study populations and literature references are listed in Table S2.1. Predicted and observed PK parameters are summarized in Table S2.4.

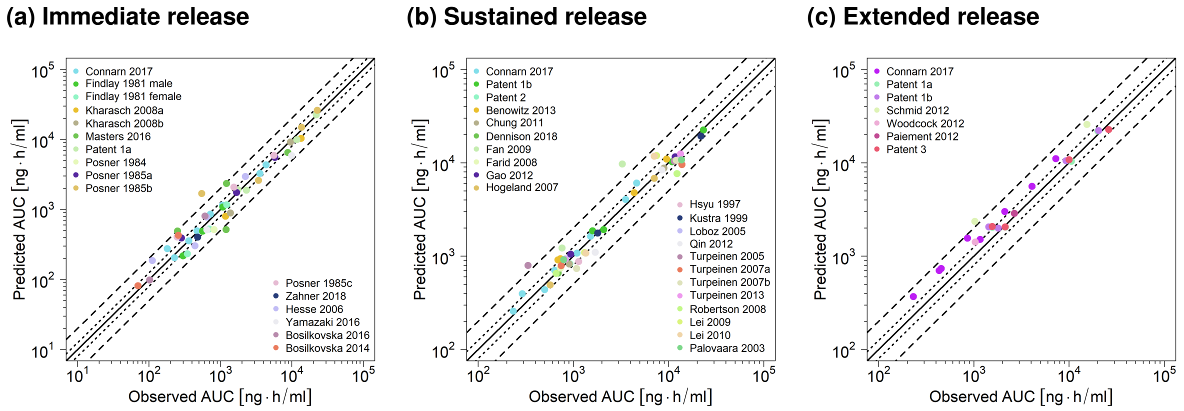

Figure S2.5.17: **Predicted compared to observed plasma AUC_{last} values.** Illustrated are values after application of (a) immediate release, (b) sustained release, and (c) extended release tablets. The solid line marks the line of identity. Dotted lines indicate 1.25-fold, dashed lines indicate 2-fold deviation. **AUC**, area under the plasma concentration-time curve.

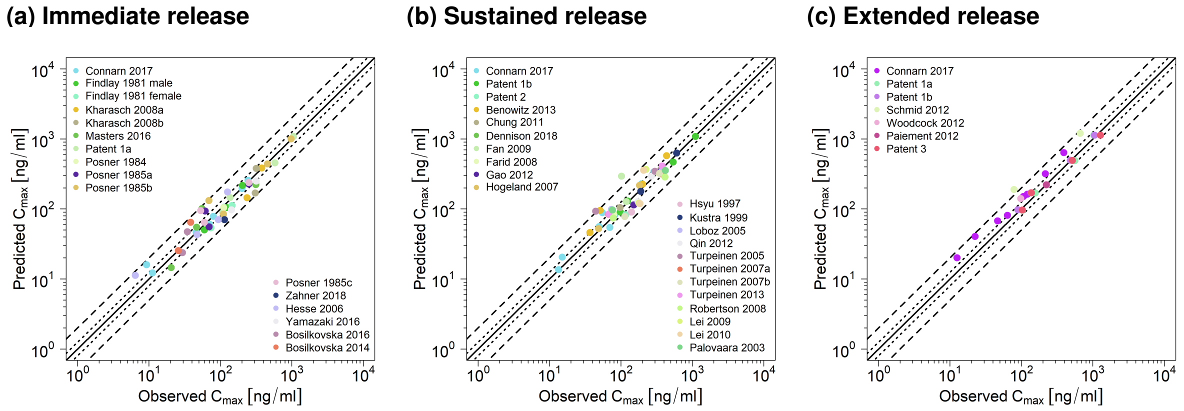

Figure S2.5.18: **Predicted compared to observed plasma C_{max} values.** Illustrated are values after application of (a) immediate release, (b) sustained release, and (c) extended release tablets. The solid line marks the line of identity. Dotted lines indicate 1.25-fold, dashed lines indicate 2-fold deviation. **C_{max}**, maximum plasma concentration.

2.5.4 Geometric mean fold error of predicted AUC and C_{max} values

Table S2.4: Predicted and observed AUC_{last} and C_{max} values of bupropion, hydroxybupropion, erythrohydrobupropion and threohydrobupropion plasma concentrations.

Dosing	n	Compound	AUC_{last} pred [ng*h/ml]	AUC_{last} obs [ng*h/ml]	AUC_{last} pred/obs	C_{max} pred [ng/ml]	C_{max} obs [ng/ml]	C_{max} pred/obs	Dataset	Reference
20 mg Cap (s.d.)	30	Bup	98.29	102.71	0.96	23.68	29.54	0.80	ta	Bosilkovska 2016 [24]
25 mg Cap (s.d.)	10	Bup	81.20	70.34	1.15	25.35	25.74	0.98	te	Bosilkovska 2014 [25]
50 mg IR (s.d.)	12	Bup	232.54	346.19	0.67	53.64	73.78	0.73	te	Findlay 1981 (female) [26]
50 mg IR (s.d.)	12	Bup	217.60	301.43	0.72	50.12	59.67	0.84	ta	Findlay 1981 (male) [26]
75 mg IR (s.d.)	20	Bup	401.99	481.30	0.84	70.29	114.17	0.62	te	Zahner 2014 [27]
75 mg IR (s.d.)	7	Bup	302.02	440.60	0.69	70.10	93.50	0.75	te	Hesse 2006 [28]
75 mg IR (s.d.)	30	Bup	354.49	361.90	0.98	78.04	80.17	0.97	te	Connarn 2017 [18]
100 mg IR (s.d.)	32	Bup	495.78	471.50	1.05	105.74	113.02	0.94	ta	Connarn 2017 [18]
100 mg IR (s.d.)	12	Bup	526.62	694.87	0.76	111.74	145.59	0.77	ta	Findlay 1981 (female) [26]
100 mg IR (s.d.)	12	Bup	489.31	562.72	0.87	104.48	123.13	0.85	te	Findlay 1981 (male) [26]
100 mg IR (s.d.)	15	Bup	491.78	250.82	1.96	101.26	55.38	1.83	te	Masters 2016 [29]
100 mg IR (s.d.)	24	Bup	512.69	648.61	0.79	96.95	143.0	0.68	te	Yamazaki 2017 [30]
100 mg IR (s.d.)	8	Bup	514.20	813.57	0.63	104.69	111.0	0.94	te	Posner 1984 [31]
100 mg IR (s.d.)	8	Bup	390.50	286.01	1.37	92.88	61.22	1.51	te	Posner 1985a [32]
100 mg IR (m.d.)	8	Bup	1681.00	549.73	3.06	131.59	69.30	1.90	te	Posner 1985b [32]
100 mg IR (s.d.)	8	Bup	404.32	248.92	1.62	94.40	52.89	1.78	te	Posner 1985c [32]
100 mg IR (m.d.)	30	Bup	1765.08	1765.08	1.08	145.51	136.69	1.06	ta	Patent 1a (US2006/0228415A1) [33]
150 mg IR (s.d.)	10	Bup	798.66	1182.40	0.68	144.23	235.94	0.61	ta	Kharasch 2008 [34]
150 mg IR (s.d.)	13	Bup	888.32	1386.28	0.64	168.77	307.63	0.55	te	Kharasch 2008b [35]
200 mg IR (s.d.)	12	Bup	1165.09	1225.94	0.95	230.57	252.23	0.91	ta	Findlay 1981 (female) [26]
200 mg IR (s.d.)	12	Bup	1165.09	1080.86	1.00	215.78	204.63	1.05	te	Findlay 1981 (male) [26]
100 mg SR (s.d.)	30	Bup	437.68	501.70	0.87	55.89	49.59	1.13	te	Connarn 2017 [18]
100 mg SR (s.d.)	12	Bup	492.31	572.17	0.86	52.32	48.77	1.07	te	Hogeland 2007 [36]
150 mg SR (m.d.)	42	Bup	910.01	689.79	1.32	95.34	51.32	1.86	te	Benowitz 2013 [37]
150 mg SR (s.d.)	22	Bup	822.18	909.01	0.90	88.34	55.37	1.60	ta	Chung 2011 [38]
150 mg SR (s.d.)	32	Bup	705.10	639.64	1.10	82.75	116.92	0.71	ta	Connarn 2017 [18]
150 mg SR (s.d.)	24	Bup	657.81	693.30	0.95	95.34	51.32	1.86	te	Dennison 2018 [39]
150 mg SR (s.d.)	17	Bup	1221.28	763.62	1.60	104.29	97.73	1.07	te	Fan 2009 [11]
150 mg SR (s.d.)	30	Bup	743.43	734.89	1.01	82.66	79.34	1.04	te	Farid 2008 [40]
150 mg SR (s.d.)	19	Bup	1048.12	941.59	1.11	126.61	118.63	1.07	ta	Gao 2012 [12]
150 mg SR (s.d.)	34	Bup	873.80	1141.13	0.77	114.07	151.68	0.75	te	Hsyu 1997 [41]
150 mg SR (s.d.)	14	Bup	1092.76	1319.17	0.83	90.21	140.82	0.64	te	Lei 2009 [42]
150 mg SR (s.d.)	18	Bup	1075.17	1358.91	0.79	178.25	189.50	0.94	te	Lei 2010 [43]
150 mg SR (s.d.)	18	Bup	932.99	832.02	1.12	98.22	74.04	1.33	ta	Loboz 2006 [44]
150 mg SR (s.d.)	12	Bup	916.46	793.90	1.15	96.64	76.52	1.26	ta	Palovaara 2003 [10]
150 mg SR (m.d.)	49	Bup	1861.39	1599.96	1.16	126.61	174.87	0.72	ta	Patent 1b (US2006/0228415A1) [33]
150 mg SR (m.d.)	7	Bup	827.72	920.60	0.90	92.50	44.25	2.09	te	Patent 2 (US8545880B2) [45]
150 mg SR (s.d.)	16	Bup	1097.92	1700.06	0.65	83.87	67.24	1.25	te	Qin 2012 [14]

Table S2.4: Predicted and observed AUC_{last} and C_{max} values of bupropion, hydroxybupropion, erythrohydrobupropion and threohydrobupropion plasma concentrations. (continued)

**Dosing**	**n**	**Compound**	**AUC_{last} pred [ng*h/ml]**	**AUC_{last} obs [ng*h/ml]**	**AUC_{last} pred/obs**	**C_{max} pred [ng/ml]**	**C_{max} obs [ng/ml]**	**C_{max} pred/obs**	**Dataset**	**Reference**
150 mg SR (s.d.)	13	Bup	651.49	659.39	0.99	77.90	113.75	0.68	te	Robertson 2008 [46]
150 mg SR (s.d.)	12	Bup	795.88	335.35	2.37	84.26	67.63	1.25	te	Turpeinen 2005 [47]
150 mg SR (s.d.)	17	Bup	788.51	746.66	1.06	95.34	53.56	1.78	te	Turpeinen 2007a [48]
150 mg SR (s.d.)	10	Bup	734.31	1082.42	0.68	73.91	78.49	0.94	te	Turpeinen 2007b [48]
150 mg SR (s.d.)	16	Bup	920.83	817.53	1.13	120.32	179.49	0.67	te	Turpeinen 2013 [49]
300 mg SR (s.d.)	24	Bup	1767.98	1803.47	0.98	52.32	48.77	1.07	te	Kustra 1999 [50]
150 mg ER (s.d.)	30	Bup	701.46	434.50	1.61	67.23	46.49	1.45	ta	Connarn 2017 [18]
300 mg ER (s.d.)	30	Bup	1552.49	860.70	1.80	150.08	106.61	1.41	te	Connarn 2017 [18]
300 mg ER (m.d.)	30	Bup	2031.86	1571.33	1.29	168.64	156.81	1.08	te	Patent 1a (US2006/0228415A1) [33]
300 mg ER (m.d.)	49	Bup	2051.28	1436.28	1.43	169.62	136.45	1.24	ta	Patent 1b (US2006/0228415A1) [33]
300 mg ER (m.d.)	38	Bup	2078.06	1565.12	1.33	169.15	138.19	1.22	te	Patent 3 (US7,645,802B2) [45]
300 mg ER (m.d.)	16	Bup	2355.85	1029.53	2.29	188.63	79.02	2.39	te	Schmid 2012 [52]
300 mg ER (m.d.)	-	Bup	1413.04	1036.09	1.36	140.19	97.94	1.43	ta	Woodcock 2012 [53]
450 mg ER (s.d.)	20	Bup	2870.69	2674.37	1.07	221.19	224.68	0.98	ta	Paiement 2012 [54]
20 mg Cap (s.d.)	30	HBup	801.40	607.74	1.32	195.62	201.48	0.97	ta	Bosilkovska 2016 [24]
25 mg Cap (s.d.)	10	HBup	427.83	257.69	1.66	256.21	244.02	1.05	te	Bosilkovska 2014 [25]
75 mg IR (s.d.)	7	HBup	2953.13	2239.20	1.32	1079.21	1055.87	1.02	te	Hesse 2006 [28]
75 mg IR (s.d.)	30	HBup	3269.46	3591.89	0.91	221.85	314.78	0.70	te	Connarn 2017 [18]
100 mg IR (s.d.)	32	HBup	4332.10	4370.16	0.99	383.99	379.17	1.01	ta	Connarn 2017 [18]
100 mg IR (s.d.)	15	HBup	6471.74	8796.79	0.74	237.18	250.64	0.95	te	Masters 2016 [29]
100 mg IR (s.d.)	24	HBup	5853.48	9995.87	0.59	64.28	38.33	1.68	te	Yamazaki 2017 [30]
100 mg IR (s.d.)	8	HBup	5498.67	5803.33	0.95	249.20	318.00	0.78	te	Posner 1985a [32]
100 mg IR (m.d.)	8	HBup	26056.97	22722.51	1.15	377.70	317.49	1.19	te	Posner 1985b [32]
100 mg IR (s.d.)	8	HBup	5890.56	5597.22	1.05	47.21	34.64	1.36	te	Posner 1985c [32]
100 mg IR (m.d.)	30	HBup	22454.16	22075.43	1.02	173.88	126.04	1.38	ta	Patent 1a (US2006/0228415A1) [33]
150 mg IR (s.d.)	10	HBup	9160.32	9618.74	0.95	227.45	250.39	0.91	ta	Kharasch 2008 [34]
150 mg IR (s.d.)	13	HBup	10351.70	13530.75	0.77	997.47	979.94	1.02	te	Kharasch 2008b [**?**]
100 mg SR (s.d.)	30	HBup	4045.70	3549.10	1.14	228.03	194.98	1.17	te	Connarn 2017 [18]
100 mg SR (s.d.)	12	HBup	6811.49	7084.82	0.96	216.14	182.94	1.18	te	Hogeland 2007 [36]
150 mg SR (m.d.)	42	HBup	10957.92	9547.77	1.15	573.84	436.84	1.31	te	Benowitz 2013 [37]
150 mg SR (s.d.)	22	HBup	8738.64	8936.26	0.98	366.16	363.64	1.01	ta	Chung 2011 [38]
150 mg SR (s.d.)	32	HBup	6041.12	4633.27	1.30	573.84	436.84	1.31	ta	Connarn 2017 [18]
150 mg SR (s.d.)	17	HBup	9661.12	3268.52	2.96	366.16	363.64	1.01	te	Fan 2009 [11]
150 mg SR (s.d.)	30	HBup	10502.50	12055.85	0.87	293.00	102.05	2.87	te	Farid 2008 [40]
150 mg SR (s.d.)	19	HBup	11539.68	11762.50	0.98	327.37	331.16	0.99	te	Gao 2012 [12]
150 mg SR (s.d.)	34	HBup	10166.26	13447.39	0.76	216.14	182.94	1.18	te	Hsyu [**?**]
150 mg SR (s.d.)	14	HBup	11893.87	7482.04	1.59	624.98	602.58	1.04	te	Lei 2009 [42]
150 mg SR (s.d.)	18	HBup	11704.75	7112.73	1.65	353.25	379.78	0.93	te	Lei 2010 [43]
150 mg SR (s.d.)	18	HBup	10854.40	12976.72	0.84	293.00	267.66	1.09	ta	Loboz 2006 [44]
150 mg SR (s.d.)	12	HBup	10727.87	13747.54	0.78	353.29	420.35	0.84	ta	Palovaara 2003 [10]

Table S2.4: Predicted and observed AUC_{last} and C_{max} values of bupropion, hydroxybupropion, erythrohydrobupropion and threohydrobupropion plasma concentrations. (continued)

**Dosing**	**n**	**Compound**	**AUC_{last} pred [ng*h/ml]**	**AUC_{last} obs [ng*h/ml]**	**AUC_{last} pred/obs**	**C_{max} pred [ng/ml]**	**C_{max} obs [ng/ml]**	**C_{max} pred/obs**	**Dataset**	**Reference**
150 mg SR (m.d.)	49	HBup	22365.49	23315.14	0.96	343.79	300.46	1.14	ta	Patent 1b (US2006/0228415A1) [33]
150 mg SR (s.d.)	16	HBup	8372.74	8540.70	0.98	315.19	376.14	0.84	te	Qin 2012 [14]
150 mg SR (s.d.)	13	HBup	7644.43	12305.92	0.62	322.75	348.93	0.92	te	Robertson 2008 [46]
150 mg SR (s.d.)	12	HBup	10397.75	11506.36	0.90	409.26	380.38	1.08	te	Turpeinen 2005 [47]
150 mg SR (s.d.)	17	HBup	9557.71	13862.09	0.69	573.84	435.00	1.32	te	Turpeinen 2007 [48]
150 mg SR (s.d.)	10	HBup	10597.39	12076.82	0.88	285.43	415.53	0.69	te	Turpeinen 2007 [48]
150 mg SR (s.d.)	16	HBup	12401.84	13350.60	0.93	364.92	223.79	1.63	te	Turpeinen 2013 [49]
300 mg SR (s.d.)	24	HBup	19212.14	21964.35	0.87	339.72	386.80	0.88	te	Kustra 1999 [50]
150 mg ER (s.d.)	30	HBup	5583.86	4088.58	1.37	318.33	217.43	1.46	ta	Connarn 2017 [18]
300 mg ER (s.d.)	30	HBup	11061.39	7266.99	1.52	635.02	392.91	1.62	te	Connarn 2017 [18]
300 mg ER (m.d.)	30	HBup	21885.82	20133.46	1.09	1123.03	1030.00	1.09	te	Patent 1a (US2006/0228415A1) [33]
300 mg ER (m.d.)	49	HBup	22072.35	20458.99	1.08	1130.27	1061.85	1.06	ta	Patent 1b (US2006/0228415A1) [33]
300 mg ER (m.d.)	38	HBup	22654.20	26187.27	0.87	1126.80	1280.80	0.88	te	Patent 3 (US7,645,802B2) [45]
300 mg ER (m.d.)	16	HBup	25727.64	15469.07	1.66	1196.56	668.29	1.79	te	Schmid 2012 [52]
75 mg IR (s.d.)	7	EBup	186.61	111.51	1.67	14.50	20.45	0.71	te	Hesse 2006 [28]
75 mg IR (s.d.)	30	EBup	204.83	228.25	0.90	12.19	11.26	1.08	te	Connarn 2017 [18]
100 mg IR (s.d.)	32	EBup	275.48	180.72	1.52	15.90	9.24	1.72	ta	Connarn 2017 [18]
100 mg IR (s.d.)	15	EBup	514.26	1205.83	0.43	88.37	108.50	0.81	te	Masters 2016 [29]
100 mg IR (m.d.)	30	EBup	1877.36	2312.75	0.81	83.97	110.63	0.76	ta	Patent 1a (US2006/0228415A1) [33]
100 mg IR (s.d.)	8	EBup	2586.00	3430.72	0.75	11.27	6.43	1.75	te	Posner 1985b [32]
100 mg SR (s.d.)	30	EBup	256.95	234.68	1.09	13.61	13.42	1.01	te	Connarn 2017 [18]
150 mg SR (m.d.)	42	EBup	936.91	733.66	1.28	45.94	36.84	1.25	te	Benowitz 2013 [37]
150 mg SR (s.d.)	32	EBup	394.99	292.35	1.35	20.47	15.15	1.35	ta	Connarn 2017 [18]
150 mg SR (m.d.)	49	EBup	1925.27	2084.16	0.92	89.99	98.09	0.92	ta	Patent 1b (US2006/0228415A1) [33]
150 mg ER (s.d.)	30	EBup	369.29	231.71	1.59	20.01	12.54	1.60	ta	Connarn 2017 [18]
300 mg ER (s.d.)	30	EBup	737.65	456.12	1.62	40.29	22.44	1.79	te	Connarn 2017 [18]
300 mg ER (m.d.)	30	EBup	2041.89	2144.43	0.95	95.75	103.90	0.92	te	Patent 1a (US2006/0228415A1) [33]
300 mg ER (m.d.)	49	EBup	1992.83	1807.20	1.10	96.76	89.23	1.08	ta	Patent 1b (US2006/0228415A1) [33]
300 mg ER (m.d.)	38	EBup	2050.67	2143.29	0.96	96.28	103.42	0.93	ta	Patent 3 (US2006/0228415A1) [45]
75 mg IR (s.d.)	7	TBup	777.19	644.82	1.21	42.87	47.04	0.91	te	Hesse 2006 [28]
75 mg IR (s.d.)	30	TBup	855.38	723.62	1.18	46.53	45.94	1.01	te	Connarn 2017 [18]
100 mg IR (s.d.)	32	TBup	1147.99	1072.29	1.07	60.66	70.73	0.86	ta	Connarn 2017 [18]
100 mg IR (s.d.)	15	TBup	2339.96	1220.23	1.92	54.62	46.64	1.17	te	Masters 2016 [29]
100 mg IR (s.d.)	8	TBup	1742.91	1681.44	1.04	55.59	69.63	0.80	te	Posner 1985a [32]
100 mg IR (m.d.)	8	TBup	14825.56	13696.52	1.08	438.38	458.40	0.96	te	Posner 1985b [32]
100 mg IR (s.d.)	8	TBup	2074.02	1548.84	1.34	64.15	59.95	1.07	te	Posner 1985 [32]
100 mg IR (m.d.)	30	TBup	9898.68	11793.82	0.84	454.37	579.14	0.78	te	Patent 1a (US2006/0228415A1) [33]
100 mg SR (s.d.)	30	TBup	1072.66	1097.26	0.98	54.17	70.09	0.77	te	Connarn 2017 [18]
150 mg SR (m.d.)	42	TBup	4781.47	4372.26	1.09	224.52	201.32	1.12	te	Benowitz 2013 [37]
150 mg SR (s.d.)	32	TBup	1623.87	1518.32	1.07	81.37	82.05	0.99	ta	Connarn 2017 [18]

Table S2.4: Predicted and observed AUC_{last} and C_{max} values of bupropion, hydroxybupropion, erythrohydrobupropion and threohydrobupropion plasma concentrations. (continued)

**Dosing**	**n**	**Compound**	**AUC_{last} pred [ng*h/ml]**	**AUC_{last} obs [ng*h/ml]**	**AUC_{last} pred/obs**	**C_{max} pred [ng/ml]**	**C_{max} obs [ng/ml]**	**C_{max} pred/obs**	**Dataset**	**Reference**
150 mg SR (m.d.)	49	TBup	10243.07	10905.15	0.94	466.39	539.88	0.86	ta	Patent 1b (US2006/0228415A1) [33]
150 mg ER (s.d.)	30	TBup	1507.34	1169.41	1.29	80.79	63.89	1.26	ta	Connarn 2017 [18]
300 mg ER (s.d.)	30	TBup	3000.24	2113.31	1.42	161.42	121.15	1.33	te	Connarn 2017 [18]
300 mg ER (m.d.)	30	TBup	10332.95	10585.97	0.98	488.69	560.01	0.87	te	Patent 1a (US2006/0228415A1) [33]
300 mg ER (m.d.)	49	TBup	10525.00	9316.26	1.13	495.81	486.37	1.02	ta	Patent 1b (US2006/0228415A1) [33]
300 mg ER (m.d.)	38	TBup	10784.29	10055.56	1.07	492.44	518.18	0.95	te	Patent 3 (US2006/0228415A1) [45]
**GMFE (range)**					**1.31 (1.00–3.06)**			**1.29 (1.00–2.87)**		
**pred/obs within twofold (range)**					**95.97%; 119/124 (0.43–3.06)**			**97.58%; 121/124 (0.55–2.87)**		

AUC_{last}, area under the concentration-time curve calculated from the first time point to the last time point; **Bup**, bupropion; **Cap**, capsule (Geneva cocktail [74]); **C_{max}**, maximum concentration; **EBup**, erythrohydrobupropion; **ER**, extended release tablet formulation; **GMFE**,geometric mean fold error; **HBup**, hydroxybupropion; **IR**, immediate release tablet formulation; **m.d.**, multiple dose; **n**, number of individuals studied; **obs**, observed; **pred** predicted; **s.d.**, single dose; **SR**, sustained release formulation; **TBup**, threohydrobupropion; **ta**, training dataset; **te**, test dataset; **-**, not available.

2.5.5 Local sensitivity analysis

Figures S2.5.19-S2.5.22 show the results of the local sensitivity analyses on the AUC of the compounds bupropion, hydroxybupropion, erythrohydrobupropion and threohydrobupopion. Sensitivity of the model to single parameter changes was determined as change of the simulated AUC extrapolated to infinity from the time of bupropion application after the last application of a 14 day multiple dose regimen of 100 mg IR (three times daily), 150 mg SR (twice daily) or 300 mg ER (once daily). A sensitivity value of -0.5 indicates a 5% decrease of the simulated AUC if the examined parameter is incraeased by 10%. Meaningful differences between formulations were only visible for the bupropion AUC. For all modeled compounds, fraction unbound in plasma (nonoptimized value) had the strongest impact. Lipophilicity of bupropion (optimized value) was more impactful for extended release administrations than for immediate release and sustained release formulations. In general, metabolite AUC is less sensitive to changes in bupropion lipophilicity than to metabolic pathways. Among the alteration of kinetics of the implemented metabolic pathways, CYP2B6 as well as 11β-HSD kinetics show a more profound influence on bupropion AUC than CYP2C19 kinetics, which reflects the key role of CYP2B6 in bupropion metabolism as described in literature [21]. Binding parameters K_D and k_{off} show no impact AUC of bupropion and metabolites after multiple dose application.

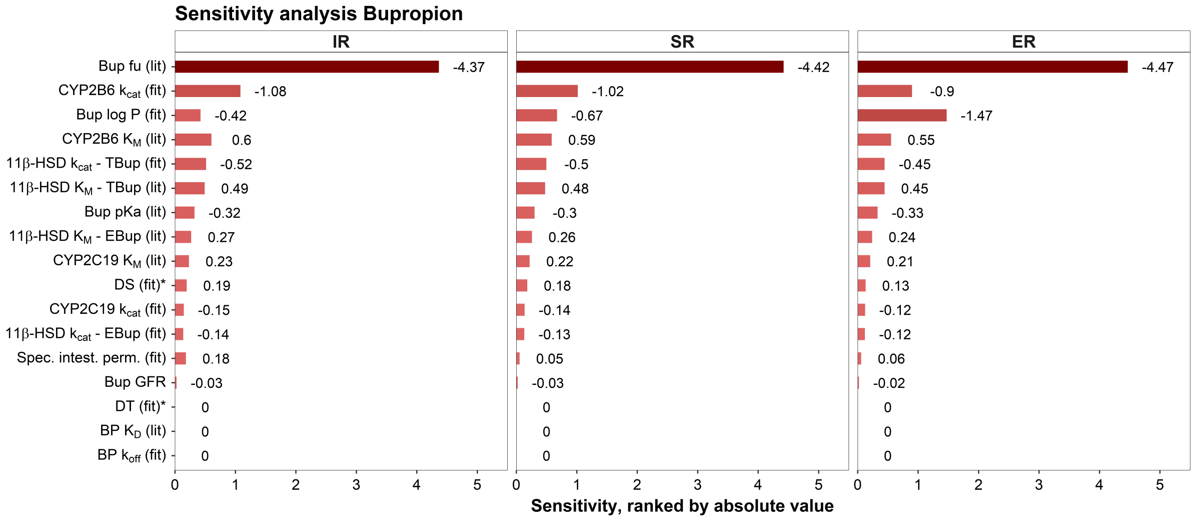

Figure S2.5.19: **Bupropion PBPK model sensitivity analyses - bupropion**. *, fitted for IR and SR; **11β-HSD**, 11β-hydroxysteroid dehydrogenase 1; **BP**, binding partner; **Bup**, bupropion; **CYP**, cytochrome P450; **DS**, dissolution shape; **DT**, dissolution time; **EBup**, erythrohydrobupropion; **ER**, extended release; **fit.**, fitted in parameter optimization; **GFR**, glomerular filtration rate; **IR**, immediate release; **K_D**, dissociation constant for binding; **k_{cat}**, catalytic rate constant; **K_M**, Michaelis-Menten constant; **k_{off}**, dissociation rate constant for binding; **lit.**, literature; **log P**, lipophilicity; **pKa**, acidic dissociation constant; **Spec. intest. perm.**, specific intestinal permeability; textbfSR, sustained release; **TBup**, threohydrobupropion.

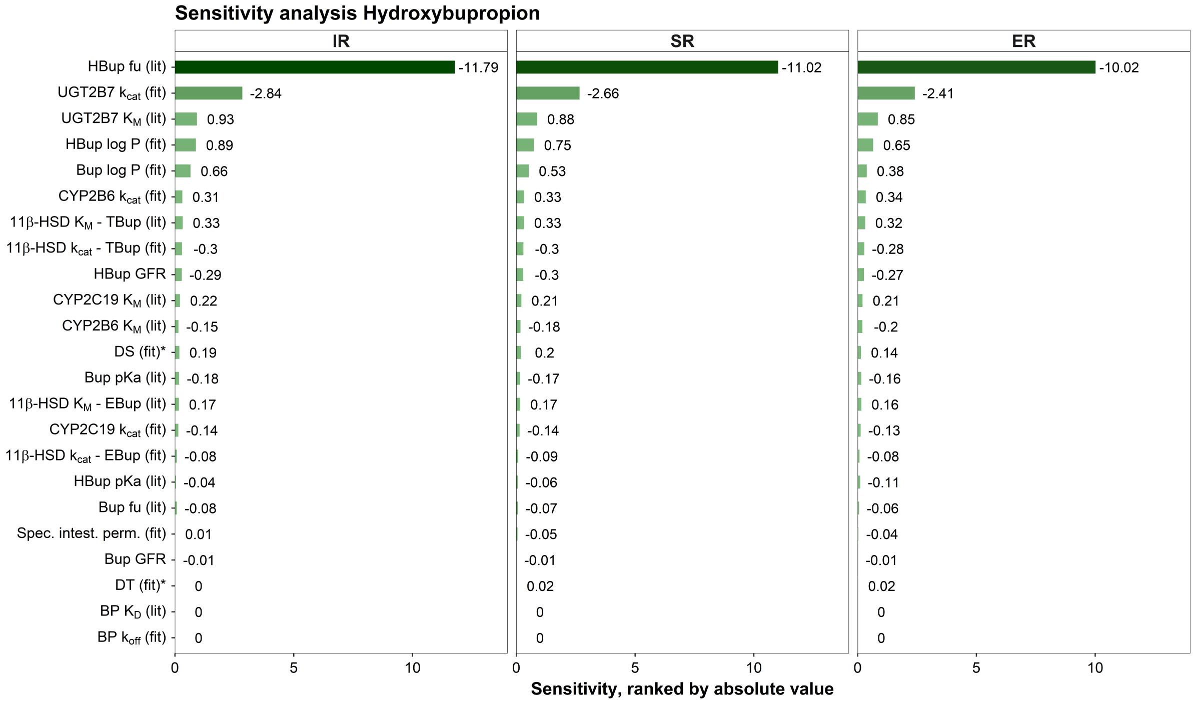

Figure S2.5.20: **Bupropion PBPK model sensitivity analyses - hydroxybupropion**. *, fitted for IR and SR; **11β-HSD**, 11β-hydroxysteroid dehydrogenase 1; **BP**, binding partner; **Bup**, bupropion; **CYP**, cytochrome P450; **DS**, dissolution shape; **DT**, dissolution time; **EBup**, erythrohydrobupropion; **ER**, extended release; **fit.**, fitted in parameter optimization; **GFR**, glomerular filtration rate; **HBup**, hydroxybupropion; **IR**, immediate release; $\mathbf{K_D}$, dissociation constant for binding; $\mathbf{k_{cat}}$, catalytic rate constant; $\mathbf{K_M}$, Michaelis-Menten constant; $\mathbf{k_{off}}$, dissociation rate constant for binding; **lit.**, literature; **log P**, lipophilicity; **pKa**, acidic dissociation constant; **Spec. intest. perm.**, specific intestinal permeability; textbfSR, sustained release; **TBup**, threohydrobupropion.

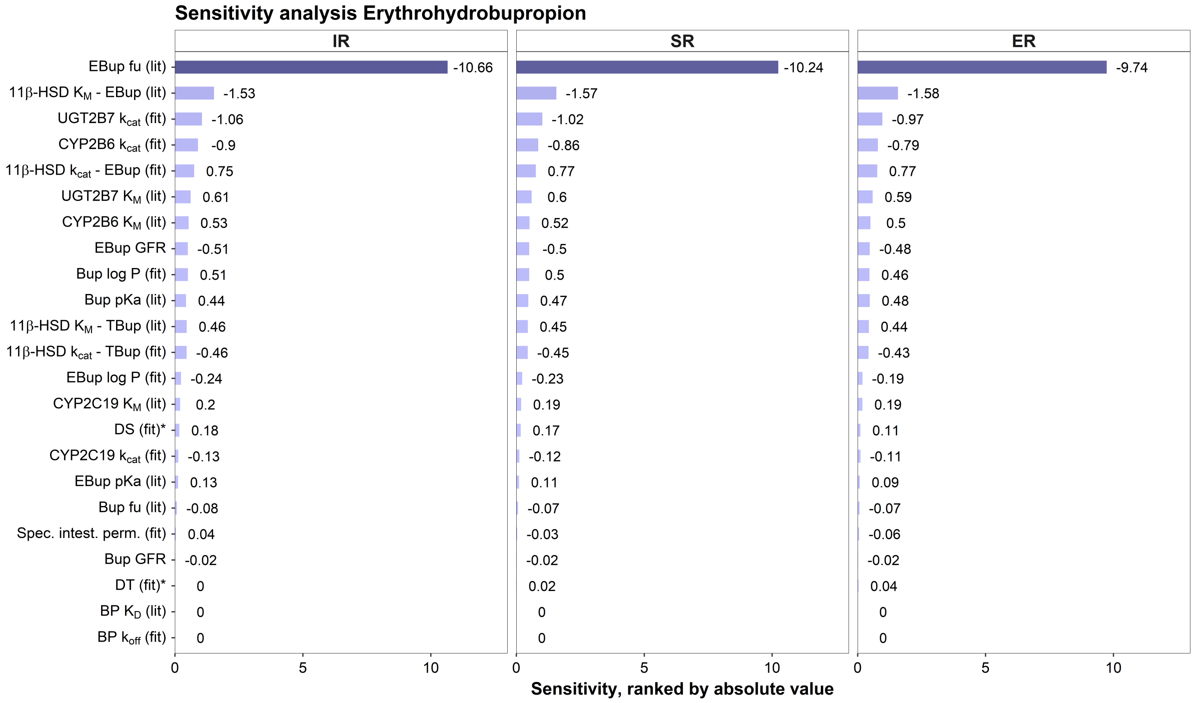

Figure S2.5.21: **Bupropion PBPK model sensitivity analyses - erythrohydrobupropion**. *, fitted for IR and SR; **11β-HSD**, 11β-hydroxysteroid dehydrogenase 1; **BP**, binding partner; **Bup**, bupropion; **CYP**, cytochrome P450; **DS**, dissolution shape; **DT**, dissolution time; **EBup**, erythrohydrobupropion; **ER**, extended release; **fit.**, fitted in parameter optimization; **GFR**, glomerular filtration rate; **IR**, immediate release; **K_D**, dissociation constant for binding; **k_{cat}**, catalytic rate constant; **K_M**, Michaelis-Menten constant; **k_{off}**, dissociation rate constant for binding; **lit.**, literature; **log P**, lipophilicity; **pKa**, acidic dissociation; **Spec. intest. perm.**, specific intestinal permeability; textbfSR, sustained release; **TBup**, threohydrobupropion.

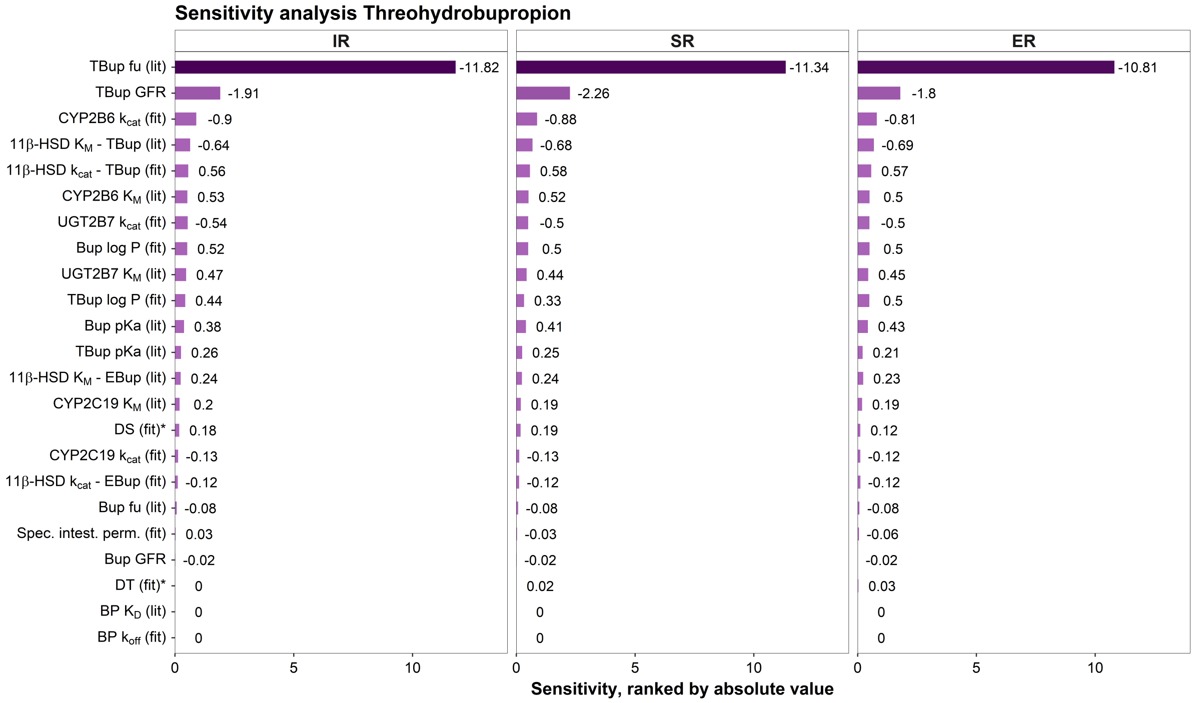

Figure S2.5.22: **Bupropion PBPK model sensitivity analyses - threohydrobupropion**. *, fitted for IR and SR; **11β-HSD**, 11β-hydroxysteroid dehydrogenase 1; **BP**, binding partner; **Bup**, bupropion; **CYP**, cytochrome P450; **DS**, dissolution shape; **DT**, dissolution time; **EBup**, erythrobupropion; **ER**, extended release; **fit.**, fitted in parameter optimization; **GFR**, glomerular filtration rate; **IR**, immediate release; **K_D**, dissociation constant for binding; **k_{cat}**, catalytic rate constant; **K_M**, Michaelis-Menten constant; **k_{off}**, dissociation rate constant for binding; **lit.**, literature; **log P**, lipophilicity; **pKa**, acidic dissociation; **Spec. intest. perm.**, specific intestinal permeability; textbfSR, sustained release; **TBup**, threohydrobupropion.

3 DGI prediction

3.1 Background

CYP2B6 expression can be influenced by polymorphisms, especially single nucleotide polymorphisms [21]. Several genetic variants for the gene encoding for CYP2B6 have been reported [75]. According to dose recommendations published by the clinical pharmacogenetics implementation consortium (CPIC), these can lead to different phenotypes, i.e. poor metabolizers (PM), intermediate metabolizers (IM), normal metabolizers (NM) and rapid metabolizers (RM) [75]. For bupropion, dose recommendation based on CYP2B6 genotype are not established yet. Genetic polymorphisms that were included in the model were: *CYP2B6*1*, *CYP2B6*4*, *CYP2B6*5* and *CYP2B6*6*. Various combinations as homo- or heterozygous expressions of these polymorphisms were simulated. Polymorphisms were chosen based on their frequency and functionality in order to describe various investigated phenotypes (*CYP2B6*1*: 49.07%, *CYP2B6*4*: 4.09%, *CYP2B6*5*: 11.55% and *CYP2B6*6*: 23.30% in European populations).
For the description of different allele combinations, the enzyme was integrated twice. For the variants *CYP2B6*4* and *CYP2B6*5*, necessary parameters (K_M and k_{cat}) were obtained from literature. Furthermore, K_M values for the *CYP2B6*1* and *CYP2B6*6* genotypes were also derived from the literature, whereas the k_{cat} value for *CYP2B6*1* was optimized with data on mostly wildtype subjects and the k_{cat} value for *CYP2B6*6* by fitting plasma concentration-time profiles to a study including only *CYP2B6*6/*6* subjects. Tabls S3.1 lists the clinical studies and Table S3.2 the model parameter used for DGI prediction. DGI $AUC_{HBup/Bup}$ and $C_{max\ HBup/Bup}$ ratios calculated from predictions were compared to observed values described in the literature.

3.2 Clinical studies

Clinical studies listed in Table S3.1 include data of patients with *CYP2B6* variants. Virtual individuals were built according to the demographics published in the respective study reports. If no data on demographics was reported, a standard individual were used as described in Section 1.4.

Table S3.1: Clinical studies used for DGI model development.

Dosing	n	Age [years]	Weight [kg]	BMI [kg/m²]	Females [%]	CYP2B6 genotype (n)	Dataset	Reference
150 mg IR (s.d.)	21	28 (±7)	72 (±14)	-	57	*1/*1	te	Kharasch 2019 [21]
150 mg IR (s.d.)	4	29 (±7)	68 (±14)	-	50	*1/*4 and *4/*6	te	Kharasch 2019 [21]
150 mg IR (s.d.)	20	28 (±8)	78 (±12)	-	30	*1/*6	te	Kharasch 2019 [21]
150 mg IR (s.d.)	2	28 (±1)	84 (±0)	-	0	*5/*5	te	Kharasch 2019 [21]
150 mg IR (s.d.)	17	32 (±9)	71 (±13)	-	59	*6/*6	ta	Kharasch 2019 [21]
150 mg SR (s.d.)	22	22.7	65	-	27.3	*1/*1 (19), *1/*4 (3)	ta	Chung 2011 [38]
150 mg SR (s.d.)	13	22.7	65	-	27.3	*1/*6 (11), *6/*6 (2)	te	Chung 2011 [38]
150 mg SR (s.d.)	19	-	-	-	-	*1/*1	te	[a]Gao 2016 [13]
150 mg SR (s.d.)	11	-	-	-	-	*1/*6	te	[a]Gao 2016 [13]
150 mg SR (s.d.)	6	-	-	-	-	*6/*6	te	[a]Gao 2016 [13]
150 mg SR (s.d.)	6	22 (19–34)	72 (53–99)	23.1 (18.4–26.9)	0	*1/*1	te	Loboz 2006 [44]
150 mg SR (s.d.)	1	22 (20–32)	64 (53–76)	21.4 (18.4–24.4)	0	*1/*4	te	Loboz 2006 [44]
150 mg SR (s.d.)	1	22 (19–34)	80 (60–99)	24.8 (19.7–26.9)	0	*1/*5	te	Loboz 2006 [44]
150 mg SR (s.d.)	6	22 (19–34)	72 (53–99)	23.1 (18.4–26.9)	0	*1/*6	te	Loboz 2006 [44]
150 mg SR (s.d.)	1	22 (19–34)	80 (60–99)	24.8 (19.7–26.9)	0	*5/*5	te	Loboz 2006 [44]
150 mg SR (s.d.)	1	22 (20–32)	64 (53–76)	21.4 (18.4–24.4)	0	*4/*6	te	Loboz 2006 [44]
150 mg SR (s.d.)	1	22 (20–32)	64 (53–76)	21.4 (18.4–24.4)	0	*6/*6	te	Loboz 2006 [44]

BMI, body mass index; **CYP**, cytochrome P450; **IR**, immediate release formulation; **n**, number of individuals studied; **s.d.**, single dose; **SR**, sustained release formulation; **ta**, training dataset; **te**, test dataset; **-**, no data available. Values are given as mean ± standard deviation (SD), the range of values is given in brackets.
[a], reported as SR but simulated as IR due to early C_{max} vales observed.

3.3 Drug-dependent model parameters

For implementation of the DGIs, model parameters were used as for the general model listed previously in Table S2.2. Table S3.2 lists the parameters that were adapted for different genotype scenarios. Details on DGI implementation can be found in Section 1.5.1.

Table S3.2: Model parameter adapted for DGI implementation.

Parameter	Unit	Value	Source	Reference	Description
CYP2B6 K_M **1*	µmol/l	25.80	lit.	[66]	Michaelis-Menten constant for the **1* allele
CYP2B6 k_{cat} **1*	1/min	10.87	opt.	[66]	Catalytic rate constant for the **1* allele
CYP2B6 K_M **6*	µmol/l	61.62	lit.	[66]	Michaelis-Menten constant for the **6* allele
CYP2B6 k_{cat} **6*	1/min	9.52	opt.	[66]	Catalytic rate constant for the **6* allele
CYP2B6 K_M **4*	µmol/l	12.70	lit.	[76]	Michaelis-Menten constant for the **4* allele
CYP2B6 k_{cat} **4*	1/min	18.12	lit.	[76]	Catalytic rate constant for the **4* allele
CYP2B6 K_M **5*	µmol/l	15.59	lit.	[76]	Michaelis-Menten constant for the **5* allele
CYP2B6 k_{cat} **5*	1/min	11.28	lit.	[76]	Catalytic rate constant for the **5* allele

CYP, cytochrome P450; **lit.**, literature; **opt.**, optimized.

3.4 Concentration-time profiles

The geometric means of the population predictions (n=500) are shown as solid lines and corresponding observed data as filled dots. Symbols represent the arithmetic mean values ± standard deviation, if available. The shaded areas indicate the geometric standard deviation. Details on dosing regimens, study populations and literature references are listed in Table S3.1.

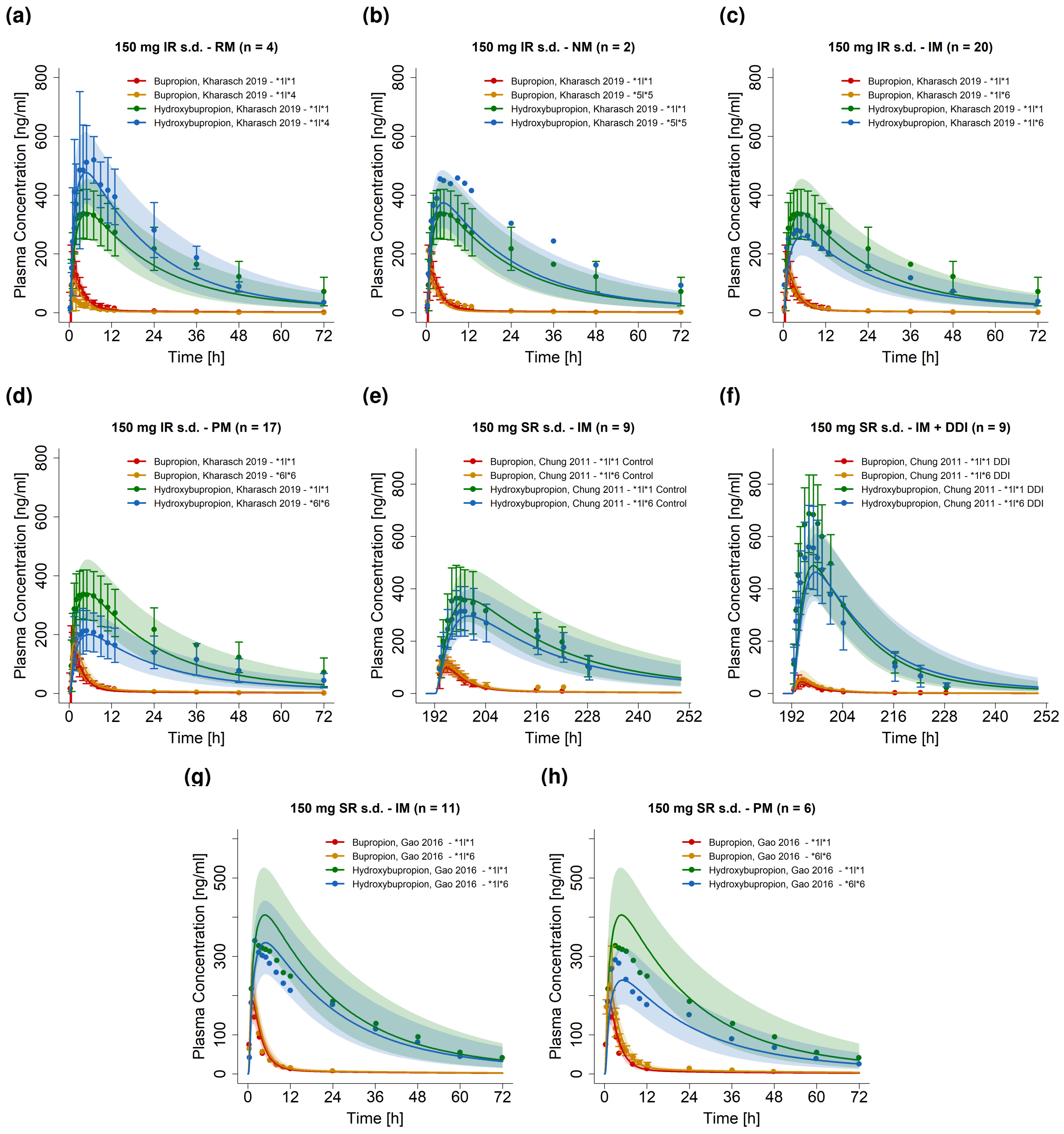

Figure S3.4.1: **Plasma concentration-time profiles of DGI predictions** on a linear scale. **Control**, without perpetrator; **DDI**, drug-drug-interaction with rifampicin; **IM**, intermediate metabolizers; **IR**, immediate release tablet; **NM**, normal metabolizers; **PM**, poor metabolizers; **RM**, rapid metabolizers; **s.d.**, single dose; **SR**, sustained release.

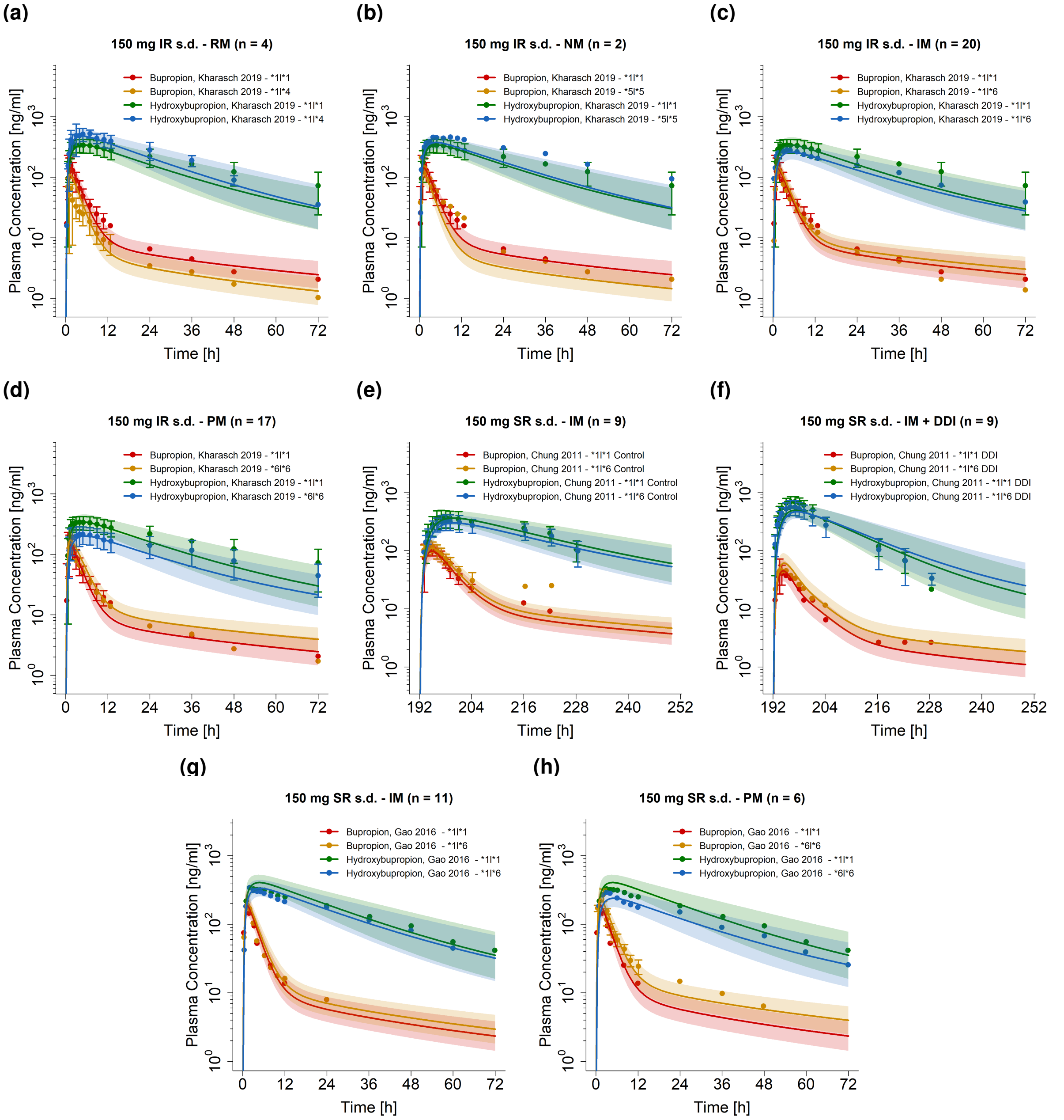

Figure S3.4.2: **Plasma concentration-time profiles of DGI predictions** on a semi-logarithmic scale. **Control**, without perpetrator; **DDI**, drug-drug-interaction with rifampicin; **IM**, intermediate metabolizers; **IR**, immediate release tablet; **NM**, normal metabolizers; **PM**, poor metabolizers; **RM**, rapid metabolizers; **s.d.**, single dose; **SR**, sustained release.

3.5 Model evaluation

3.5.1 Predicted compared to observed concentrations goodness-of-fit plots

Following, goodness-of-fit plots of predicted compared to observed plasma concentrations are illustrated in Figure S3.5.3. Details on dosing regimens, study populations and literature references are listed in Table S3.1. Predicted and observed PK parameters are summarized in Table S3.4.

(a) Bupropion **(b) Hydroxybupropion**

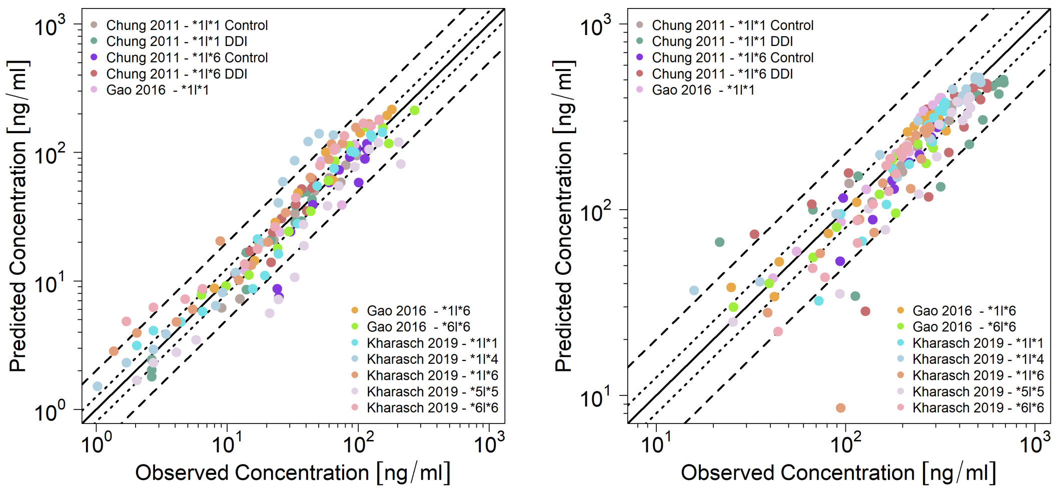

Figure S3.5.3: **DGI predicted compared to observed plasma concentrations of (a) bupropion and (b) hydroxybupropion.** The solid line marks the line of identity. Dotted lines indicate 1.25-fold, dashed lines indicate 2-fold deviation. **Control**, without perpetrator; **DDI**, drug-drug-interaction with perpetrator; **DGI**, drug-gene-interaction.

3.5.2 Mean relative deviation of plasma concentration predictions

Table S3.3: Mean relative deviation values of bupropion and hydroxybupropion DGI plasma concentration predictions.

Dosing	n	Compound	MRD	Compound	MRD	Dataset	Reference
150 mg IR (s.d.)	21	Bup	1.34	HBup	1.16	te	Kharasch 2019 **1/*1* [21]
150 mg IR (s.d.)	4	Bup	1.63	HBup	1.29	te	Kharasch 2019 **1/*4* & **4/*6* [21]
150 mg IR (s.d.)	20	Bup	1.86	HBup	1.48	te	Kharasch 2019 **1/*6* [21]
150 mg IR (s.d.)	2	Bup	1.58	HBup	1.17	te	Kharasch 2019 **5/*5* [21]
150 mg IR (s.d.)	17	Bup	1.91	HBup	1.16	ta	Kharasch 2019 **6/*6* [21]
150 mg SR (s.d.)	13	Bup	1.14	HBup	1.10	ta	Chung 2011 **1/*1* Control [38]
150 mg SR (s.d.)	13	Bup	1.47	HBup	1.42	ta	Chung 2011 **1/*1* DDI [38]
150 mg SR (s.d.)	9	Bup	1.28	HBup	1.11	te	Chung 2011 **1/*6* Control [38]
150 mg SR (s.d.)	9	Bup	1.37	HBup	1.39	te	Chung 2011 **1/*6* DDI [38]
150 mg SR (s.d.)	19	Bup	1.33	HBup	1.17	te	Gao 2016 **1/*1* [13]
150 mg SR (s.d.)	11	Bup	1.43	HBup	1.18	te	Gao 2016 **1/*6* [13]
150 mg SR (s.d.)	6	Bup	1.28	HBup	1.15	te	Gao 2016 **6/*6* [13]
		**Mean**	**1.33 (1.10–1.91)**				
		**Median**	**1.29 (1.10–1.91)**				
			**100% (24/24) $\leq$ 2**				

Bup, bupropion; **Control**, without perpetrator; **DDI**, drug-drug-interaction with perpetrator; **HBup**, hydroxybupropion; **IR**, immediate release formulation; **m.d.**, multiple dose; **MRD**, mean relative deviation; **n**, number of individuals studied; **s.d.**, single dose; **SR**, sustained release formulation; **ta**, training dataset; **te**, test dataset.

3.5.3 AUC and C_{max} goodness-of-fit plots

Following, predicted compared to observed AUC and C_{max} values are shown for individual bupropion and hydroxybupropion profiles, their metabolite-parent AUC and C_{max} ratio and their DGI effect metabolite-parent AUC and C_{max} ratio, respectively. Details on dosing regimens, study populations and literature references are listed in Table S3.1. Predicted and observed PK parameters are summarized in Tables S3.4, S3.5 and S3.6.

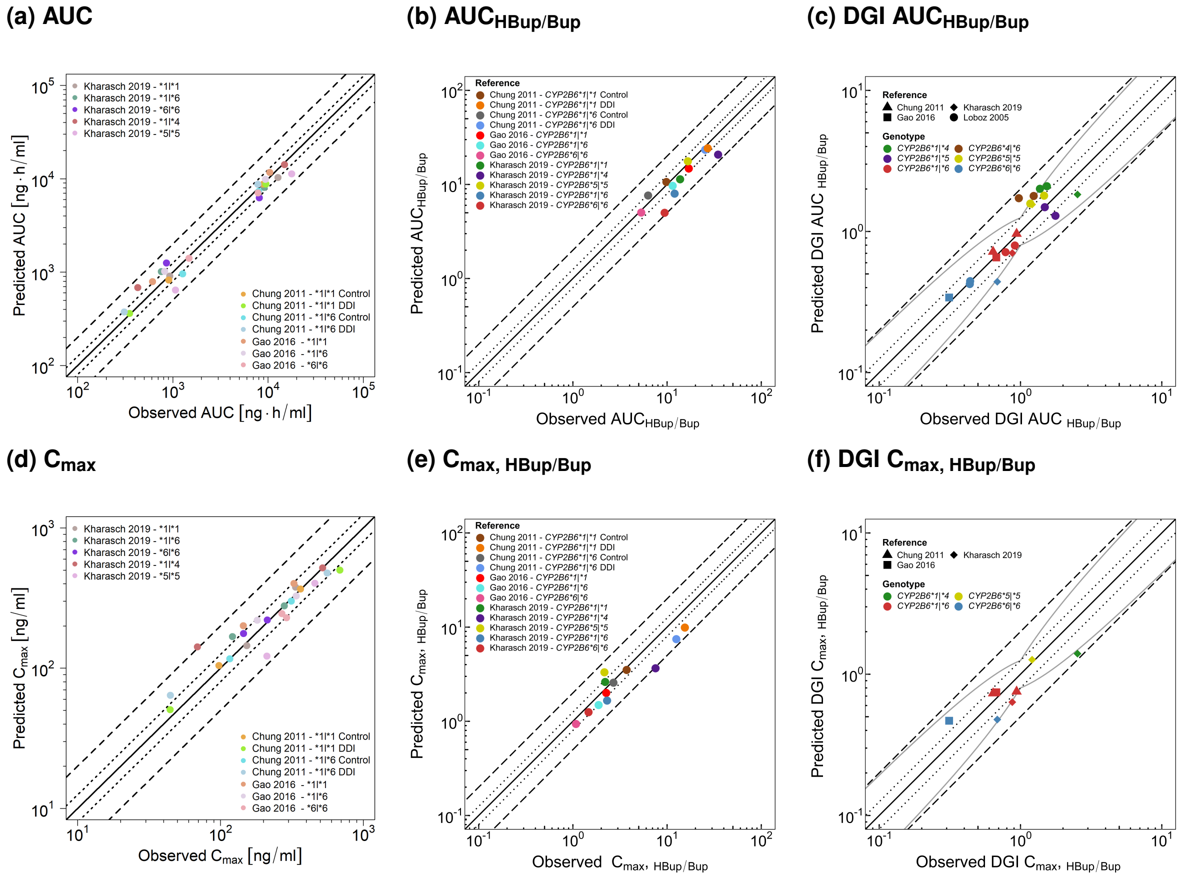

Figure S3.5.4: **Predicted compared to observed (a) AUC values, (b) $AUC_{HBup/Bup}$ ratios, (c) DGI $AUC_{HBup/Bup}$, (d) C_{max} values, (e) $C_{max, HBup/Bup}$ ratios and (f) DGI $C_{max, HBup/Bup}$ ratios.** The solid line marks the line of identity. Dotted lines indicate 1.25-fold, dashed lines indicate 2-fold deviation. The curved gray lines show the prediction success limits suggested by Guest et al. allowing a 1.25-fold variability [77]. **AUC**, area under the plasma concentration-time curve; **Bup**, bupropion; **C_{max}**, maximum plasma concentration; **Control**, without perpetrator; **DDI**, drug-drug-interaction with perpetrator; **DGI**, drug-gene-interaction; **HBup**, hydroxybupropion.

3.5.4 Geometric mean fold error of predicted AUC and C_{max} values, $AUC_{HBup/Bup}$ and $C_{max, HBup/Bup}$ ratios, and DGI $AUC_{HBup/Bup}$ and DGI $C_{max, HBup/Bup}$ ratios

Table S3.4: Predicted and observed AUC_{last} and C_{max} values of bupropion and hydroxybupropion DGI plasma concentrations.

Dosing	n	Compound	AUC_{last} pred [ng*h/ml]	AUC_{last} obs [ng*h/ml]	AUC_{last} pred/obs	C_{max} pred [ng/ml]	C_{max} obs [ng/ml]	C_{max} pred/obs	Dataset	Reference
150 mg IR (s.d.)	21	Bup	907.72	928.90	0.98	144.55	153.01	0.94	te	Kharasch 2019 *1/*1 [21]
150 mg IR (s.d.)	4	Bup	683.07	429.31	1.59	141.71	68.99	2.05	te	Kharasch 2019 *1/*4 & *4/*6 [21]
150 mg IR (s.d.)	20	Bup	1014.16	759.08	1.34	167.69	121.58	1.38	te	Kharasch 2019 *1/*6 [21]
150 mg IR (s.d.)	2	Bup	640.82	1070.57	0.60	121.60	212.09	0.57	te	Kharasch 2019 *5/*5 [21]
150 mg IR (s.d.)	17	Bup	1251.29	860.49	1.45	176.61	145.49	1.21	ta	Kharasch 2019 *6/*6 [21]
150 mg SR (s.d.)	13	Bup	823.49	909.01	0.91	104.29	97.73	1.07	ta	Chung 2011 *1/*1 Control [38]
150 mg SR (s.d.)	13	Bup	360.82	351.52	1.03	50.49	44.49	1.13	ta	Chung 2011 *1/*1 DDI [38]
150 mg SR (s.d.)	9	Bup	958.22	1268.88	0.76	116.76	116.63	1.00	te	Chung 2011 *1/*6 Control [38]
150 mg SR (s.d.)	9	Bup	373.58	309.15	1.21	63.99	44.66	1.43	te	Chung 2011 *1/*6 DDI [38]
150 mg SR (s.d.)	19	Bup	790.62	613.15	1.29	200.62	144.96	1.38	te	Gao 2016 *1/*1 [13]
150 mg SR (s.d.)	11	Bup	1016.99	820.34	1.24	219.70	181.33	1.21	te	Gao 2016 *1/*6 [13]
150 mg SR (s.d.)	6	Bup	1402.08	1483.80	0.94	244.27	269.84	0.91	te	Gao 2016 *6/*6 [13]
150 mg IR (s.d.)	28	HBup	10298.60	12764.00	0.81	378.25	336.77	1.12	te	Kharasch 2019 *1/*1 [21]
150 mg IR (s.d.)	4	HBup	14169.45	14938.93	0.95	518.15	520.08	1.00	te	Kharasch 2019 *1/*4 and *4/*6 [21]
150 mg IR (s.d.)	20	HBup	8093.83	9126.07	0.89	278.28	280.29	0.99	te	Kharasch 2019 *1/*6 [21]
150 mg IR (s.d.)	2	HBup	11293.90	17774.72	0.64	402.75	458.17	0.88	te	Kharasch 2019 *5/*5 [21]
150 mg IR (s.d.)	17	HBup	6250.18	8101.38	0.77	220.67	213.17	1.04	ta	Kharasch 2019 *6/*6 [21]
150 mg SR (s.d.)	13	HBup	8744.70	8936.26	0.98	366.16	363.64	1.01	ta	Chung 2011 *1/*1 Control [38]
150 mg SR (s.d.)	13	HBup	8750.56	9498.00	0.92	499.27	687.07	0.73	ta	Chung 2011 *1/*1 DDI [38]
150 mg SR (s.d.)	9	HBup	7304.46	7976.41	0.92	300.13	314.03	0.96	te	Chung 2011 *1/*6 Control [38]
150 mg SR (s.d.)	9	HBup	8726.17	7838.55	1.11	476.89	559.91	0.85	te	Chung 2011 *1/*6 DDI [38]
150 mg SR (s.d.)	19	HBup	11675.08	10421.81	1.12	401.09	327.24	1.23	te	Gao 2016 *1/*1 [13]
150 mg SR (s.d.)	11	HBup	9827.62	9404.32	1.05	326.93	340.08	0.96	te	Gao 2016 *1/*6 [13]
150 mg SR (s.d.)	6	HBup	7045.71	7886.01	0.89	228.64	291.04	0.79	te	Gao 2016 *6/*6 [13]
**GMFE (range)**					**1.22 (1.02–1.59)**			**1.21 (1.00–2.05)**		
**pred/obs within twofold (range)**					**100%; 24/24 (0.60–1.59)**			**95.83%; 23/24 (0.57–2.05)**		

AUC, area under the plasma concentration-time curve; **Bup**, bupropion; C_{max}, maximum plasma concentration; **Control**, without perpetrator; **DDI**, drug-drug-interaction with perpetrator; **GMFE**, geometric mean fold error; **HBup**, hydroxybupropion; **IR**, immediate release formulation; **n**, number of individuals studied; **obs**, observed; **pred**, predicted; **s.d.**, single dose; **SR**, sustained release formulation; **ta**, training dataset; **te**, test dataset.

Table S3.5: Predicted and observed $AUC_{HBup/Bup}$ and $C_{max, HBup/Bup}$ ratios of bupropion and hydroxybupropion DGI plasma concentrations.

**Dosing**	**n**	**$AUC_{HBup/Bup}$ pred**	**$AUC_{HBup/Bup}$ obs**	**$AUC_{HBup/Bup}$ pred/obs**	**$C_{max, HBup/Bup}$ pred**	**$C_{max, HBup/Bup}$ obs**	**$C_{max, HBup/Bup}$ pred/obs**	**Dataset**	**Reference**
150 mg IR (s.d.)	28	11.35	13.74	0.83	2.62	2.20	1.19	te	Kharasch 2019 *1/*1 [21]
150 mg IR (s.d.)	4	20.74	34.80	0.60	3.66	7.54	0.49	te	Kharasch 2019 *1/*4 and *4/*6 [21]
150 mg IR (s.d.)	20	7.98	12.02	0.66	1.66	2.31	0.72	te	Kharasch 2019 *1/*6 [21]
150 mg IR (s.d.)	2	17.62	16.60	1.06	3.31	2.16	1.53	te	Kharasch 2019 *5/*5 [21]
150 mg IR (s.d.)	17	4.99	9.41	0.53	1.25	1.47	0.85	ta	Kharasch 2019 *6/*6 [21]
150 mg SR (s.d.)	13	10.62	9.83	1.08	3.51	3.72	0.94	ta	Chung 2011 *1/*1 Control [38]
150 mg SR (s.d.)	13	24.25	27.02	0.90	9.89	15.44	0.64	ta	Chung 2011 *1/*1 DDI [38]
150 mg SR (s.d.)	9	7.62	6.29	1.21	2.57	2.69	0.95	te	Chung 2011 *1/*6 Control [38]
150 mg SR (s.d.)	9	23.36	25.36	0.92	7.45	12.54	0.59	te	Chung 2011 *1/*6 DDI [38]
150 mg SR (s.d.)	19	14.77	17.00	0.87	2.00	2.26	0.89	te	Gao 2016 *1/*1 [13]
150 mg SR (s.d.)	11	9.66	11.46	0.84	1.49	1.88	0.79	te	Gao 2016 *1/*6 [13]
150 mg SR (s.d.)	6	5.03	5.31	0.95	0.94	1.08	0.87	te	Gao 2016 *6/*6 [13]
150 mg SR (s.d.)	6	11.76	18.50	0.64	-	-	-	te	Loboz 2006 *1/*1 Control [44]
150 mg SR (s.d.)	6	21.78	30.90	0.70	-	-	-	te	Loboz 2006 *1/*1 DDI [44]
150 mg SR (s.d.)	1	22.75	25.40	0.90	-	-	-	te	Loboz 2006 *1/*4 Control [44]
150 mg SR (s.d.)	1	45.56	47.50	0.96	-	-	-	te	Loboz 2006 *1/*4 DDI [44]
150 mg SR (s.d.)	1	15.47	32.70	0.47	-	-	-	te	Loboz 2006 *1/*5 Control [44]
150 mg SR (s.d.)	1	32.36	45.90	0.71	-	-	-	te	Loboz 2006 *1/*5 DDI [44]
150 mg SR (s.d.)	6	8.38	14.50	0.58	-	-	-	te	Loboz 2006 *1/*6 Control [44]
150 mg SR (s.d.)	6	17.32	28.20	0.61	-	-	-	te	Loboz 2006 *1/*6 DDI [44]
150 mg SR (s.d.)	1	19.58	18.00	1.09	-	-	-	te	Loboz 2006 *4/*6 Control [44]
150 mg SR (s.d.)	1	38.87	38.30	1.01	-	-	-	te	Loboz 2006 *4/*6 DDI [44]
150 mg SR (s.d.)	1	18.87	21.70	0.87	-	-	-	te	Loboz 2006 *5/*5 Control [44]
150 mg SR (s.d.)	1	39.06	45.40	0.86	-	-	-	te	Loboz 2006 *5/*5 DDI [44]
150 mg SR (s.d.)	1	4.98	8.10	0.61	-	-	-	te	Loboz 2006 *6/*6 Control [44]
150 mg SR (s.d.)	1	9.69	13.60	0.71	-	-	-	te	Loboz 2006 *6/*6 DDI [44]
**GMFE (range)**				**1.33 (1.01–2.14)**			**1.31 (1.04–2.06)**		
**pred/obs within twofold (range)**				**96.15%; 25/26 (0.47–1.21)**			**91.67%; 11/12 (0.49–1.53)**		

AUC, area under the plasma concentration-time curve; **Bup**, bupropion; **C_{max}**, maximum plasma concentration; **Control**, without perpetrator; **DDI**, drug-drug-interaction with perpetrator; **GMFE**, geometric mean fold error; **HBup**, hydroxybupropion; **IR**, immediate release formulation; **n**, number of individuals studied; **obs**, observed; **pred**, predicted; **s.d.**, single dose; **SR**, sustained release formulation; **ta**, training dataset; **te**, test dataset; **-**, no data available.

Table S3.6: Predicted and observed DGI $AUC_{HBup/Bup}$ and DGI $C_{max,\,HBup/Bup}$ ratios of bupropion and hydroxybupropion DGI plasma concentrations.

Dosing	n	DGI $AUC_{HBup/Bup}$ pred	DGI $AUC_{HBup/Bup}$ obs	DGI $AUC_{HBup/Bup}$ pred/obs	DGI $C_{max,\,HBup/Bup}$ pred	DGI $C_{max,\,HBup/Bup}$ obs	DGI $C_{max,\,HBup/Bup}$ pred/obs	Dataset	Reference
150 mg IR (s.d.)	4	1.83	2.53	0.72	1.40	3.43	0.41	te	Kharasch 2019 **1/*4* and **4/*6* [21]
150 mg IR (s.d.)	20	0.70	0.87	0.80	0.63	1.05	0.60	te	Kharasch 2019 **1/*6* [21]
150 mg IR (s.d.)	2	1.55	1.21	1.28	1.26	0.98	1.29	te	Kharasch 2019 **5/*5* [21]
150 mg IR (s.d.)	17	0.44	0.68	0.64	0.48	0.67	0.71	ta	Kharasch 2019 **6/*6* [21]
150 mg SR (s.d.)	9	0.72	0.64	1.13	0.73	0.64	1.12	te	Chung 2011 **1/*6* Control [38]
150 mg SR (s.d.)	9	0.96	0.94	1.02	0.75	0.94	1.02	te	Chung 2011 **1/*6* DDI [38]
150 mg SR (s.d.)	11	0.65	0.67	0.97	0.75	0.83	0.90	te	Gao 2016 **1/*6* [13]
150 mg SR (s.d.)	6	0.34	0.31	1.09	0.47	0.48	0.98	te	Gao 2016 **6/*6* [13]
150 mg SR (s.d.)	1	2.01	1.37	1.46	-	-	-	te	Loboz 2006 **1/*4* Control [44]
150 mg SR (s.d.)	1	2.09	1.54	1.36	-	-	-	te	Loboz 2006 **1/*4* DDI [44]
150 mg SR (s.d.)	1	1.29	1.77	0.73	-	-	-	te	Loboz 2006 **1/*5* Control [44]
150 mg SR (s.d.)	1	1.49	1.49	1.00	-	-	-	te	Loboz 2006 **1/*5* DDI [44]
150 mg SR (s.d.)	6	0.71	0.78	0.91	-	-	-	te	Loboz 2006 **1/*6* Control [44]
150 mg SR (s.d.)	6	0.80	0.91	0.87	-	-	-	te	Loboz 2006 **1/*6* DDI [44]
150 mg SR (s.d.)	1	1.72	0.97	1.77	-	-	-	te	Loboz 2006 **4/*6* Control [44]
150 mg SR (s.d.)	1	1.78	1.24	1.44	-	-	-	te	Loboz 2006 **4/*6* DDI [44]
150 mg SR (s.d.)	1	1.58	1.17	1.34	-	-	-	te	Loboz 2006 **5/*5* Control [44]
150 mg SR (s.d.)	1	1.79	1.47	1.22	-	-	-	te	Loboz 2006 **5/*5* DDI [44]
150 mg SR (s.d.)	1	0.42	0.44	0.97	-	-	-	te	Loboz 2006 **6/*6* Control [44]
150 mg SR (s.d.)	1	0.44	0.44	1.01	-	-	-	te	Loboz 2006 **6/*6* DDI [44]
**GMFE (range)**				**1.25 (1.00–1.77)**			**1.35 (1.05–2.44)**		
**pred/obs within twofold (range)**				**100%; 20/20 (0.64–1.77)**			**87.5%; 7/8 (0.41–1.29)**		

AUC, area under the plasma concentration-time curve; **Bup**, bupropion; $\mathbf{C_{max}}$, maximum plasma concentration; **Control**, without perpetrator; **DDI**, drug-drug-interaction with perpetrator; **GMFE**, geometric mean fold error; **HBup**, hydroxybupropion; **IR**, immediate release formulation; **n**, number of individuals studied; **obs**, observed; **pred**, predicted; **s.d.**, single dose; **SR**, sustained release formulation; **ta**, training dataset; **te**, test dataset; **-**, no data available.

4 DDI prediction

4.1 PBPK modeling of rifampicin

The antibiotic rifampicin exhibits a strong DDI potential. As an agonist of the pregnane X receptor, rifampicin induces multiple metabolizing enzymes, i.e. CYPs (CYP2B6 or CYP2C19) and UGTs (UGT2B7) [38]. The rifampicin PBPK model was developed and applied for several DDI predictions in previous publications [78–80]. For prediction of rifampicin-bupropion DDIs, interaction parameters were gathered from literature for implementation of rifampicins' influence on the following enzymes: CYP2B6, CYP2C19 and UGT2B7. Drug-dependent parameters of the rifampicin model are listed in Table S4.1.

Table S4.1: Drug-dependent parameters of the rifampicin PBPK model.

Parameter	Unit	Value	Source	Reference	Description
MW	g/mol	822.94	lit.	[a]DB01045	Molecular weight
pKa (acid)	-	1.70	lit.	[81]	Acid dissociation constant
pKa (base)	-	7.90	lit.	[81]	Acid dissociation constant
Solubility (pH)	g/l	2.80 (7.5)	lit.	[82–85]	Solubility
logP	-	[b]2.50	1.30, 2.70	[82, 86]	Lipophilicity
fu	%	17.0	lit.	[87]	Fraction unbound
B/P ratio	-	0.89	[c,d]0.89	[88]	Blood/plasma ratio
Specific intest. perm.	cm/min	[b]1.24E-05	[d]3.84E-07	PK-Sim®	Normalized to surface area
Organ perm.	cm/min	2.93E-05	[d]2.93E-05.	PK-Sim®	Normalized to surface are
GFR fraction	-	1.00	asm.	-	Fraction of filtered drug in the urine
EHC cont. fraction	-	1.00	asm.	-	Fraction of bile continually released
Cellular permeabilities	cm/min	PK-Sim std.	-	[1]	Permeation across cell membranes
Partition coefficients	-	R&R	-	[89, 90]	Organ-plasma partition coefficients
AADAC K_M	µmol/l	195.10	lit.	[91]	AADAC Michaelis-Menten constant
AADAC kcat	1/min	[b]9.87	-	-	AADAC catalytic rate constant
OATP1B1 K_M	µmol/l	1.50	lit.	[92]	OATP1B1 Michaelis-Menten constant
OATP1B1 kcat	1/min	[b]105.41	-	-	OATP1B1 catalytic rate constant
Pgp K_M	µmol/l	55.0	lit.	[93]	Pgp Michaelis-Menten constant
Pgp kcat	1/min	[b]0.61	-	-	Pgp catalytic rate constant
Induction EC_{50}	µmol/l	0.34	lit.	[87, 94]	Conc. for half-maximal induction
AADAC E_{max}	-	[b]0.99	-	-	Maximum in vivo induction effect
OATP1B1 E_{max}	-	[b]0.38	-	-	Maximum in vivo induction effect
Pgp E_{max}	-	2.50	lit.	[95]	Maximum in vivo induction effect
CYP2B6 E_{max}	-	3.60	lit.	[96]	Maximum in vivo induction effect
CYP2C19 E_{max}	-	1.07	lit.	[97]	Maximum in vivo induction effect
UGT2B7 E_{max}	-	1.79	lit.	[98]	Maximum in vivo induction effect
OATP1B1 K_i	µmol/l	0.48	lit.	[99]	Conc. for half-maximal inhibition
Pgp K_i	µmol/l	169.00	lit.	[100]	Conc. for half-maximal inhibition
CYP2B6 K_i	µmol/l	[e]118.5	lit.	[101]	Conc. for half-maximal inhibition
UGT2B7 K_i	µmol/l	[e]554.0	lit.	[102]	Conc. for half-maximal inhibition
Formulation		Solution			

asm., assumed; **conc.**, concentration; **cont.**, continuous; **CYP**, cytochrome P450; **EHC**, enterohepatic circulation; **intest.**, intestinal; **GFR**, glomerular filtration rate; **lit.**, literature; **OATP**, organic anion transporting polypeptide; **perm.**, permeability; **Pgp**, P-glycoprotein; **PK-Sim std.**, PK-Sim Standard calculation method; **R&R**, Rodgers and Rowland calculation method; **UGT**, uridine 5'-diphospho-glucuronosyltransferase; **-**, not available.

[a], https://www.drugbank.ca/drugs/DB01045, 22 October 2018

[b], optimized

[c], blood/serum concentration ratio

[d], calculated parameter

[e], in vitro values corrected for binding in the assay using fraction unbound to microsomal protein [72]

4.2 PBPK modeling of fluvoxamine

The selective serotonin reuptake inhibitor fluvoxamine exhibits a strong inhibitory activity on several CYP enzymes, especially on CYP1A2, CYP2C19 and CYP3A4 [103, 104]. The fluvoxamine PBPK model was developed and applied for DDI predictions in a previous publication [103]. To describe a DDI cocktail study conducted by Bosilkovska et al. [25], the fluvoxamine model was used to predict the reported fluvoxamine-voriconazole-bupropion DDI, by implementing competitive inhibition on CYP2C19 and CYP3A4 using interaction parameters from the literature. Drug-dependent parameters of the fluvoxamine model are listed in Table S4.2.

Table S4.2: Drug-dependent parameters of the fluvoxamine PBPK model.

Parameter	Unit	Value	Source	Reference	Description
MW	g/mol	318.34	lit.	[a]DB00176	Molecular weight
pKa (base)	-	9.40	lit.	[105]	Acid dissociation constant
Solubility (pH)	mg/ml	14.66 (7.0)	lit.	MSDS	Solubility
logP	-	[b]3.57	3.20	[a]DB00176	Lipophilicity
fu	%	23	lit.	[106]	Fraction unbound
Specific intest. perm.	dm/min	[b]2.74E-06	[c]3.03E-5	PK-Sim®	Normalized to surface are
Specific organ perm.	dm/min	[c]0.02	[c]0.02	PK-Sim®	Normalized to surface are
GFR fraction	-	1.00	asm.	-	Fraction of filtered drug in the urine
EHC cont. fraction	-	1.00	asm.	-	Fraction of bile continually released
Cellular permeabilities	cm/min	PK-Sim std.	-	[1]	Permeation across cell membranes
Partition coefficients	cm/min	Schmitt	-	[107]	Organ-plasma partition coefficients
CYP1A2 K_M	µmol/l	[b]0.0074	-	-	CYP1A2 Michaelis-Menten constant
CYP1A2 kcat	1/min	[b]0.016	opt.	-	CYP1A2 catalytic rate constant
CYP2D6 K_M	µmol/l	76.30	lit.	[108]	CYP2D6 Michaelis-Menten constant
CYP2D6 kcat	1/min	[b]110.51	-	-	CYP2D6 catalytic rate constant
CYP2C19 K_i	µmol/l	0.013	lit.	[104]	Conc. for half-maximal inhibition
CYP3A4 K_i	µmol/l	1.60	lit.	**[? ?]**	Conc. for half-maximal inhibition
Formulation		Solution			

asm., assumed; **calc.**, calculated; **conc.**, concentration; **cont.**, continuous; **CYP**, cytochrome P450; **EHC**, enterohepatic circulation; **intest.**, intestinal; **GFR**, glomerular filtration rate; **lit.**, literature; **MSDS**, material safety data sheet; **perm.**, permeability; **PK-Sim std.**, PK-Sim Standard calculation method; **Schmitt**, Schmitt calculation method; **-**, not available.
[a], https://www.drugbank.ca/drugs/DB00176, 22 October 2018
[b], optimized
[c], calculated parameter

4.3 PBPK modeling of voriconazole

The antifungal voriconazole exhibits a strong inhibitory activity on several CYP enzymes, i.e. its mechanism-based autoinhibition of CYP3A4 [109]. Regarding CYP2B6 inhibition, voriconazole is known for its high interaction potential with the CYP2B6 substrate and inducer efavirenz [110]. As a CYP2C19 substrate and inhibitor, voriconazole also engages in CYP2C19 DDIs [111]. The voriconazole PBPK model was developed and applied for DDI predictions in a previous publication [112]. To describe a DDI cocktail study conducted by Bosilkovska et al. [25], the voriconazole model was used to predict the reported fluvoxamine-voriconazole-bupropion DDI, by implementing competitive inhibition on CYP2B6 and CYP2C19 using literature values. Drug-dependent parameters of the voriconazole model are listed in Table S4.3.

Table S4.3: Drug-dependent parameters of the voriconazole PBPK model.

Parameter	Unit	Value	Source	Reference	Description
MW	g/mol	349.30	lit.	^{a}DB00582	Molecular weight
pKa (base)	-	1.60	lit.	[113]	Acid dissociation constant
Solubility (pH)	mg/ml	3.2 (1.0)	lit.	[113]	Solubility
		2.7 (1.2)	lit.	[114]	
		0.1 (7.0)	lit.	^{a}DB00582	
logP	-	1.80	lit.	[14, 115]	Lipophilicity
fu	%	42.0	lit.	[115–117]	Fraction unbound
Specific intest. perm.	cm/s	^{b}2.71E-04	2.81E-5	[116]	Normalized to surface are
Specific organ perm.	cm/s	4.30E-05	^{c}4.30E-05	PK-Sim®	Normalized to surface are
GFR fraction	-	1.00	asm.	-	Fraction of filtered drug in the urine
EHC cont. fraction	-	1.00	asm.	-	Fraction of bile continually released
Cellular permeabilities	cm/s	PK-Sim std.	-	[1]	Permeation across cell membranes
Partition coefficients	cm/s	P. and T.	-	[115, 116]	Organ-plasma partition coefficients
CYP3A4 K_M	µmol/l	15.00	lit.	[115]	CYP3A4 Michaelis-Menten constant
CYP3A4 kcat	1/min	^{b}2.12	0.31	[115]	CYP3A4 catalytic rate constant
CYP2C19 K_M	µmol/l	3.50	lit.	[115]	CYP2C19 Michaelis-Menten constant
CYP2C19 kcat	1/min	1.19	lit.	[115]	CYP2C19 catalytic rate constant
CYP3A4 K_I	µmol/l	9.33	lit.	[109]	Conc. for half-maximal inhibition
CYP3A4 k_{inact}	1/min	^{b}0.0015	0.04	[109]	Maximum inactivation rate constant
CYP2B6 K_I	µmol/l	^{b}0.07	^{d}0.30	[111]	Conc. for half-maximal inhibition
CYP2C19 K_I	µmol/l	^{d}4.57	lit.	[111]	Conc. for half-maximal inhibition
Weibull shape	-	^{a}1.29	-	-	Shape used for Weibull
Weibull time	min	^{a}30	-	-	Time of 50% dissolved

asm., assumed; **calc.**, calculated; **conc.**, concentration; **cont.**, continuous; **CYP**, cytochrome P450; **EHC**, enterohepatic circulation; **intest.**, intestinal; **GFR**, glomerular filtration rate; **lit.**, literature; **perm.**, permeability; **P. and T.**, Poulin and Theil calculation method; **PK-Sim std.**, PK-Sim Standard calculation method; **-**, not available.
^{a}, https://go.drugbank.com/drugs/DB00582 03.12.2020
^{b}, optimized
^{c}, calculated parameter
^{d}, in vitro values corrected for binding in the assay using fraction unbound to microsomal protein [72]

4.4 Clinical studies

In Tables S4.4 and S4.5, clinical studies used for DDI model development are listed. Virtual individuals were built according to the demographics published in the respective study report. If no data on demographics were reported, a standard individual was used as described in Section 1.4.

Table S4.4: Clinical studies used for rifampicin-bupropion DDI model development.

Rifampicin application	Bupropion application	Dose gap [h]	n	Age [years]	Weight [kg]	Females [%]	Dataset	Reference
600 mg po (tab) q.d. (D1–D7)	25 mg po (Cap) s.d. (D8)	12	10	23 (20–36)	22 (19.9–24.4)	0	te	Bosilkovska 2014 [25]
600 mg po (tab) q.d. (D1–D7)	150 mg po (IR) s.d. (D8)	12	10	31 (21–40)	73 (57–84)	60	te	Kharasch 2008a [34]
600 mg po (tab) q.d. (D1–D7)	150 mg po (SR) s.d. (D8)	24	22	22.7	65	27.3	te	Chung 2011 [38]
600 mg po (tab) q.d. (D1–D10)	150 mg po (SR) s.d. (D8)	12	17	22 (19–34)	72 (53–99)	0	te	Loboz 2006 [44]

Cap, capsule (Geneva cocktail [25]); **D**, study day; **IR**, immediate release formulations; **n**, number of individuals studied; **po**, oral; **q.d.**, once daily; **s.d.**, single dose; **SR**, sustained release formulations; **tab**, tablet; **te**, test dataset. Values are given as mean, the range of values is given in brackets.

Table S4.5: Clinical studies used for fluvoxamine-voriconazole-bupropion DDI model development.

Fluvoxamine application	Voriconazole application	Bupropion application	Dose gap [h]	n	Age [years]	Females [%]	Dataset	Reference
50 mg po (tab) b.i.d.	400 mg po (tab) s.d.	25 mg po (Cap) s.d.	12 (F), 2 (F), 2 (V)	10	23 (20–36)	0	ta	Bosilkovska 2014 [25]

b.i.d., twice daily; **Cap**, capsule (Geneva cocktail [25]); **D**, study day; **F**, fluvoxamine; **n**, number of individuals studied; **po**, oral; **s.d.**, single dose; **ta**, training dataset; **tab**, tablet; **V**, voriconazole. Values are given as mean, the range of values is given in brackets.

4.5 Concentration-time profiles

The geometric means of the population predictions (n=500) are shown as solid lines and corresponding observed data as filled dots. Symbols represent the arithmetic mean values ± standard deviation, if available. The shaded areas indicate the geometric standard deviation. Details on dosing regimens, study populations and literature references are listed in Tables S4.4 and S4.5.

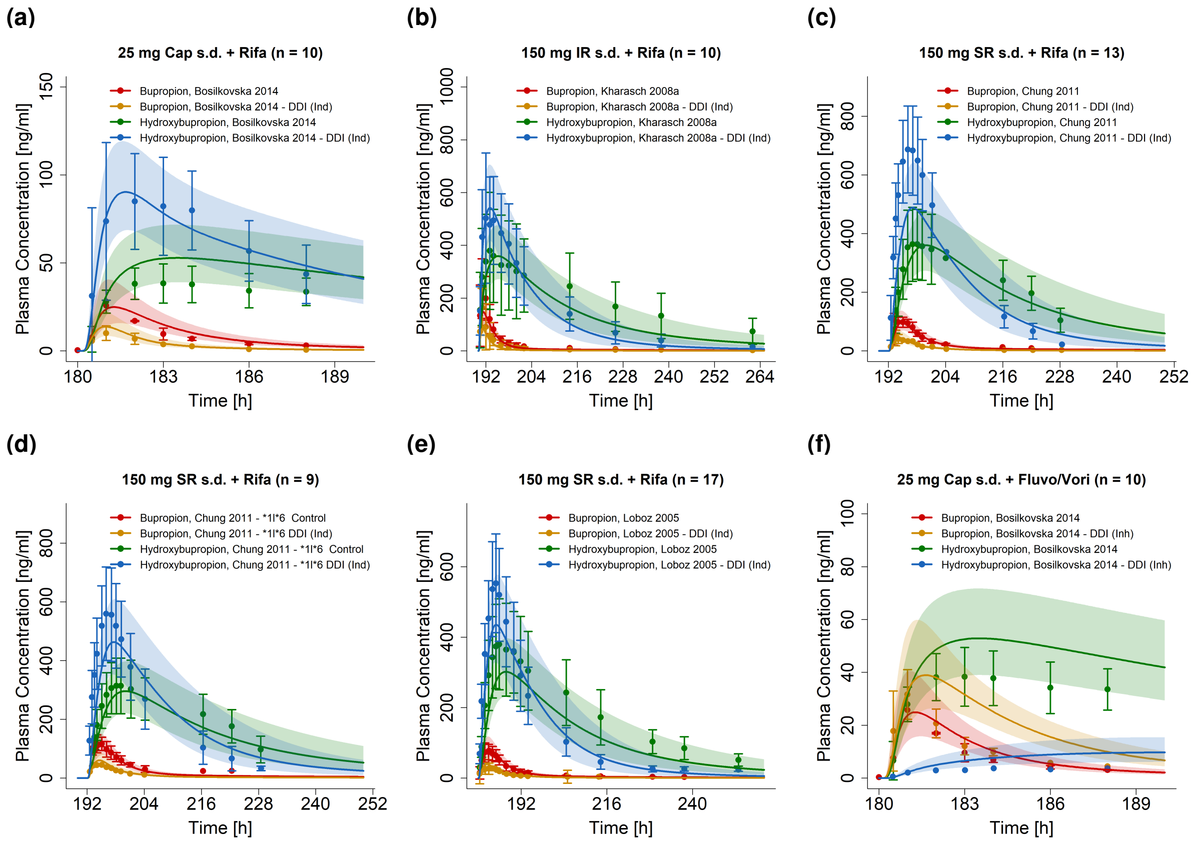

Figure S4.5.1: **Plasma concentration-time profiles of bupropion and hydroxybupropion DDI simulations** on a linear scale. **Cap**, capsule (Geneva cocktail [25]); **Control**, without perpetrator; **DDI (Ind)**, drug-drug-interaction with rifampicin as inducer; **DDI (Inh)**, drug-drug-interaction with fluvoxamine and voriconazole as inhibitors; **Fluvo/Vori**, fluvoxamine and voriconazole; **IR**, immediate release formulation; **Rifa**, rifampicin; **s.d.**, single dose; **SR**, sustained release formulation.

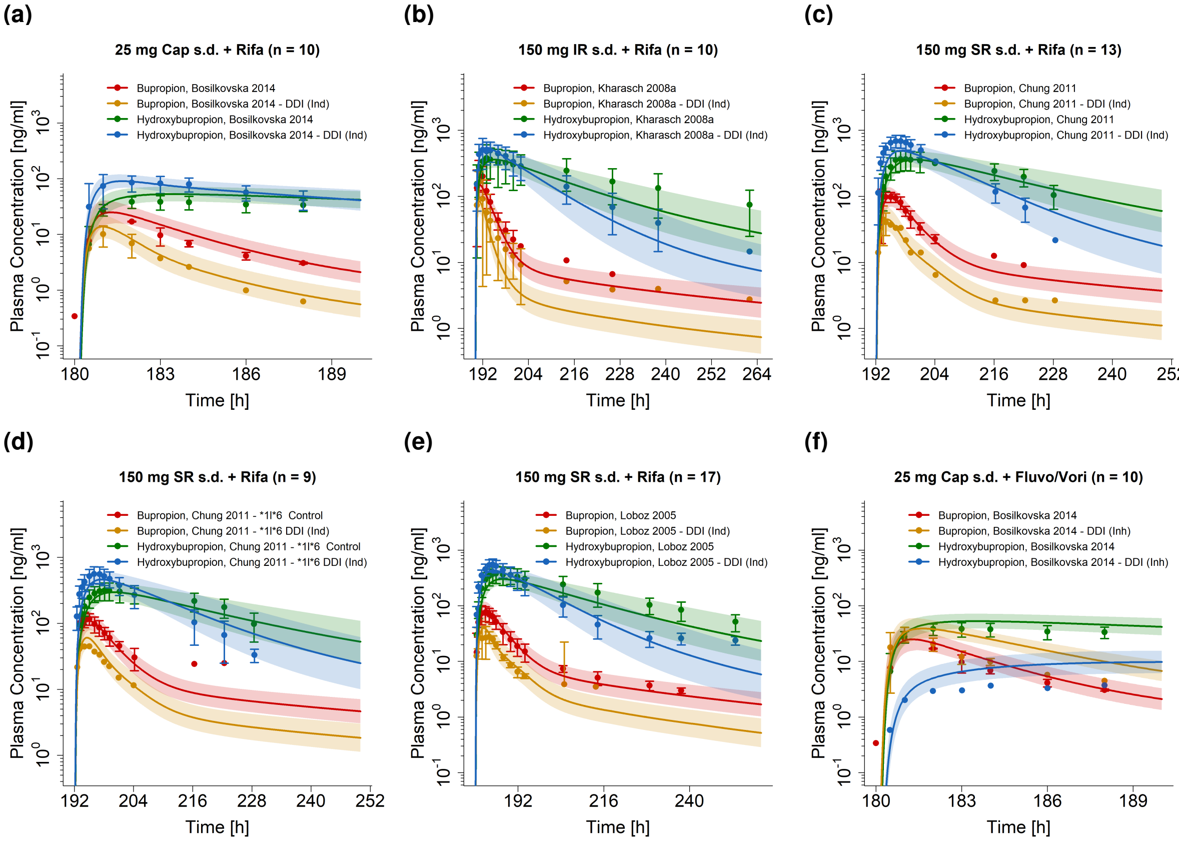

Figure S4.5.2: **Plasma concentration-time profiles of bupropion and hydroxybupropion DDI simulations** on a semi-logarithmic scale. **Cap**, capsule (Geneva cocktail [25]); **Control**, without perpetrator; **DDI (Ind)**, drug-drug-interaction with rifampicin as inducer; **DDI (Inh)**, drug-drug-interaction with fluvoxamine and voriconazole as inhibitors; **Fluvo/Vori**, fluvoxamine and voriconazole; **IR**, immediate release formulation; **Rifa**, rifampicin; **s.d.**, single dose; **SR**, sustained release formulation.

4.6 Model evaluation

4.6.1 Predicted compared to observed concentrations goodness-of-fit plots

Following, goodness-of-fit plots of predicted compared to observed plasma concentrations are illustrated in Figure S4.6.3. Details on dosing regimens, study populations and literature references are listed in Tables S4.4 and S4.5. Predicted and observed PK parameters are summarized in Table S4.7.

(a) Bupropion **(b) Hydroxybupropion**

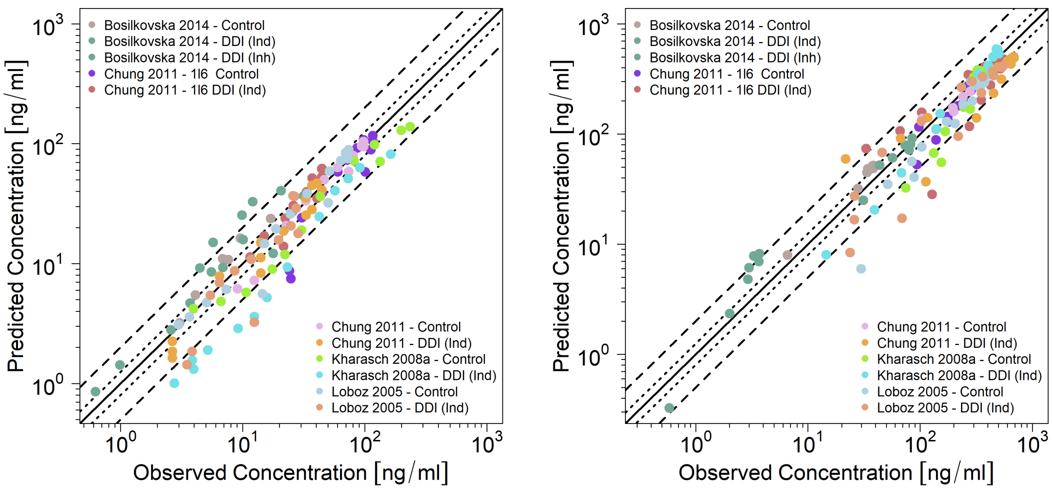

Figure S4.6.3: **DDI predicted compared to observed plasma concentrations of (a) bupropion and (b) hydroxybupropion.** The solid line marks the line of identity. Dotted lines indicate 1.25-fold, dashed lines indicate 2-fold deviation. **Control**, without perpetrators; **DDI (Ind)**, drug-drug-interaction with rifampicin as inducer; **DDI (Inh)**, drug-drug-interaction with fluvoxamine and voriconazole as inhibitors.

4.6.2 Mean relative deviation of plasma concentration predictions

Table S4.6: Mean relative deviation values of bupropion and hydroxybupropion DDI plasma concentration predictions.

Dosing	n	Compound	MRD	Compound	MRD	Dataset	Reference
			Rifampicin induction				
25 mg Cap (s.d.)	10	Bup	1.34	HBup	1.11	te	Bosilkovska 2014 Control [25]
25 mg Cap (s.d.)	10	Bup	1.65	HBup	1.07	te	Bosilkovska 2014 DDI [25]
150 mg IR (s.d.)	10	Bup	1.25	HBup	1.20	te	Kharasch 2008a Control [34]
150 mg IR (s.d.)	10	Bup	2.28	HBup	1.18	te	Kharasch 2008a DDI [34]
150 mg SR (s.d.)	13	Bup	1.15	HBup	1.04	ta	Chung 2011 **1/*1* Control [38]
150 mg SR (s.d.)	13	Bup	1.31	HBup	1.26	ta	Chung 2011 **1/*1* DDI [38]
150 mg SR (s.d.)	9	Bup	1.30	HBup	1.08	te	Chung 2011 **1/*6* Control [38]
150 mg SR (s.d.)	9	Bup	1.12	HBup	1.26	te	Chung 2011 **1/*6* DDI [38]
150 mg SR (s.d.)	18	Bup	1.30	HBup	1.35	ta	Loboz 2006 Control [44]
150 mg SR (s.d.)	18	Bup	1.57	HBup	1.26	te	Loboz 2006 DDI [44]
			Fluvoxamine and voriconazole inhibition				
25 mg Cap (s.d.)	10	Bup	1.69	HBup	2.68	ta	Bosilkovska 2014 DDI [25]
		**Mean**	**1.36 (1.04–2.68)**				
		**Median**	**1.26 (1.04–2.68)**				
			**90.90% (20/22 $\leq$ 2)**				

Bup, bupropion; **Cap**, capsule (Geneva cocktail [25]); **Control**, without perpetrators; **DDI**, drug-drug-interaction with perpetrators; **HBup**, hydroxybupropion; **IR**, immediate release formulation; **MRD**, mean relative deviation; **n**, number of individuals studied; **s.d.**, single dose; **SR**, sustained release formulation; **ta**, training dataset; **te**, test dataset.

4.6.3 AUC and C_{max} goodness-of-fit plots

Following, predicted compared to observed AUC and C_{max} values are shown for individual bupropion and hydroxybupropion profiles, their metabolite-parent AUC and C_{max} ratio and their DDI effect metabolite-parent AUC and C_{max} ratio, respectively. Details on dosing regimens, study populations and literature references are listed in Tables S4.4 and S4.5. Predicted and observed PK parameters are summarized in Tables S4.7, S4.8 and S4.9.

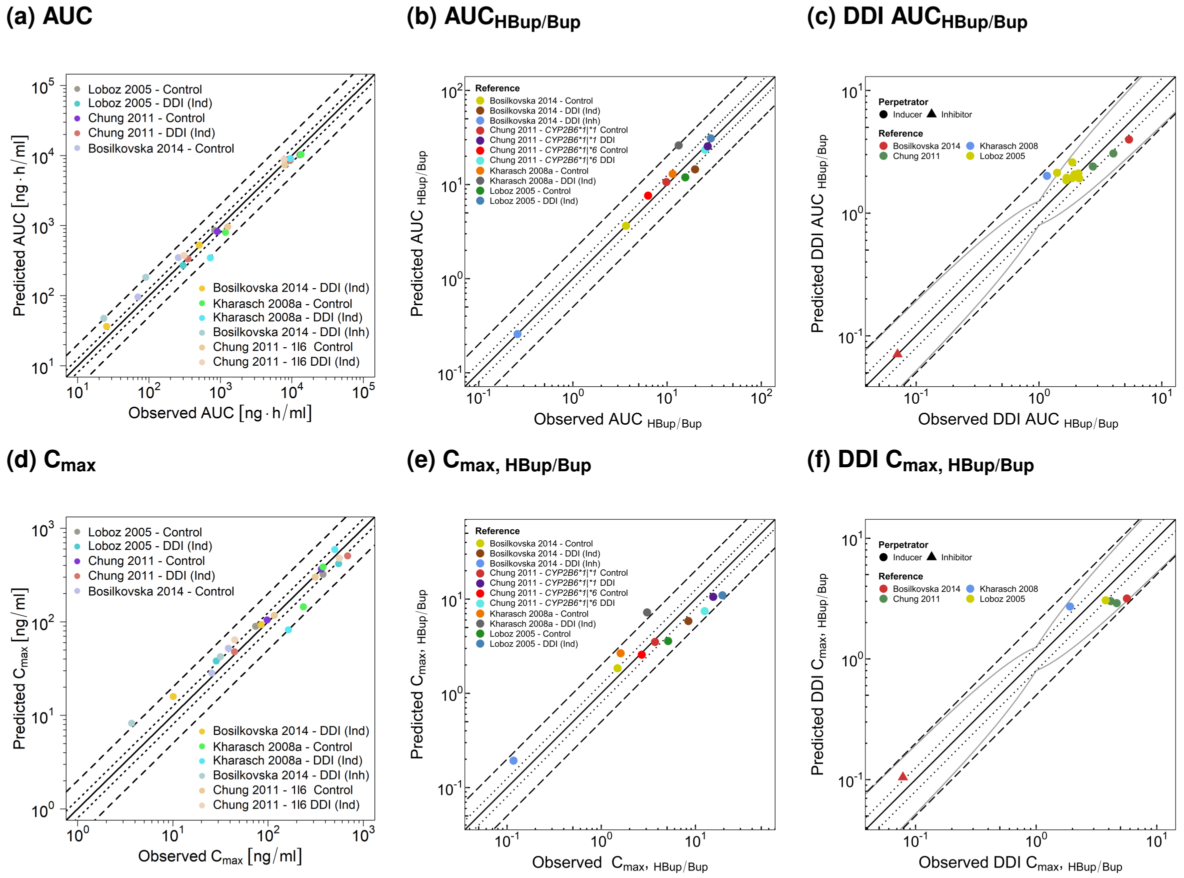

Figure S4.6.4: **Predicted compared to observed (a) AUC values, (b) $AUC_{HBup/Bup}$ ratios, (c) DDI $AUC_{HBup/Bup}$, (d) C_{max} values, (e) $C_{max,\ HBup/Bup}$ ratios and (f) DDI $C_{max,\ HBup/Bup}$ ratios.** The solid line marks the line of identity. Dotted lines indicate 1.25-fold, dashed lines indicate 2-fold deviation. The curved gray lines show the prediction success limits suggested by Guest et al. allowing a 1.25-fold variability [77]. **AUC**, area under the plasma concentration-time curve; **Bup**, bupropion; **C_{max}**, maximum plasma concentration; **Control**, without perpetrator; **DDI**, drug-drug-interaction with perpetrators; **DDI (Ind)**, drug-drug-interaction with rifampicin as inducer; **DDI (Inh)**, drug-drug-interaction with fluvoxamine and voriconazole as inhibitors; **HBup**, hydroxybupropion.

4.6.4 Geometric mean fold error of predicted AUC and C_{max} values, $AUC_{HBup/Bup}$ and $C_{max, HBup/Bup}$ ratios, and DDI $AUC_{HBup/Bup}$ and DDI $C_{max, HBup/Bup}$ ratios

Table S4.7: Predicted and observed AUC_{last} and C_{max} values of bupropion and hydroxybupropion DDI plasma concentrations.

Dosing	n	Compound	AUC_{last} pred [ng*h/ml]	AUC_{last} obs [ng*h/ml]	AUC_{last} pred/obs	C_{max} pred [ng/ml]	C_{max} obs [ng/ml]	C_{max} pred/obs	Dataset	Reference
					Rifampicin induction					
25 mg Cap (s.d.)	10	Bup	95.61	70.34	1.36	28.20	25.74	1.10	te	Bosilkovska 2014 Control [25]
25 mg Cap (s.d.)	10	Bup	47.87	25.49	1.88	18.71	10.10	1.85	te	Bosilkovska 2014 DDI [25]
150 mg IR (s.d.)	10	Bup	798.66	1182.40	0.68	144.23	235.94	0.61	te	Kharasch 2008a Control [34]
150 mg IR (s.d.)	10	Bup	345.35	722.93	0.48	81.81	164.01	0.50	te	Kharasch 2008a DDI [34]
150 mg SR (s.d.)	13	Bup	822.18	909.01	0.90	104.29	97.73	1.07	ta	Chung 2011 **1/*1* Control [38]
150 mg SR (s.d.)	13	Bup	334.43	351.52	0.95	47.74	44.49	1.07	ta	Chung 2011 **1/*1* DDI [38]
150 mg SR (s.d.)	9	Bup	958.22	1268.88	0.76	116.76	116.63	1.00	te	Chung 2011 **1/*6* Control [38]
150 mg SR (s.d.)	9	Bup	354.09	309.15	1.15	61.23	44.66	1.37	te	Chung 2011 **1/*6* DDI [38]
150 mg SR (s.d.)	18	Bup	854.58	832.02	1.03	89.31	74.04	1.21	te	Loboz 2006 Control [44]
150 mg SR (s.d.)	18	Bup	269.42	300.93	0.90	37.95	28.60	1.33	te	Loboz 2006 DDI [44]
25 mg Cap (s.d.)	10	HBup	348.31	257.69	1.35	52.07	38.33	1.36	te	Bosilkovska 2014 Control [25]
25 mg Cap (s.d.)	10	HBup	449.48	505.31	0.89	75.50	84.97	0.89	te	Bosilkovska 2014 DDI [25]
150 mg IR (s.d.)	10	HBup	10351.70	13530.75	0.77	383.99	379.17	1.01	te	Kharasch 2008 Control [34]
150 mg IR (s.d.)	10	HBup	9004.53	9608.04	0.94	591.45	503.13	1.18	te	Kharasch 2008 DDI [34]
150 mg SR (s.d.)	13	HBup	8738.64	8936.26	0.98	366.16	363.64	1.01	ta	Chung 2011 **1/*1* Control [38]
150 mg SR (s.d.)	13	HBup	8533.25	9498.00	0.90	504.90	687.07	0.74	ta	Chung 2011 **1/*1* DDI [38]
150 mg SR (s.d.)	9	HBup	7304.46	7976.41	0.92	300.13	314.03	0.96	te	Chung 2011 **1/*6* Control [38]
150 mg SR (s.d.)	9	HBup	8894.42	7838.55	1.13	492.24	59.91	0.88	te	Chung 2011 **1/*6* DDI [38]
150 mg SR (s.d.)	18	HBup	10199.30	12976.72	0.79	320.63	379.78	0.84	ta	Loboz 2006 Control [44]
150 mg SR (s.d.)	18	HBup	8346.79	8767.25	0.95	416.72	552.73	0.75	te	Loboz 2006 DDI [44]
					Fluvoxamine and voriconazole inhibition					
25 mg Cap (s.d.)	10	Bup	182.88	90.52	2.02	42.28	31.64	1.34	ta	Bosilkovska 2014 DDI [25]
25 mg Cap (s.d.)	10	HBup	47.14	23.37	2.02	8.16	3.70	2.21	ta	Bosilkovska 2014 DDI [25]
		**GMFE (range)**			**1.30 (1.02–2.02)**			**1.30 (1.00–2.21)**		
		**pred/obs within twofold (range)**			**86.36%; 19/22 (0.48–2.02)**			**95.45%; 21/22 (0.50–2.21)**		

AUC_{last}, area under the plasma concentration-time curve; **Bup**, bupropion; **Cap**, capsule (Geneva cocktail [25]); **C_{max}**, maximum plasma concentration; **Control**, without perpetrators; **DDI**, drug-drug-interaction with perpetrators; **GMFE**, geometric mean fold error; **HBup**, hydroxybupropion; **IR**, immediate release formulation; **n**, number of individuals studied; **obs**, observed; **pred**, predicted; **s.d.**, single dose; **SR**, sustained release formulation; **ta**, training dataset; **te**, test dataset.

Table S4.8: Predicted and observed $AUC_{HBup/Bup}$ and $C_{max,\ HBup/Bup}$ ratios of bupropion and hydroxybupropion DDI plasma concentrations.

**Dosing**	**n**	**$AUC_{HBup/Bup}$ pred**	**$AUC_{HBup/Bup}$ obs**	**$AUC_{HBup/Bup}$ pred/obs**	**$C_{max,\ HBup/Bup}$ pred**	**$C_{max,\ HBup/Bup}$ obs**	**$C_{max,\ HBup/Bup}$ pred/obs**	**Dataset**	**Reference**
				Rifampicin induction					
25 mg Cap (s.d.)	10	3.64	3.66	0.99	1.85	1.49	1.24	te	Bosilkovska 2014 Control [25]
25 mg Cap (s.d.)	10	14.50	19.82	0.73	5.84	8.41	0.70	te	Bosilkovska 2014 DDI [25]
150 mg IR (s.d.)	10	12.96	11.44	1.13	2.66	1.61	1.66	ta	Kharasch 2008a Control [34]
150 mg IR (s.d.)	10	26.07	13.29	1.96	7.29	3.07	2.36	te	Kharasch 2008a DDI [34]
150 mg SR (s.d.)	13	10.62	9.83	1.08	3.51	3.72	0.94	ta	Chung 2011 *1/*1 Control [38]
150 mg SR (s.d.)	13	7.62	6.29	1.21	2.57	2.69	0.95	ta	Chung 2011 *1/*1 DDI [38]
150 mg SR (s.d.)	9	24.25	27.02	0.90	9.89	15.44	0.64	te	Chung 2011 *1/*6 Control [38]
150 mg SR (s.d.)	9	23.36	25.36	0.92	7.45	12.54	0.59	te	Chung 2011 *1/*6 DDI [38]
150 mg SR (s.d.)	18	11.93	15.60	0.76	3.59	5.13	0.70	ta	Loboz 2006 Control [44]
150 mg SR (s.d.)	18	30.98	29.13	1.06	10.98	19.33	0.57	te	Loboz 2006 DDI [44]
150 mg SR (s.d.)	6	11.76	18.50	0.64	-	-	-	te	Loboz 2006 *1/*1 Control [44]
150 mg SR (s.d.)	6	21.78	30.90	0.70	-	-	-	te	Loboz 2006 *1/*1 DDI [44]
150 mg SR (s.d.)	1	22.75	25.40	0.90	-	-	-	te	Loboz 2006 *1/*4 Control [44]
150 mg SR (s.d.)	1	45.56	47.50	0.96	-	-	-	te	Loboz 2006 *1/*4 DDI [44]
150 mg SR (s.d.)	1	15.47	32.70	0.47	-	-	-	te	Loboz 2006 *1/*5 Control [44]
150 mg SR (s.d.)	1	32.36	45.90	0.71	-	-	-	te	Loboz 2006 *1/*5 DDI [44]
150 mg SR (s.d.)	6	8.38	14.50	0.58	-	-	-	te	Loboz 2006 *1/*6 Control [44]
150 mg SR (s.d.)	6	17.32	28.20	0.61	-	-	-	te	Loboz 2006 *1/*6 DDI [44]
150 mg SR (s.d.)	1	19.58	18.00	1.09	-	-	-	te	Loboz 2006 *4/*6 Control [44]
150 mg SR (s.d.)	1	38.87	38.30	1.01	-	-	-	te	Loboz 2006 *4/*6 DDI [44]
150 mg SR (s.d.)	1	18.87	21.70	0.87	-	-	-	te	Loboz 2006 *5/*5 Control [44]
150 mg SR (s.d.)	1	39.06	45.40	0.86	-	-	-	te	Loboz 2006 *5/*5 DDI [44]
150 mg SR (s.d.)	1	4.98	8.10	0.61	-	-	-	te	Loboz 2006 *6/*6 Control [44]
150 mg SR (s.d.)	1	9.69	13.60	0.71	-	-	-	te	Loboz 2006 *6/*6 DDI [44]
				Fluvoxamine and voriconazole inhibition					
150 mg IR (s.d.)	10	0.26	0.26	1.00	0.19	0.12	1.58	ta	Bosilkovska 2014 DDI [25]
**GMFE (range)**				**1.37 (1.00–2.14)**			**1.53 (1.05–2.36)**		
**Pred/Obs within twofold (range)**				**95.83%; 24/25 (0.47–1.96)**			**90.00%; 10/11 (0.57–2.36)**		

AUC_{last}, area under the plasma concentration-time curve; **Bup**, bupropion; **Cap**, capsule (Geneva cocktail [25]); **C_{max}**, maximum plasma concentration; **Control**, without perpetrators; **DDI**, drug-drug-interaction with perpetrators; **GMFE**, geometric mean fold error; **HBup**, hydroxybupropion; **IR**, immediate release formulation; **n**, number of individuals studied; **obs**, observed; **pred**, predicted; **s.d.**, single dose; **SR**, sustained release formulation; **ta**, training dataset; **te**, test dataset; **-**, no data available.

Table S4.9: Predicted and observed DDI $AUC_{HBup/Bup}$ and DDI $C_{max,\ HBup/Bup}$ ratios of bupropion and hydroxybupropion DDI plasma concentrations.

**Dosing**	**n**	**DDI $AUC_{HBup/Bup}$ pred**	**DDI $AUC_{HBup/Bup}$ obs**	**DDI $AUC_{HBup/Bup}$ pred/obs**	**DDI $C_{max,\ HBup/Bup}$ pred**	**DDI $C_{max,\ HBup/Bup}$ obs**	**DDI $C_{max,\ HBup/Bup}$ pred/obs**	**Dataset**	**Reference**
					Rifampicin induction				
25 mg Cap (s.d.)	10	3.98	5.42	0.74	3.16	5.64	0.56	te	Bosilkovska 2014 [25]
150 mg IR (s.d.)	10	2.01	1.16	1.73	2.74	1.91	1.44	te	Kharasch 2008a [34]
150 mg SR (s.d.)	13	0.72	0.64	1.12	0.73	0.72	1.01	ta	Chung 2011 **1/*1* [38]
150 mg SR (s.d.)	9	0.96	0.94	1.03	0.75	0.81	0.93	te	Chung 2011 **1/*6* [38]
150 mg SR (s.d.)	18	2.60	1.87	1.39	3.06	3.77	0.81	te	Loboz 2006 [44]
150 mg SR (s.d.)	6	1.85	1.67	1.11	-	-	-	te	Loboz 2006 **1/*1* [44]
150 mg SR (s.d.)	1	1.93	1.87	1.03	-	-	-	te	Loboz 2006 **1/*4* [44]
150 mg SR (s.d.)	1	2.13	1.40	1.52	-	-	-	te	Loboz 2006 **1/*5* [44]
150 mg SR (s.d.)	6	2.07	1.94	1.06	-	-	-	te	Loboz 2006 **1/*6* [44]
150 mg SR (s.d.)	1	1.92	2.13	0.90	-	-	-	te	Loboz 2006 **4/*6* [44]
150 mg SR (s.d.)	1	1.10	2.09	1.01	-	-	-	te	Loboz 2006 **5/*5* [44]
150 mg SR (s.d.)	1	1.94	1.68	1.16	-	-	-	te	Loboz 2006 **6/*6* [44]
					Fluvoxamine and voriconazole inhibition				
25 mg Cap (s.d.)	10	0.07	0.07	1.00	0.10	0.08	1.25	ta	Bosilkovska 2014 [25]
**GMFE (range)**				**1.23 (1.00–1.73)**			**1.46 (1.01–1.44)**		
**Pred/Obs within twofold (range)**				**100%; 13/13 (0.74–1.73)**			**100%; 6/6 (0.56–1.44)**		

AUC_{last}, area under the plasma concentration-time curve; **Bup**, bupropion; **Cap**, capsule (Geneva cocktail [25]); **C_{max}**, maximum plasma concentration; **DDI**, drug-drug-interaction with perpetrators; **GMFE**, geometric mean fold error; **HBup**, hydroxybupropion; **IR**, immediate release formulation; **n**, number of individuals studied; **obs**, observed; **pred**, predicted; **s.d.**, single dose; **SR**, sustained release formulation; **ta**, training dataset; **te**, test dataset; **-**, no data available.

5 DDGI prediction

5.1 Background

Drug-drug-gene interactions occur when subjects with variant *CYP2B6* variant genotypes receive bupropion with a potential perpetrator drug. In the following section, DDGIs were simulated for various CYP2B6 genotypes during concomitant bupropion and rifampicin intake. In the literature, plasma concentration-time profiles of this DDGI were only provided in the study of Chung et al. [38] (for the genotype *CYP2B6*1/*6* after rifampicin intake). However, Loboz et al. [44] also investigated DDGIs with rifampicin for several different genotypes and reported hydroxybupropion to bupropion AUC_{inf} ratios. Hence, for DDGI model evaluation, predicted $AUC_{inf\ HBup/Bup}$ ratios were calculated for DDGIs and compared to observed ratios. Model parameters to predict the rifampicin-bupropion DDGIs are listed in Tables S2.2 (bupropion), S3.2 (DGI) and S4.1 (rifampicin).

5.2 Clinical studies

In Table S5.1, clinical studies used for DDGI model development are listed. Virtual individuals were built according to the demographics published in the respective study reports. If no data on the demographics were reported, a standard individual was used as described in Section 1.4.

Table S5.1: Clinical studies used for DDGI model development.

**Rifampicin application**	**Bupropion appliction**	**Dose gap [h]**	**n**	***CYP2B6* genoytpe**	**Dataset**	**Reference**
600 mg po (tab) q.d. (D1–D7)	150 mg po (SR) s.d. (D8)	24	13	**1/*1*	ta	Chung 2011 [38]
600 mg po (tab) q.d. (D1–D7)	150 mg po (SR) s.d. (D8)	24	9	**1/*6*	te	Chung 2011 [38]
600 mg po (tab) q.d. (D1–D10)	150 mg po (SR) s.d. (D8)	12	6	**1/*1*	te	Loboz 2006 [44]
600 mg po (tab) q.d. (D1–D10)	150 mg po (SR) s.d. (D8)	12	1	**1/*4*	te	Loboz 2006 [44]
600 mg po (tab) q.d. (D1–D10)	150 mg po (SR) s.d. (D8)	12	1	**1/*5*	te	Loboz 2006 [44]
600 mg po (tab) q.d. (D1–D10)	150 mg po (SR) s.d. (D8)	12	1	**1/*6*	te	Loboz 2006 [44]
600 mg po (tab) q.d. (D1–D10)	150 mg po (SR) s.d. (D8)	12	6	**4/*6*	te	Loboz 2006 [44]
600 mg po (tab) q.d. (D1–D10)	150 mg po (SR) s.d. (D8)	12	1	**5/*5*	te	Loboz 2006 [44]
600 mg po (tab) q.d. (D1–D10)	150 mg po (SR) s.d. (D8)	12	1	**6/*6*	te	Loboz 2006 [44]

CYP, cytochrome P450; **D**, study day; **n**, number of individuals studied; **po**, oral; **q.d.**, once daily; **s.d.**, single dose; **SR**, sustained release formulation; **tab**, tablet; **ta**, training dataset; **te**, test dataset.

5.3 Concentration-time profiles

Observed plasma concentration-time profiles were only published in the DDGI study by Chung et al. [38]. The profiles are shown on linear and semi-logarithmic scales in Figure S5.3.1. The geometric means of the population predictions (n=500) are shown as solid lines and corresponding observed data as filled dots. Symbols represent the arithmetic mean values ± standard deviation, if available. The shaded areas indicate the geometric standard deviation.

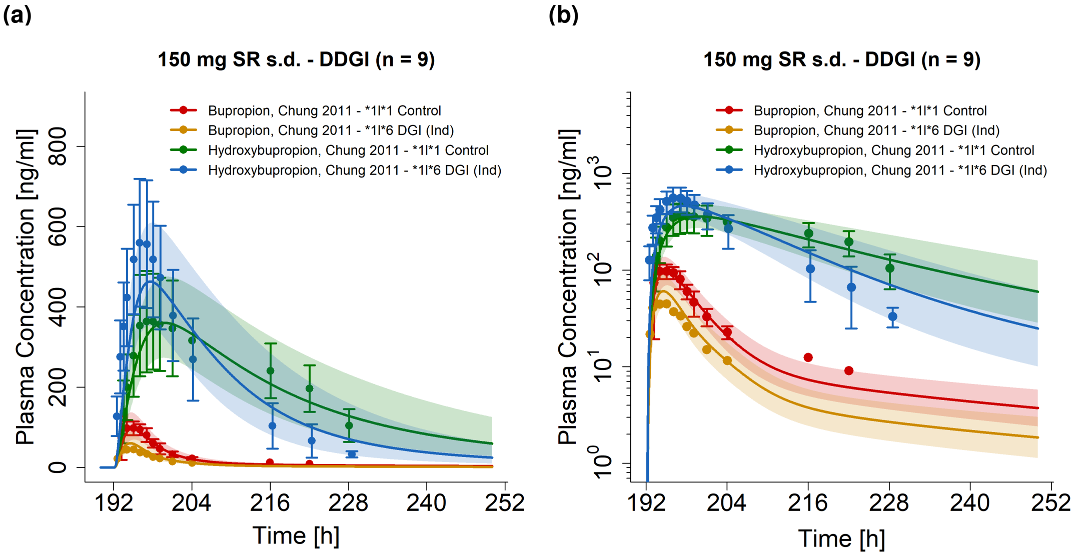

Figure S5.3.1: **Plasma concentration-time profiles of bupropion and hydroxybupropion for DDGI simulations** on (a) a linear and (b) a semi-logarithmic scale. **Control**, without perpetrator; **DDI (Ind)**, drug-drug-interaction with rifampicin as inducer; **DDGI**, drug-drug-gene-interaction; **s.d.**, single dose; **SR**, sustained release formulation.

5.4 Model evaluation

5.4.1 DDGI $AUC_{HBup/Bup}$ ratios goodness-of-fit plots

In Figure S5.4.2, predicted compared to observed DDGI $AUC_{HBup/Bup}$ ratios for different genotypes are shown. The DDGI $AUC_{HBup/Bup}$ ratios were calculated as described in Section 1.6.3. Details on dosing regimens, study populations and literature references are listed in Table S5.1. Predicted and observed PK parameters are summarized in Table S5.2.

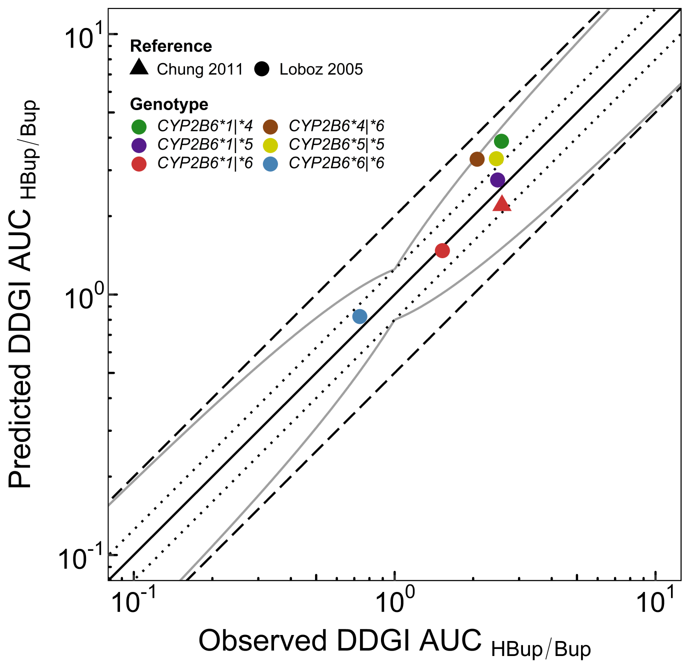

Figure S5.4.2: **Predicted compared to observed DDGI $AUC_{HBup/Bup}$ ratios.** The solid line marks the line of identity. Dotted lines indicate 1.25-fold, dashed lines indicate 2-fold deviation. The curved gray lines show the prediction success limits suggested by Guest et al. allowing a 1.25-fold variability [77]. **AUC**, area under the plasma concentration-time curve; **Bup**, bupropion; **DDGI**, drug-drug-gene-interaction; **HBup**, hydroxybupropion.

5.4.2 Geometric mean fold error of predicted DDGI $AUC_{HBup/Bup}$ ratios

Table S5.2 lists predicted and observed DDGI $AUC_{HBup/Bup}$ ratios for AUC_{last} (Chung 2011) and AUC_{inf} (Loboz 2006). Single $AUC_{HBup/Bup}$ ratios of the reference (*CYP2B6*1/*1* without perpetrator treatment) and the corresponding effect (CYP2B6 variant under perpetrator influence) are listed in Tables S3.5 and S4.8.

Table S5.2: Predicted and observed DDGI $AUC_{HBup/Bup}$ and DDGI $C_{max, HBup/Bup}$ ratios of bupropion and hydroxybupropion plasma concentrations.

Dosing	n	DDGI $AUC_{HBup/Bup}$ pred	DDGI $AUC_{HBup/Bup}$ obs	DDGI $AUC_{HBup/Bup}$ pred/obs	DDGI $C_{max, HBup/Bup}$ pred	DDGI $C_{max, HBup/Bup}$ obs	DDGI $C_{max, HBup/Bup}$ pred/obs	Dataset	Reference
150 mg SR (s.d.)	9	2.20	2.58	0.85	2.12	3.37	0.63	te	Chung 2011 **1/*6* DDI [38]
150 mg SR (s.d.)	1	3.87	2.57	1.51	-	-	-	te	Loboz 2006 **1/*4* DDI [44]
150 mg SR (s.d.)	1	2.75	2.48	1.11	-	-	-	te	Loboz 2006 **1/*5* DDI [44]
150 mg SR (s.d.)	6	1.47	1.52	0.97	-	-	-	te	Loboz 2006 **1/*6* DDI [44]
150 mg SR (s.d.)	1	3.31	2.07	1.60	-	-	-	te	Loboz 2006 **4/*6* DDI [44]
150 mg SR (s.d.)	1	3.32	2.45	1.36	-	-	-	te	Loboz 2006 **5/*5* DDI [44]
150 mg SR (s.d.)	1	0.82	0.74	1.12	-	-	-	te	Loboz 2006 **6/*6* DDI [44]
**GMFE (range)**				**1.27 (1.08–1.60)**			**1.59**		
**pred/obs within twofold (range)**				**100%; 7/7 (0.85–1.60)**			**100%; 1/1 (0.63)**		

AUC, area under the plasma concentration-time curve; **Bup**, bupropion; **C_{max}**, maximum plasma concentration; **DDI**, drug-drug-interaction with rifampicin; **DDGI**, drug-drug-gene-interaction with rifampicin in populations with *CYP2B6* varaints; **GMFE**, geometric mean fold error; **HBup**, hydroxybupropion; **n**, number of individuals studied; **obs**, observed; **pred**, predicted; **s.d.**, single dose; **SR**, sustained release formulation; **te**, test dataset; **-**, no data available.

5.4.3 DDGI scenarios of rifampicin-bupropion interactions

The change of $AUC_{HBup/Bup}$ during the rifampicin-bupropion DDGI for all CYP2B6 genotypes implemented into the model is illustrated in Figure S5.4.3. The values on the different bars represent the % change from *CYP2B6*1/*1* control conditions, for the different genotypes, with or without rifampicin coadministration. It should be noted, that DDIs for the genotypes *CYP2B6*4/*4* and *CYP2B6*5/*6* (shaded in gray) no clinical values were available to evaluate the presented model predictions. The rifampicin-bupropion coadministration protocol of Loboz et al. was applied for all simulations (see table S5.1).

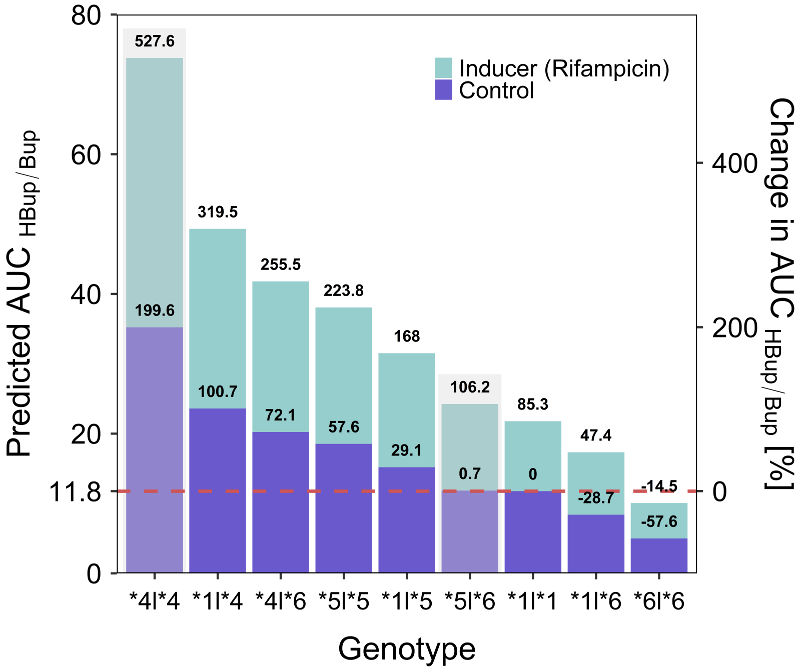

Figure S5.4.3: **Predicted $AUC_{HBup/Bup}$ for simulated DDGI scenarios. AUC**, area under the plasma concentration-time curve; **Bup**, bupropion; **Control**, without perpetrator; **HBup**, hydroxy-bupropion.

References

[1] Open Systems Pharmacology Suite Community, “Open Systems Pharmacology Suite Manual,” 2018.

[2] M. Nishimura and S. Naito, “Tissue-specific mRNA expression profiles of human phase I metabolizing enzymes except for cytochrome P450 and phase II metabolizing enzymes,” *Drug Metabolism and Pharmacokinetics*, vol. 21, no. 5, pp. 357–74, 2006.

[3] N. Kolesnikov, E. Hastings, M. Keays, O. Melnichuk, Y. A. Tang, E. Williams, M. Dylag, N. Kurbatova, M. Brandizi, T. Burdett, K. Megy, E. Pilicheva, G. Rustici, A. Tikhonov, H. Parkinson, R. Petryszak, U. Sarkans, and A. Brazma, “ArrayExpress update-simplifying data submissions,” *Nucleic Acids Research*, vol. 43, no. D1, pp. D1113–D1116, 2015.

[4] B. Prasad, K. Johnson, S. Billington, C. Lee, G. W. Chung, C. D. Brown, E. J. Kelly, J. Himmelfarb, and J. D. Unadkat, “Abundance of drug transporters in the human kidney cortex as quantified by quantitative targeted proteomics,” *Drug Metabolism and Disposition*, vol. 44, no. 12, pp. 1920–1924, 2016.

[5] National Center for Biotechnology Information (NCBI), “Expressed Sequence Tags (EST) from UniGene,” 2019.

[6] M. Meyer, S. Schneckener, B. Ludewig, L. Kuepfer, and J. Lippert, “Using expression data for quantification of active processes in physiologically based pharmacokinetic modeling,” *Drug metabolism and disposition: the biological fate of chemicals*, vol. 40, pp. 892–901, may 2012.

[7] D. Scotcher, S. Billington, J. Brown, C. R. Jones, C. D. Brown, A. Rostami-Hodjegan, and A. Galetin, “Microsomal and cytosolic scaling factors in dog and human kidney cortex and application for in vitro-in vivo extrapolation of renal metabolic clearance,” *Drug Metabolism and Disposition*, vol. 45, no. 5, pp. 556–568, 2017.

[8] J. Valentin, “Basic Anatomical and Physiological Data for Use in Radiological Protection: Reference Values,” *Annals of the ICRP*, vol. 32, jan 2002.

[9] G. Tanaka and H. Kawamura, “Anatomical and physiological characteristics for Asian reference man: Male and female of different ages: Tanaka model.,” 1996.

[10] S. Palovaara, O. Pelkonen, J. Uusitalo, and K. Laine, “Inhibition of cytochrome P450 2B6 activity by hormone replacement therapy and oral contraceptive as measured by bupropion hydroxylation,” *Clinical Pharmacology & Therapeutics*, vol. 74, no. 4, pp. 326–333, 2003.

[11] L. Fan, J. C. Wang, F. Jiang, Z. R. Tan, Y. Chen, Q. Li, W. Zhang, G. Wang, H. P. Lei, D. L. Hu, D. Wang, and H. H. Zhou, “Induction of cytochrome P450 2B6 activity by the herbal medicine baicalin as measured by bupropion hydroxylation,” *European Journal of Clinical Pharmacology*, vol. 65, no. 4, pp. 403–409, 2009.

[12] L. C. Gao, X. Huang, Z. R. Tan, L. Fan, and H. H. Zhou, “The effects of sodium ferulate on the pharmacokinetics of bupropion and its active metabolite in healthy men,” *European Review for Medical and Pharmacological Sciences*, vol. 16, no. 9, pp. 1192–1196, 2012.

[13] L. C. Gao, F. Q. Liu, L. Yang, L. Cheng, H. Y. Dai, R. Tao, S. P. Cao, D. Wang, and J. Tang, "The P450 oxidoreductase (POR) rs2868177 and cytochrome P450 (CYP) 2B6*6 polymorphisms contribute to the interindividual variability in human CYP2B6 activity," *European Journal of Clinical Pharmacology*, vol. 72, no. 10, pp. 1205–1213, 2016.

[14] W.-J. Qin, W. Zhang, Z.-Q. Liu, X.-P. Chen, Z.-R. Tan, D.-L. Hu, D. Wang, L. Fan, and H.-H. Zhou, "Rapid clinical induction of bupropion hydroxylation by metamizole in healthy Chinese men," *British Journal of Clinical Pharmacology*, vol. 74, pp. 999–1004, dec 2012.

[15] National Center for Health Statistics Hyattsville MD 20782., "Third National Health and Nutrition Examination Survey, (NHANES III)," tech. rep., 1997.

[16] "Open Systems Pharmacology Suite Community. Open Systems Pharmacology Suite Manual, Version 7.4, 2018. URLhttps://github.com/Open-Systems-Pharmacology/OSPSuite. Documentation/blob/master/OpenSystemsPharmacologySuite.pdf.," Available online (accessed on 27 November 2020).

[17] M. Fava, A. J. Rush, M. E. Thase, A. Clayton, S. M. Stahl, J. F. Pradko, and J. A. Johnston, "15 Years of Clinical Experience With Bupropion HCl," *The Primary Care Companion to The Journal of Clinical Psychiatry*, vol. 07, pp. 106–113, jun 2005.

[18] J. N. Connarn, S. Flowers, M. Kelly, R. Luo, K. M. Ward, G. Harrington, I. Moncion, M. Kamali, M. McInnis, M. R. Feng, V. Ellingrod, A. Babiskin, X. Zhang, and D. Sun, "Pharmacokinetics and Pharmacogenomics of Bupropion in Three Different Formulations with Different Release Kinetics in Healthy Human Volunteers," *AAPS Journal*, vol. 19, no. 5, pp. 1513–1522, 2017.

[19] V. M. Fokina, M. Xu, E. Rytting, S. Z. Abdel-Rahman, H. West, C. Oncken, S. M. Clark, M. S. Ahmed, G. D. Hankins, and T. N. Nanovskaya, "Pharmacokinetics of bupropion and its pharmacologically active metabolites in pregnancy," *Drug Metabolism and Disposition*, vol. 44, no. 11, pp. 1832–1838, 2016.

[20] J. E. Sager, L. S. Price, and N. Isoherranen, "Stereoselective metabolism of bupropion to OH-bupropion, threohydrobupropion, erythrohydrobupropion, and 49-OH-bupropion in vitro," *Drug Metabolism and Disposition*, vol. 44, no. 10, pp. 1709–1719, 2016.

[21] E. D. Kharasch and A. Crafford, "Common Polymorphisms of CYP2B6 Influence Stereoselective Bupropion Disposition," *Clinical Pharmacology and Therapeutics*, vol. 105, no. 1, pp. 142–152, 2019.

[22] J. E. Slemmer, B. R. Martin, and M. I. Damaj, "Bupropion is a nicotinic antagonist," *Journal of Pharmacology and Experimental Therapeutics*, vol. 295, no. 1, pp. 321–327, 2000.

[23] S. M. Stahl, J. F. Pradko, B. R. Haight, J. G. Modell, C. B. Rockett, and S. Learned-Coughlin, "A Review of the Neuropharmacology of Bupropion, a Dual Norepinephrine and Dopamine Reuptake Inhibitor," *The Primary Care Companion to The Journal of Clinical Psychiatry*, vol. 06, no. 04, pp. 159–166, 2004.

[24] M. Bosilkovska, C. Samer, J. Déglon, A. Thomas, B. Walder, J. Desmeules, and Y. Daali, "Evaluation of Mutual Drug-Drug Interaction within Geneva Cocktail for Cytochrome P450 Phenotyping using Innovative Dried Blood Sampling Method," *Basic & Clinical Pharmacology & Toxicology*, vol. 119, pp. 284–290, sep 2016.

[25] M. Bosilkovska, C. F. Samer, J. Déglon, M. Rebsamen, C. Staub, P. Dayer, B. Walder, J. A. Desmeules, and Y. Daali, "Geneva cocktail for cytochrome P450 and P-glycoprotein activity assessment using dried blood spots," *Clinical Pharmacology and Therapeutics*, vol. 96, no. 3, pp. 349–359, 2014.

[26] J. W. Findlay, J. Van Wyck Fleet, P. G. Smith, R. F. Butz, M. L. Hinton, M. R. Blum, and D. H. Schroeder, "Pharmacokinetics of bupropion, a novel antidepressant agent, following oral administration to healthy subjects," *European Journal of Clinical Pharmacology*, vol. 21, no. 2, pp. 127–135, 1981.

[27] C. Zahner, E. Kruttschnitt, J. Uricher, M. Lissy, M. Hirsch, S. Nicolussi, S. Krähenbühl, and J. Drewe, "No Clinically Relevant Interactions of St. John's Wort Extract Ze 117 Low in Hyperforin With Cytochrome P450 Enzymes and P-glycoprotein," *Clinical Pharmacology & Therapeutics*, vol. 106, pp. 432–440, aug 2019.

[28] L. M. Hesse, D. J. Greenblatt, L. L. von Moltke, and M. H. Court, "Ritonavir Has Minimal Impact on the Pharmacokinetic Disposition of a Single Dose of Bupropion Administered to Human Volunteers," *The Journal of Clinical Pharmacology*, vol. 46, pp. 567–576, may 2006.

[29] A. R. Masters, B. T. Gufford, J. B. L. Lu, I. F. Metzger, D. R. Jones, and Z. Desta, "Chiral Plasma Pharmacokinetics and Urinary Excretion of Bupropion and Metabolites in Healthy Volunteers," *Journal of Pharmacology and Experimental Therapeutics*, vol. 358, pp. 230–238, jun 2016.

[30] T. Yamazaki, A. Desai, R. Goldwater, D. Han, C. Howieson, S. Akhtar, D. Kowalski, C. Lademacher, H. Pearlman, D. Rammelsberg, and R. Townsend, "Pharmacokinetic Effects of Isavuconazole Coadministration With the Cytochrome P450 Enzyme Substrates Bupropion, Repaglinide, Caffeine, Dextromethorphan, and Methadone in Healthy Subjects," *Clinical Pharmacology in Drug Development*, vol. 6, pp. 54–65, jan 2017.

[31] J. Posner, A. Bye, S. Jeal, A. W. Peck, and P. Whiteman, "Alcohol and bupropion pharmacokinetics in healthy male volunteers," *European Journal of Clinical Pharmacology*, vol. 26, no. 5, pp. 627–630, 1984.

[32] J. Posner, A. Bye, K. Dean, A. W. Peck, and P. D. Whiteman, "The disposition of bupropion and its metabolites in healthy male volunteers after single and multiple doses," *European Journal of Clinical Pharmacology*, vol. 29, no. 1, pp. 97–103, 1985.

[33] W. Oberegger, O. Eradiri, F. Zhou, and P. Maes, "Patent No .: US 2006 / 0228415 A1," 2006.

[34] E. D. Kharasch, D. Mitchell, and R. Coles, "Stereoselective Bupropion Hydroxylation as an In Vivo Phenotypic Probe for Cytochrome P4502B6 (CYP2B6) Activity," *The Journal of Clinical Pharmacology*, vol. 48, pp. 464–474, apr 2008.

[35] E. D. Kharasch, D. Mitchell, R. Coles, and R. Blanco, "Rapid Clinical Induction of Hepatic Cytochrome P4502B6 Activity by Ritonavir," *Antimicrobial Agents and Chemotherapy*, vol. 52, pp. 1663–1669, may 2008.

[36] G. W. Hogeland, S. Swindells, J. C. McNabb, A. D. M. Kashuba, G. C. Yee, and C. M. Lindley, "Lopinavir/ritonavir Reduces Bupropion Plasma Concentrations in Healthy Subjects," *Clinical Pharmacology & Therapeutics*, vol. 81, pp. 69–75, jan 2007.

[37] N. L. Benowitz, A. Z. Zhu, R. F. Tyndale, D. Dempsey, and P. Jacob, "Influence of CYP2B6 genetic variants on plasma and urine concentrations of bupropion and metabolites at steady state," *Pharmacogenetics and Genomics*, vol. 23, no. 3, pp. 135–141, 2013.

[38] J. Y. Chung, J. Y. Cho, H. S. Lim, J. R. Kim, K. S. Yu, K. S. Lim, S. G. Shin, and I. J. Jang, "Effects of pregnane X receptor (NR1I2) and CYP2B6 genetic polymorphisms on the induction of bupropion hydroxylation by rifampin," *Drug Metabolism and Disposition*, vol. 39, no. 1, pp. 92–97, 2011.

[39] J. Dennison, A. Puri, S. Warrington, T. Endo, T. Adeloye, and A. Johnston, "Amenamevir: Studies of Potential CYP2C8- and CYP2B6-Mediated Pharmacokinetic Interactions With Montelukast and Bupropion in Healthy Volunteers," *Clinical Pharmacology in Drug Development*, vol. 7, no. 8, pp. 860–870, 2018.

[40] N. A. Farid, C. D. Payne, C. S. Ernest, Y. G. Li, K. J. Winters, D. E. Salazar, and D. S. Small, "Prasugrel, a new thienopyridine antiplatelet drug, weakly inhibits cytochrome P450 2B6 in humans," *Journal of Clinical Pharmacology*, vol. 48, no. 1, pp. 53–59, 2008.

[41] P.-H. Hsyu, A. Singh, T. D. Giargiari, J. A. Dunn, J. A. Ascher, and J. A. Johnston, "Pharmacokinetics of Bupropion and its Metabolites in Cigarette Smokers versus Nonsmokers," *The Journal of Clinical Pharmacology*, vol. 37, pp. 737–743, aug 1997.

[42] H.-P. Lei, W. Ji, J. Lin, H. Chen, Z.-R. Tan, D.-L. Hu, L.-J. Liu, and H.-H. Zhou, "Effects of Ginkgo biloba extract on the pharmacokinetics of bupropion in healthy volunteers," *British Journal of Clinical Pharmacology*, vol. 68, pp. 201–206, aug 2009.

[43] H. Lei, X. Yu, H. Xie, H. Li, L. Fan, L. Dai, Y. Chen, and H. Zhou, "Effect of St. John's wort supplementation on the pharmacokinetics of bupropion in healthy male Chinese volunteers," *Xenobiotica*, vol. 40, pp. 275–281, apr 2010.

[44] K. Loboz, A. Gross, K. Williams, W. Liauw, R. Day, J. Blievernicht, U. Zanger, and A. McLachlan, "Cytochrome P450 2B6 activity as measured by bupropion hydroxylation: Effect of induction by rifampin and ethnicity," *Clinical Pharmacology & Therapeutics*, vol. 80, pp. 75–84, jul 2006.

[45] B. Li, A. Nangia, C. Ming, and X. X. Cheng, "Patent No.: US 8,545,880 B2," 2013.

[46] S. M. Robertson, F. Maldarelli, V. Natarajan, E. Formentini, R. M. Alfaro, and S. R. Penzak, "Efavirenz induces CYP2B6-mediated hydroxylation of bupropion in healthy subjects," *Journal of Acquired Immune Deficiency Syndromes*, vol. 49, no. 5, pp. 513–519, 2008.

[47] M. TURPEINEN, A. TOLONEN, J. UUSITALO, J. JALONEN, O. PELKONEN, and K. LAINE, "Effect of clopidogrel and ticlopidine on cytochrome P450 2B6 activity as measured by bupropion hydroxylation," *Clinical Pharmacology & Therapeutics*, vol. 77, pp. 553–559, jun 2005.

[48] M. Turpeinen, N. Koivuviita, A. Tolonen, P. Reponen, S. Lundgren, J. Miettunen, K. Metsärinne, A. Rane, O. Pelkonen, and K. Laine, "Effect of renal impairment on the pharmacokinetics of bupropion and its metabolites," *British Journal of Clinical Pharmacology*, vol. 64, pp. 165–173, aug 2007.

[49] M. Turpeinen, J. Uusitalo, T. Lehtinen, M. Kailajärvi, O. Pelkonen, J. Vuorinen, P. Tapanainen, C. Stjernschantz, R. Lammintausta, and M. Scheinin, "Effects of Ospemifene on Drug Metabolism Mediated by Cytochrome P450 Enzymes in Humans in Vitro and in Vivo," *International Journal of Molecular Sciences*, vol. 14, pp. 14064–14075, jul 2013.

[50] R. Kustra, B. Corrigan, J. Dunn, B. Duncan, and P. H. Hsyu, “Lack of effect of cimetidine on the pharmacokinetics of sustained-release bupropion.,” *Journal of Clinical Pharmacology*, vol. 39, pp. 1184–8, nov 1999.

[51] W. Oberegger, P. Maes, M. Ashty Saleh, and G. Jackson, “Patent No.: US 7,645,802 B2,” 2010.

[52] Y. Schmid, A. Rickli, A. Schaffner, U. Duthaler, E. Grouzmann, C. M. Hysek, and M. E. Liechti, “Interactions between Bupropion and 3,4-Methylenedioxymethamphetamine in Healthy Subjects,” *Journal of Pharmacology and Experimental Therapeutics*, vol. 353, pp. 102–111, apr 2015.

[53] J. Woodcock, M. Khan, and L. X. Yu, “Withdrawal of Generic Budeprion for Nonbioequivalence,” *New England Journal of Medicine*, vol. 367, pp. 2461–2463, dec 2012.

[54] N. Paiement, P. K. Noonan, M. A. González, and H. Zerbe, “Steady State Plasma Levels of Bupropion After Administration of 3x150 Mg Extended Release Reference Tablets and Switching to 1x450 Mg Extended Release 450ER Tablets,” *International Journal of Clinical Pharmacology & Toxicology*, vol. 1, pp. 26–31, 2012.

[55] “ChemAxon Bupropion: https://chemicalize.com/app/calculation/bupropion,” Available online (accessed on 27 November 2020).

[56] “ChemAxon Hydroxybupropion: https://chemicalize.com/app/calculation/Hydroxybupropion,” Available online (accessed on 27 November 2020).

[57] “ChemAxon Erythro- and Threohydrobupropion: https://chemicalize.com/app/calculation/CC(NC(C)(C)C)C(O)C1%3
Available online (accessed on 27 November 2020).

[58] T. Takayanagi, D. Itoh, and H. Mizugushi, “Analysis of Acid Dissociation Equilibrium of Bupropion by Capillary Zone Electrophoresis After the Heat-Degradation,” *Chromatography*, vol. 37, no. 3, pp. 105–109, 2016.

[59] P. Muralidhar, E. Bhargav, and B. Srinath, “Formulation and optimization of bupropion HCl in microspongesby 2^3 factorial design,” *International Journal of Pharmaceutical Sciences and Research*, vol. 8, no. 3, pp. 1134–1144, 2017.

[60] “PubChem Hydroxybupropion: https://pubchem.ncbi.nlm.nih.gov/compound/446,” Available online (accessed on 27 November 2020).

[61] C. Xue, X. Zhang, and W. Cai, “Prediction of Drug-Drug Interactions with Bupropion and Its Metabolites as CYP2D6 Inhibitors Using a Physiologically-Based Pharmacokinetic Model,” *Pharmaceutics*, vol. 10, p. 1, dec 2017.

[62] M. J. Reese, R. M. Wurm, K. T. Muir, G. T. Generaux, L. St. John-Williams, and D. J. Mcconn, “An in Vitro Mechanistic Study to Elucidate the Desipramine/Bupropion Clinical Drug-Drug Interaction,” *Drug Metabolism and Disposition*, vol. 36, pp. 1198–1201, jul 2008.

[63] R. Kawai, M. Lemaire, J. L. Steimer, A. Bruelisauer, W. Niederberger, and M. Rowland, “Physiologically based pharmacokinetic study on a cyclosporin derivative, SDZ IMM 125.,” *Journal of pharmacokinetics and biopharmaceutics*, vol. 22, pp. 327–65, oct 1994.

[64] L. M. Berezhkovskiy, “Volume of Distribution at Steady State for a Linear Pharmacokinetic System with Peripheral Elimination,” *Journal of Pharmaceutical Sciences*, vol. 93, pp. 1628–1640, jun 2004.

[65] X. Wang, D. R. Abdelrahman, O. L. Zharikova, S. L. Patrikeeva, G. D. Hankins, M. S. Ahmed, and T. N. Nanovskaya, “Bupropion metabolism by human placenta,” *Biochemical Pharmacology*, vol. 79, pp. 1684–1690, jun 2010.

[66] C. Xu, E. T. Ogburn, Y. Guo, and Z. Desta, “Effects of the CYP2B6*6 allele on catalytic properties and inhibition of CYP2B6 in vitro: Implication for the mechanism of reduced efavirenz metabolism and other CYP2B6 substrates in vivo,” *Drug Metabolism and Disposition*, vol. 40, no. 4, pp. 717–725, 2012.

[67] Y. Chen, H. F. Liu, L. Liu, K. Nguyen, E. B. Jones, and A. J. Fretland, “The in vitro metabolism of bupropion revisited: Concentration dependent involvement of cytochrome P450 2C19,” *Xenobiotica*, vol. 40, no. 8, pp. 536–546, 2010.

[68] F. I. Carroll, B. E. Blough, P. Abraham, A. C. Mills, J. A. Holleman, S. A. Wolckenhauer, A. M. Decker, A. Landavazo, K. T. McElroy, H. A. Navarro, M. B. Gatch, and M. J. Forster, “Synthesis and biological evaluation of bupropion analogues as potential pharmacotherapies for cocaine addiction,” *Journal of Medicinal Chemistry*, vol. 52, no. 21, pp. 6768–6781, 2009.

[69] H. R. Arias, F. Gumilar, A. Rosenberg, K. M. Targowska-Duda, D. Feuerbach, K. Jozwiak, R. Moaddel, I. W. Wainer, and C. Bouzat, “Interaction of Bupropion with Muscle-Type Nicotinic Acetylcholine Receptors in Different Conformational States,” *Biochemistry*, vol. 48, pp. 4506–4518, jun 2009.

[70] U. Simonsen, S. Comerma-Steffensen, and K. E. Andersson, “Modulation of Dopaminergic Pathways to Treat Erectile Dysfunction,” *Basic and Clinical Pharmacology and Toxicology*, vol. 119, pp. 63–74, 2016.

[71] B. T. Gufford, J. B. L. Lu, I. F. Metzger, D. R. Jones, and Z. Desta, “Stereoselective glucuronidation of bupropion metabolites in vitro and in vivo,” *Drug Metabolism and Disposition*, vol. 44, no. 4, pp. 544–553, 2016.

[72] R. P. Austin, P. Barton, S. L. Cockroft, M. C. Wenlock, and R. J. Riley, “The Influence of Nonspecific Microsomal Binding on Apparent Intrinsic Clearance, and Its Prediction from Physicochemical Properties,” *Drug Metabolism and Disposition*, vol. 30, pp. 1497–1503, dec 2002.

[73] F. Langenbucher, “Linearization of dissolution rate curves by the Weibull distribution.,” *The Journal of Pharmacy and Pharmacology*, vol. 24, pp. 979–81, dec 1972.

[74] M. Bosilkovska, C. F. Samer, J. Déglon, M. Rebsamen, C. Staub, P. Dayer, B. Walder, J. A. Desmeules, and Y. Daali, “Geneva Cocktail for Cytochrome P450 and P-Glycoprotein Activity Assessment Using Dried Blood Spots,” *Clinical Pharmacology & Therapeutics*, vol. 96, pp. 349–359, sep 2014.

[75] Z. Desta, R. S. Gammal, L. Gong, M. Whirl-Carrillo, A. H. Gaur, C. Sukasem, J. Hockings, A. Myers, M. Swart, R. F. Tyndale, C. Masimirembwa, O. F. Iwuchukwu, S. Chirwa, J. Lennox, A. Gaedigk, T. E. Klein, and D. W. Haas, “Clinical Pharmacogenetics Implementation Consortium (CPIC) Guideline for CYP2B6 and Efavirenz-Containing Antiretroviral Therapy,” *Clinical Pharmacology and Therapeutics*, vol. 106, no. 4, pp. 726–733, 2019.

[76] P. F. Wang, A. Neiner, and E. D. Kharasch, “Stereoselective Bupropion Hydroxylation by Cytochrome P450 CYP2B6 and Cytochrome P450 Oxidoreductase Genetic Variants,” *Drug Metabolism and Disposition: The Biological Fate of Chemicals*, vol. 48, no. 6, pp. 438–445, 2020.

[77] E. J. Guest, L. Aarons, J. B. Houston, A. Rostami-Hodjegan, and A. Galetin, "Critique of the two-fold measure of prediction success for ratios: application for the assessment of drug-drug interactions.," *Drug Metabolism and Disposition: The Biological Fate of Chemicals*, vol. 39, pp. 170–3, feb 2011.

[78] N. Hanke, S. Frechen, D. Moj, H. Britz, T. Eissing, T. Wendl, and T. Lehr, "PBPK models for CYP3A4 and P-gp DDI prediction: a modeling network of rifampicin, itraconazole, clarithromycin, midazolam, alfentanil and digoxin - Supplementary document," *CPT: Pharmacometrics & Systems Pharmacology*, aug 2018.

[79] L. Kovar, C. Schräpel, D. Selzer, Y. Kohl, R. Bals, M. Schwab, and T. Lehr, "Physiologically-based pharmacokinetic (Pbpk) modeling of buprenorphine in adults, children and preterm neonates," *Pharmaceutics*, vol. 12, no. 6, pp. 1–22, 2020.

[80] D. Türk, N. Hanke, S. Wolf, S. Frechen, T. Eissing, T. Wendl, M. Schwab, and T. Lehr, "Physiologically based pharmacokinetic models for prediction of complex CYP2C8 and OATP1B1 (SLCO1B1) drug–drug–gene interactions: a modeling network of gemfibrozil, repaglinide, pioglitazone, rifampicin, clarithromycin and itraconazole," *Clinical Pharmacokinetics*, p. Supplementary document, may 2019.

[81] M. J. O'Neil, P. E. Heckelman, C. B. Koch, K. J. Roman, C. M. Kenny, and M. R. D'Arecca, *The Merck Index: An Encyclopedia of Chemicals, Drugs, and Biologicals 14th edn.* 2006.

[82] G. Baneyx, N. Parrott, C. Meille, A. Iliadis, and T. Lavé, "Physiologically based pharmacokinetic modeling of CYP3A4 induction by rifampicin in human: influence of time between substrate and inducer administration," *European Journal of Pharmaceutical Sciences : Official Journal of the European Federation for Pharmaceutical Sciences*, vol. 56, pp. 1–15, 2014.

[83] R. Panchagnula, I. Gulati, M. Varma, and Y. A. Raj, "Dissolution methodology for evaluation of rifampicin-containing fixed-dose combinations using biopharmaceutic classification sytem based approach," *Clinical Research and Regulatory Affairs*, vol. 24, no. 2-4, pp. 61–76, 2007.

[84] S. Agrawal and R. Panchagnula, "Implication of biopharmaceutics and pharmacokinetics of rifampicin in variable bioavailability from solid oral dosage forms," *Biopharmaceutics & Drug Disposition*, vol. 26, no. 8, pp. 321–34, 2005.

[85] G. Boman and V. A. Ringberger, "Binding of rifampicin by human plasma proteins," *European Journal of Clinical Pharmacology*, vol. 7, no. 5, pp. 369–73, 1974.

[86] D. S. Wishart, "DrugBank: a comprehensive resource for in silico drug discovery and exploration," *Nucleic Acids Research*, vol. 34, pp. D668–D672, jan 2006.

[87] I. E. Templeton, J. B. Houston, and A. Galetin, "Predictive utility of in vitro rifampin induction data generated in fresh and cryopreserved human hepatocytes, Fa2N-4, and HepaRG cells," *Drug Metabolism and Disposition: The Biological Fate of Chemicals*, vol. 39, no. 10, pp. 1921–9, 2011.

[88] U. Loos, E. Musch, J. C. Jensen, G. Mikus, H. K. Schwabe, and M. Eichelbaum, "Pharmacokinetics of oral and intravenous rifampicin during chronic administration," *Klinische Wochenschrift*, vol. 63, pp. 1205–11, dec 1985.

[89] T. Rodgers, D. Leahy, and M. Rowland, "Physiologically based pharmacokinetic modeling 1: predicting the tissue distribution of moderate-to-strong bases.," *Journal of Pharmaceutical Sciences*, vol. 94, pp. 1259–76, jun 2005.

[90] T. Rodgers and M. Rowland, “Physiologically based pharmacokinetic modelling 2: predicting the tissue distribution of acids, very weak bases, neutrals and zwitterions.,” *Journal of Pharmaceutical Sciences*, vol. 95, pp. 1238–57, jun 2006.

[91] A. Nakajima, T. Fukami, Y. Kobayashi, A. Watanabe, M. Nakajima, and T. Yokoi, “Human arylacetamide deacetylase is responsible for deacetylation of rifamycins: Rifampicin, rifabutin, and rifapentine,” *Biochemical Pharmacology*, vol. 82, no. 11, pp. 1747–1756, 2011.

[92] R. G. Tirona, B. F. Leake, A. W. Wolkoff, and R. B. Kim, “Human organic anion transporting polypeptide-C (SLC21A6) is a major determinant of rifampin-mediated pregnane X receptor activation.,” *The Journal of Pharmacology and Experimental Therapeutics*, vol. 304, pp. 223–8, jan 2003.

[93] A. Collett, J. Tanianis-Hughes, D. Hallifax, and G. Warhurst, “Predicting P-glycoprotein effects on oral absorption: correlation of transport in Caco-2 with drug pharmacokinetics in wild-type and mdr1a(-/-) mice in vivo.,” *Pharmaceutical Research*, vol. 21, pp. 819–26, may 2004.

[94] M. Shou, M. Hayashi, Y. Pan, Y. Xu, K. Morrissey, L. Xu, and G. L. Skiles, “Modeling, prediction, and in vitro in vivo correlation of CYP3A4 induction,” *Drug metabolism and Disposition: The Biological Fate of Chemicals*, vol. 36, no. 11, pp. 2355–70, 2008.

[95] B. Greiner, M. Eichelbaum, P. Fritz, H. P. Kreichgauer, O. von Richter, J. Zundler, and H. K. Kroemer, “The role of intestinal P-glycoprotein in the interaction of digoxin and rifampin,” *The Journal of Clinical Investigation*, vol. 104, no. 2, pp. 147–53, 1999.

[96] A. Ramamoorthy, Y. Liu, S. Philips, Z. Desta, H. Lin, C. Goswami, A. Gaedigk, L. Li, D. A. Flockhart, and T. C. Skaar, “Regulation of microRNA expression by rifampin in human hepatocytes,” *Drug Metabolism and Disposition*, vol. 41, no. 10, pp. 1763–1768, 2013.

[97] H. Zhang, C. Sridar, C. Kenaan, H. Amunugama, D. P. Ballou, and P. F. Hollenberg, “Polymorphic variants of cytochrome P450 2B6 (CYP2B6.4-CYP2B6.9) exhibit altered rates of metabolism for bupropion and efavirenz: A charge-reversal mutation in the K139E variant (CYP2B6.8) impairs formation of a functional cytochrome P450-reductase complex,” *Journal of Pharmacology and Experimental Therapeutics*, vol. 338, no. 3, pp. 803–809, 2011.

[98] M. G. Soars, D. M. Petullo, J. A. Eckstein, S. C. Kasper, and S. A. Wrighton, “An assessment of UDP-glucuronosyltransferase induction using primary human hepatocytes,” *Drug Metabolism and Disposition*, vol. 32, no. 1, pp. 140–148, 2004.

[99] M. Hirano, K. Maeda, Y. Shitara, and Y. Sugiyama, “Drug-drug interaction between pitavastatin and various drugs via OATP1B1,” *Drug Metabolism and Disposition*, vol. 34, no. 7, pp. 1229–1236, 2006.

[100] M. L. Reitman, X. Chu, X. Cai, J. Yabut, R. Venkatasubramanian, S. Zajic, J. A. Stone, Y. Ding, R. Witter, C. Gibson, K. Roupe, R. Evers, J. A. Wagner, and A. Stoch, “Rifampin’s acute inhibitory and chronic inductive drug interactions: experimental and model-based approaches to drug-drug interaction trial design,” *Clinical Pharmacology and Therapeutics*, vol. 89, no. 2, pp. 234–42, 2011.

[101] Y. Shimokawa, N. Yoda, S. Kondo, Y. Yamamura, Y. Takiguchi, and K. Umehara, “Inhibitory Potential of Twenty Five Anti-tuberculosis Drugs on CYP Activities in Human Liver Microsomes,” *Biological & Pharmaceutical Bulletin*, vol. 38, no. 9, pp. 1425–1429, 2015.

[102] J. F. Rajaonarison, M. Placidi, and B. Lacarelle, “Screening in Human Liver Interactions for Inhibitors,” *Drug Metabolism and Disposition*, vol. 20, no. 4, 1994.

[103] H. Britz, N. Hanke, A.-K. Volz, O. Spigset, M. Schwab, T. Eissing, T. Wendl, S. Frechen, and T. Lehr, “PBPK models for CYP1A2 DDI prediction: a modelling network of fluvoxamine, theophylline, caffeine, rifampicin and midazolam,” *CPT: Pharmacometrics & Systems Pharmacology*, vol. XX, pp. 1–12, 2019.

[104] R. S. Foti, P. W. Swaan, J. Wang, H. Duan, Y. Pan, and T. Hu, “Potent and Selective Inhibition of Plasma Membrane Monoamine Transporter by HIV Protease Inhibitors,” *Drug Metabolism and Disposition*, vol. 43, no. 11, pp. 1773–1780, 2015.

[105] D. Hallifax and J. B. Houston, “Saturable Uptake of Lipophilic Amine Drugs into Isolated Hepatocytes: Mechanisms and Consequences for Quantitative Clearance Prediction,” *Drug Metabolism and Disposition*, vol. 35, pp. 1325–1332, aug 2007.

[106] V. Claassen, “Review of the animal pharmacology and pharmacokinetics of fluvoxamine.,” *British Journal of Clinical Pharmacology*, vol. 15, no. 3 S, pp. 349S–355S, 1983.

[107] W. Schmitt, “General approach for the calculation of tissue to plasma partition coefficients,” *Toxicology in Vitro*, vol. 22, pp. 457–467, mar 2008.

[108] M. Miura and T. Ohkubo, “Identification of human cytochrome P450 enzymes involved in the major metabolic pathway of fluvoxamine,” *Xenobiotica*, vol. 37, no. 2, pp. 169–179, 2007.

[109] X. Li, S. Frechen, D. Moj, T. Lehr, M. Taubert, C. hsuan Hsin, G. Mikus, P. J. Neuvonen, K. T. Olkkola, T. I. Saari, and U. Fuhr, “A Physiologically Based Pharmacokinetic Model of Voriconazole Integrating Time-Dependent Inhibition of CYP3A4, Genetic Polymorphisms of CYP2C19 and Predictions of Drug–Drug Interactions,” *Clinical Pharmacokinetics*, vol. 59, no. 6, pp. 781–808, 2020.

[110] P. F. Wang, A. Neiner, and E. D. Kharasch, “Efavirenz metabolism: Influence of polymorphic CYP2B6 variants and stereochemistry,” *Drug Metabolism and Disposition*, vol. 47, no. 10, pp. 1195–1205, 2019.

[111] S. Jeong, P. D. Nguyen, and Z. Desta, “Comprehensive in vitro analysis of voriconazole inhibition of eight cytochrome P450 (CYP) enzymes: Major effect on CYPs 2B6, 2C9, 2C19, and 3A,” *Antimicrobial Agents and Chemotherapy*, vol. 53, no. 2, pp. 541–551, 2009.

[112] X. Li, S. Frechen, D. Moj, T. Lehr, M. Taubert, C. hsuan Hsin, G. Mikus, P. J. Neuvonen, K. T. Olkkola, T. I. Saari, and U. Fuhr, “A Physiologically Based Pharmacokinetic Model of Voriconazole Integrating Time-Dependent Inhibition of CYP3A4, Genetic Polymorphisms of CYP2C19 and Predictions of Drug–Drug Interactions,” *Clinical Pharmacokinetics*, vol. 59, no. 6, pp. 781–808, 2020.

[113] Pfizer Canada ULC, “Product monograph: VFEND® voriconazole Tablets 50 mg and 200 mg For Injection 200 mg / Vial (10 mg/mL when reconstituted) Powder for Oral Suspension 3 g / bottle (40 mg/mL when reconstituted) Kirkland, Quebec; 2020.,” 2020.

[114] “Scientific Discussion-VFEND Procedure No. EMEA/H/ C/387/X/09. [London]: London; 2004.,” no. September, 2004.

[115] B. Damle, M. V. Varma, and N. Wood, “Pharmacokinetics of Voriconazole Administered Concomitantly with Fluconazole and Population-Based Simulation for Sequential Use,” *Antimicrobial Agents and Chemotherapy*, vol. 55, pp. 5172–5177, nov 2011.

[116] N. R. Zane and D. R. Thakker, “A Physiologically Based Pharmacokinetic Model for Voriconazole Disposition Predicts Intestinal First-pass Metabolism in Children,” *Clinical Pharmacokinetics*, vol. 53, pp. 1171–1182, dec 2014.

[117] F. Qi, L. Zhu, N. Li, T. Ge, G. Xu, and S. Liao, “Influence of different proton pump inhibitors on the pharmacokinetics of voriconazole,” *International Journal of Antimicrobial Agents*, vol. 49, pp. 403–409, apr 2017.
